# Supplementary material for: Construction of human activity-based phosphorylation networks
Source: Mol Syst Biol. 2013 Apr 2;9:655. doi: 10.1038/msb.2013.12 (PMC3658267; doi:10.1038/msb.2013.12)
Supplement: Supplementary Information [file msb201312-s1.pdf]

Supplementary Information

Construction of Human Activity-Based Phosphorylation Networks

Robert H. Newman*, Jianfei Hu*, Hee-Sool Rho*, Zhi Xie*, Crystal Woodard, John Neiswinger, Christopher Cooper, Matthew Shirley, Hillary M. Clark, Shaohui Hu, Woochang Hwang, Jun Seop Jeong, George Wu, Jimmy Lin, Xinxin Gao, Qiang Ni, Renu Goel, Shuli Xia, Hongkai Ji, Kevin N. Dalby, Morris J. Birnbaum, Philip A. Cole, Stefan Knapp, Alexey G. Ryazanov, Donald J. Zack, Seth Blackshaw, Tony Pawson, Anne-Claude Gingras, Stephen Desiderio, Akhilesh Pandey, Benjamin E. Turk, Jin Zhang#, Heng Zhu#, Jiang Qian#

TABLE OF CONTENTS

1. SUPPLEMENTARY METHODS 4

1.1 KINASE PURIFICATION 4

1.1.1 Cloning 4

1.1.2 Kinase expression, purification and activity 4

1.2 HUMAN PROTEIN KINASE ASSAY USING PROTEIN MICROARRAYS 5

1.3 PROTEIN MICROARRAY DATA ANALYSIS 6

1.4 BAYESIAN APPROACH TO ENRICH FOR PHYSIOLOGICALLY RELEVANT EVENTS 7

1.4.1 Rationale 7

1.4.2 Data sources 8

1.4.3 Bayesian approach 8

1.4.4 Implementation 9

1.4.5 Independence of features 11

1.4.6 Generation and evaluation of the refKSR dataset 11

1.5 FIRST-STAGE KSR VALIDATION 13

1.5.1 Plasmid construction, isolation and mammalian cell transfection 13

1.5.2 SDS-PAGE and immunoblot analysis 14

1.6 SECOND-STAGE KSR VALIDATION 14

1.6.1 Rational 14

1.6.2 Transfection and pharmacological activation/inhibition 15

1.6.3 Cell lysis and immunoprecipitation **15**

1.7 M3: A STRATEGY TO IDENTIFY KINASE PHOSPHORYLATION MOTIFS **17**

1.7.1 Classification of kinases specificity **17**

1.7.2 Experimental verification of phosphorylation sites **18**

1.7.3 Prediction of phosphorylation motifs by an iterative algorithm **18**

1.7.4 Evaluation **20**

1.8 CONSTRUCTION AND EVALUATION OF A HIGH-RESOLUTION MAP OF HUMAN PHOSPHORYLATION NETWORKS **21**

1.8.1 Construction of high-resolution map **21**

1.8.2 Experimental validation **21**

1.9 CHARACTERIZATION OF PKA AS A MISSING LINK BETWEEN BTK AND ARID3A DURING B CELL RECEPTOR SIGNALING **24**

1.9.1 Identification of ARID3A S333 and S353 as PKA phosphorylation sites **24**

1.9.2 Identification of PKA Y331 as Btk phosphorylation site **25**

1.9.3 Effect of Btk-mediated phosphorylation on PKA activity *in vitro* **26**

1.9.4 Btk-mediated phosphorylation of endogenous PKA following BCR activation **26**

1.9.5 Effects of BCR activation on PKA activity **27**

1.9.6 PKA-mediated phosphorylation of ARID3A following BCR activation **28**

1.9.7 Effect of PKA inhibition on Ca^{2+} dynamics following BCR activation **28**

2. REFERENCES 30

3. SUPPLEMENTARY FIGURES 32

4. SUPPLEMENTARY TABLES 48

5. SUPPLEMENTARY FILES 51

1. SUPPLEMENTARY METHODS

1.1 KINASE PURIFICATION

1.1.1 Cloning. 289 non-redundant human kinase genes obtained from the Invitrogen Ultimate Human ORF collection and other sources were cloned into the yeast expression vector, pEGH-A, using the Gateway cloning system (Invitrogen). Each clone was verified by restriction digestion.

1.1.2 Kinase expression, purification and activity. Each kinase was expressed as a GST fusion in the budding yeast, *Saccharomyces cerevisciae*, and purified using glutathione-sepharose affinity chromatography, as described previously (Zhu et al., 2001). Examination of a random subset of purified kinases using a battery of analytical assays, including immunoblot, silver stain and autoradiographic analyses, indicated that the purification was of high quality and that the purified human kinases were free of contaminating kinase activity from yeast (Supplementary Figure 1).

To evaluate the enzymatic activity of each sample, auto- and *trans*-phosphorylation reactions were performed in the presence of [γ-^{32}P]ATP and a set of general kinase substrates (i.e., a mixture of histone H3, myelin basic protein (MBP), and casein) using a standard liquid-phase assay (Supplementary Figure 2). Comparison of the resulting signal intensities with those obtained from commercially-available MAPK3/Erk1 protein kinase and a negative control (i.e., reaction mix in the absence kinase proteins) demonstrated that > 80 % of the purified kinases exhibited readily detectable kinase activity, including MAPKs. It has been well established that while many kinases are activated via auto-phosphorylation, some kinases, especially the MAPKs, require upstream trans-phosphorylation for full activity. One plausible

explanation is that the yeast MAPKKs might have served as the upstream activating kinases for the human MAPK, because the infrastructure of the MAP signaling pathways is highly conserved between yeast and humans.

1.2 Human protein kinase assay using protein microarrays

Human protein microarrays were generated as described previously (Hu et al, 2009). Each microarray contained 4,191 unique proteins consisting of a collection of human transcription factors (1,371 proteins in total), transcriptional co-regulators (238), RNA-binding proteins (689), DNA-binding proteins (257), DNA repair proteins (146), chromosome organizing proteins (286), protein kinases (332) and mitochondrial proteins (652) as well as a panel of proteins involved in various other cellular processes (589). To identify in vitro substrates for each kinase, a protocol similar to that described by Zhu et al. was used (Zhu et al, 2001). Briefly, protein microarrays were first blocked with 2% BSA in Tris-buffered saline (TBS) for 1 hour at room temperature before being incubated with the purified kinase-of-interest in reaction buffer (25 mM Hepes at pH 7.5 with 100 mM NaCl, 50 mM Tris-HCl pH 7.5, 55 nM ATPγ^{32}P, 1 mM DTT, 10 mM $MgCl_2$, 1 mM $MnCl_2$, 1 mM EGTA, 1 mM $NaVO_4$, 1 mM NaF, and 0.1 % NP-40) for 30 minutes at 30 ^{o}C. The microarray was then washed three times each in 1) TBS, 0.1% Tween-20 (TBST) and 2) 0.5% SDS before being rinsed briefly with double-distilled H_2O and dried by centrifugation. Each slide was then exposed to BioMax high resolution X-ray film (Kodak) for 16 hours and 5 days. Finally, developed films were scanned using an office scanner at a resolution of 4,800 dpi. Each captured image obtained from autoradiographic films was processed using Photoshop. The substrate profiling on each image was

acquired using GenePix software (Axon) and positive hits were identified as described below.

1.3 PROTEIN MICROARRAY DATA ANALYSIS

The process of data analysis includes four steps: 1) background correction, 2) normalization, 3) identification of positive hits (i.e., phosphorylation events), and 4) removal of autophosphorylated proteins (i.e., non-specific interactions). The first step, background correction, is critical to reduce background noise. To accomplish this, we implemented both local and neighboring background correction and compared the two approaches based on their ability to recover known KSRs. Because local background correction recovered known KSRs most efficiently, this method was used to correct background noise in this study. Next, we quantified the signal intensity of each protein on the array by dividing the median foreground intensity by the median intensity of its local background. Normalization is important in this process because it helps reduce spatial artifacts on the microarrays that might arise from uneven mixing, uneven washing, and/or uneven drying of the slide. Given the fact that proteins are randomly printed on the microarray and only a small portion of the proteins are potential substrates for a given kinase, we assumed that the median signal intensity of each block on the microarray should be close to one (i.e., the foreground intensity should be close to the background intensity for most spots). Suppose the original signal intensity of a protein is I_{ori}, the median signal intensity of the block that the protein locates is I_{median}, the corrected signal intensity of the protein after within-chip normalization is I_{norm}, then $I_{norm} = I_{ori} – I_{median} +1$. To identify positive hits on the microarrays, we estimated the standard deviation from the signal intensity distribution, as described in detail previously (Hu et al, 2009). A cutoff of

three standard deviations above the mean was chosen in this study. Since each protein was printed in duplicate, a protein was scored as a positive hit only if both spots of the same protein showed signal intensity higher than a cutoff value determined for that particular microarray experiment. Finally, because it is impossible to distinguish signals arising from autophosphorylation events and *trans*-phosphorylation events, we conducted negative control experiments during each set of assays to identify proteins that underwent autophosphorylation on the protein microarrays. Those proteins that exhibited autophosphorylation activity in the negative control experiments were excluded from the substrate list for further analysis.

1.4 BAYESIAN APPROACH TO ENRICH FOR PHYSIOLOGICALLY RELEVANT EVENTS

1.4.1 Rationale. To predict KSRs that are likely to occur in vivo, we performed a Bayesian analysis from diverse types of biological datasets that support functional relationships among kinase/substrate pairs. Specifically, we used information about tissue-specific gene expression, subcellular localization, and protein-protein interactions (PPIs). We assumed that kinase-substrate pairs meeting one or more of the following conditions are more likely to represent physiologically relevant interactions: (1) pairs in which both the kinase and the substrate are expressed in the same tissue-types, (2) pairs in which both the kinase and the substrate localize to or otherwise share the same cellular compartments, and (3) pairs in which the kinase and the substrate exhibit a relatively short distance in the known PPI network. These assumptions were tested using the positive and negative datasets described below.

1.4.2 Data sources. To obtain expression profiles for kinases and substrates, we used a microarray dataset which describes gene expression profiles across 79 different human tissues (Su et al, 2004). During these analyses, the Affymetrix probeset IDs were converted to official gene symbols. If multiple probes corresponded to a unique gene symbol, the average gene expression value was used. Meanwhile, cellular localization information was retrieved from the Gene Ontology database (http://www.geneontology.org/). Finally, the PPI data were collected from multiple databases, including DIP (Database of Interacting Proteins, http://dip.doe-mbi.ucla.edu/), MIPS (Mammalian PPI database, http://mips.gsf.de/proj/ppi/), IntAct (ftp://ftp.ebi.ac.uk/pub/databases/intact/current), Biogrid (http://www.thebiogrid.org/) and HPRD (Human Protein Reference Database, http://www.hprd.org). A total of 55,047 PPI pairs were included.

1.4.3 Bayesian approach. To integrate different types of datasets, we used a naïve Bayesian approach that has been widely used for integrating data from heterogeneous sources (Hu et al, 2010; Jansen et al, 2003). A formal description about Bayesian statistics can be found in (Gelman et al). Here we only give a brief description.

We first defined the ‘positive’ and ‘negative’ datasets for purposes of predicting and validating KSRs using Bayesian network analysis. For the positive dataset, we collected 1,103 experimentally validated kinase-substrate pairs from the literature and the PhosphoELM database (phospho.elm.eu.org) (note that the comKSR dataset only includes the subset of 741 known KSRs whose kinases can be found in our kinase collection). Since very little direct information exists about negative interactions, we

constructed an artificial dataset containing 10,000 protein pairs where no kinases were included in the set.

Next, we defined the 'prior' probability of finding a true KSR as

$$P_{prior} = \frac{P(positive)}{P(negative)} = \frac{P(positive)}{1 - P(positive)} \tag{1}$$

The 'posterior' probability of finding a true KSR after considering *n* supporting evidences is

$$P_{post} = \frac{P(positive \mid f_1 \ldots f_n)}{P(negative \mid f_1 \ldots f_n)} \tag{2}$$

According to the Bayesian theory

$$P_{post} = L \times P_{prior} \tag{3}$$

where the likelihood ratio *L* is defined as

$$L = \frac{P(f_1 \ldots f_n \mid positive)}{P(f_1 \ldots f_n \mid negative)} \tag{4}$$

Because the three features (co-expression, subcellular localization, and PPI) are independent to each other (see below), *L* can be simplified to

$$L = \prod_{i=1}^{n} \frac{P(f_1 \ldots f_n \mid positive)}{P(f_1 \ldots f_n \mid negative)} \tag{5}$$

1.4.4 Implementation. We integrated three independent data sources to predict more physiologically relevant KSRs in the rawKSR dataset. For gene expression data, we computed the Pearson correlation coefficients (PCCs) for each KSR across different tissue types. We found that known KSRs tend to have higher PCCs than other random protein pairs ($P<2.2e-16$, t-test), with average PCC of 0.32 and 0.21, respectively. Using the cellular localization data, we grouped each protein into one of six localization categories: (1) nucleus, (2) cytoplasm, (3) membranes, (4) nucleus and cytoplasm, (5)

cytoplasm and membranes, and (6) nucleus, cytoplasm and membranes. For a given KSR pair there exist 36 possible subcellular localization combinations. We found that known KSR pairs are more likely to share some sub-cellular compartments than are two randomly-selected proteins. For example, 8.7% of KSR pairs share three sub-cellular compartments (nucleus, cytoplasm and membranes), and 47.0% of KSR pairs share two sub-cellular compartments (nucleus and cytoplasm), as a contrast, only 0.16% of random-selected protein pairs share three sub-cellular compartments, and 11.3% of them share two sub-cellular compartments. Finally, we used the PPI dataset to calculate the PPI distance of each KSR pair in the known PPI networks. We found that known KSRs are enriched for PPI distances of one or two compared to random protein pairs (78% vs 2.5%, $p<2.2e-16$, binomial test), where one represents direct PPI between the kinase and its substrate and two indicates that the two proteins interact with a common partner.

We then calculated the likelihood ratio (L) for each KSR in the rawKSR dataset using the variables observed from the positive and negative datasets. Because the three features are independent (see below), it is proper to apply the naïve Bayesian approach for the data integration. For each KSR pair, we obtained its overall L value based on its individual L values. The overall L value represents the likelihood that the KSR pair is physiologically relevant. The direct PPIs (d=1) dominate the top ~200 KSRs. In other words, if one kinase and one protein have direct physical interaction and show positive signal on the protein microarray, then their kinase-substrate relationship is more likely to be physiologically relevant. For the other KSRs, the contributions to the overall likelihood ratio from the three features are comparable. In these cases, the contribution from an indirect PPI (e.g., d=2 where two proteins share a common interacting partner such as a scaffolding protein) becomes notable to the L values.

1.4.5 Independence of features. Naïve Bayesian analysis requires that the features are independent of each other. Therefore, we calculated the correlation coefficients of *L* values for each pairwise combination of features. The correlation coefficients between subcellular localization and co-expression, between subcellular localization and PPI, and between co-expression and PPI, are 0.007, 0.052, and 0.022, respectively. The low correlation coefficients suggest that these three features are, indeed, independent. Therefore, the likelihood ratio can be factorized and the naïve Bayesian approach is applicable.

1.4.6 Generation and evaluation of the refKSR dataset. To generate the refKSR dataset, we tested different cutoffs for the *L* value and performed a simulation to estimate its statistical significance. It is difficult to derive a P value based on analytic approaches, because the distributions of these three features are quite different and discrete. Therefore, we used permutation to estimate the P value. To do this, we permuted the *L* values of all protein pairs for each of the three features. After this permutation, all the biological correlations inherent in the data have been disrupted, and each protein pair has no relationship with the three L values associated to it. The L value corresponding to the top 5% was selected as cutoff. In this set, 5% of protein pairs have *L* values greater than 1.555. Therefore, all KSRs in the refKSR dataset exhibited *L* values > 1.555.

To evaluate the ability of the Bayesian approach to enrich for physiologically relevant KSRs, we examined several lines of computational and experimental evidence. For instance, we compared the fraction of known substrates in the rawKSR and refKSR datasets based on MS/MS data gathered from the literature and various databases. We

also asked whether the Bayesian approach could more efficiently recover known KSRs. To this end, we collected 1,108 known KSRs from database and literature sources. Among them, 687 KSRs were not examined in this study because the kinase was not included in our collection and/or the substrate was not present on the protein microarray. If we used these 687 KSRs as a training set and the remaining 421 KSRs as a test set, we were able to recover 22 known KSRs from among the 3,656 KSRs in the refKSR dataset (22/3,656 = 0.6% of total KSRs in the set). By comparison, we were able to recover 25 known KSRs from the 24,046 KSRs in the rawKSR dataset (25/24,046 = 0.1% of total KSRs in the set). This result suggests that the known KSRs are enriched among the refKSRs. The relatively low recovery rate of known KSRs may be due to the fact the protein microarray assay does not include additional factors, such as scaffolding proteins or co-regulators, that facilitate the phosphorylation reaction.

Because our Bayesian statistics model did not include biological processes as a predictor, this information could be used as an independent assessment of the physiological-relevance of the refKSRs. For this analysis, we assumed that if the substrates of a given kinase are enriched for a particular biological process, then the kinase is also likely to be involved in that biological process. To predict the function a given kinase, we calculated the enriched GO terms (biological processes) among its substrates and asked whether we could recover the known biological processes of that kinase. A GO term was considered enriched if the false discovery rate (FDR) corrected *P* value is less than 0.01. The *P* value was calculated based on hypergeometric distribution with the proteins on the array serving as background.

1.5 First-stage KSR validation

1.5.1 Plasmid construction, isolation and mammalian cell transfection. To experimentally evaluate the refKSR dataset, a subset of 243 putative KSR pairs was randomly selected from among the refKSRs for in situ validation, as described below. Each substrate gene within the set (representing 136 unique genes) was cloned from the corresponding Entry vector into the mammalian expression vector pSG5-FLAG (kindly provided by S. Dianne Hayward) using the Gateway cloning system (Invitrogen). At the same time, each kinase gene within the set (representing 75 unique genes) was shuttled into the expression vector, pcDNA3.1-V5, via the Gateway LR reactions (Invitrogen, Inc.). All plasmids were amplified in *E. coli* DH5α and isolated by alkaline lysis. Plasmid DNA was then immobilized on glass-fiber DNA binding plates (NUNC, Inc.), washed with DNA wash buffer (10 mM Tris-HCl at pH 7.5 with 80% EtOH), and eluted in 10 mM Tris-HCl at pH 8. Following purification, all clones were verified by restriction digestion. To validate putative KSR pairs, HeLa cells were plated at ~ 0.6 x 10^4 cells/well in 96-well tissue culture plates and maintained at 37 ^oC under 5% CO_2 in Dulbecco's Modified Eagle's Media (DMEM) supplemented with 10 % fetal bovine serum (FBS), 1% penicillin/streptomycin. The following morning, cells were transfected with 200 ng of FLAG-substrate DNA in the presence of either V5-kinase DNA (200 ng) or pcDNA3.1 empty vector (200 ng) using polyethylenimine (PEI)-mediated transfection. PEI (1 mg/mL) was added at a ratio of 1 μL PEI per μg plasmid DNA. Two days after transfection, the cells were lysed in 40 μL of hot 2x SDS-Laemmli loading buffer followed by brief agitation with a sterile 96-prong replicator. A portion of each lysate (15 μL) was then subjected to western blot analysis, as described below. To ensure that the kinase-dependent changes in the substrate levels observed during the initial screen were

not due to differences in transfection efficiencies between the substrate alone and substrate plus kinase transfection mixes, the assay was repeated for a group of 24 KSRs as described above except 35 ng of pEX-GFP DNA was also included in each transfection mix. In these experiments, the GFP served as a transfection control, which could also be used to normalize substrate signal intensities during data analysis.

1.5.2 SDS-PAGE and immunoblot analysis. During the initial screen, lysates were resolved on a gradient polyacrylamide gel electrophoresis (PAGE) gel (BioRad) and transferred to nitrocellulose membranes using the iBlot semi-dry transfer system (Invitrogen). Membranes were blocked for at least 1 hour in blocking buffer (TBST, 5 % non-fat milk) before being incubated overnight at 4 ^{o}C in a primary antibody solution composed of rabbit anti-FLAG antibody (Sigma) diluted 1:1000 in blocking buffer. The following morning, membranes were washed three times in TBST, incubated for 1 hour at room temperature in goat anti-rabbit-HRP conjugate (Pierce) diluted 1:10,000 in blocking buffer, washed in TBST and treated with ECL Plus Detection Reagent (GE Healthcare). Chemiluminescence was detected using HyperECL X-Ray film (GE Healthcare). Likewise, V5-tagged kinases and GFP transfection markers were also detected using mouse monoclonal anti-V5 (Genscript) and rabbit polyclonal anti-GFP antibodies, as indicated.

1.6 SECOND-STAGE KSR VALIDATION

1.6.1 Rational. To demonstrate that those substrates that exhibit kinase-dependent changes in their electrophoretic mobility and/or protein levels in the first-round validation are, in fact, specifically phosphorylated by the identified kinase, several substrates of

PKA, PKC, Akt, and Erk1, were selected for further analysis (Supplementary Table 2). These kinases were selected because 1) the endogenous kinase can be pharmacologically activated and/or inhibited in a specific manner inside cells and 2) phospho-substrate antibodies, raised against the consensus phosphorylation motif of each, are commercially available (Cell Signaling Technologies).

1.6.2 Transfection and pharmacological activation/inhibition. Cells were transiently transfected with 1.0 μg of FLAG-substrate DNA in six-well culture dishes using PEI-mediated transfection as outlined above. In order to minimize differences in transfection efficiency between wells, all wells for a given substrate were transfected using a common transfection mix. Two days after transfection, endogenous levels of the kinase-of-interest were activated and/or inhibited as indicated in Figure 2. The assays were conducted in the following cell lines for each respective kinase: PKC experiments were conducted in HeLa; Erk1 in the colon cancer cell line, HCT116; PKA in either HEK293-T or HeLa; Akt in NIH3T3. Prior to Akt activation, the cells were starved for 24 hours in serum-free media.

1.6.3 Cell lysis and immunoprecipitation. Following drug treatment, the culture media was removed and the cells were washed in ice-cold phosphate-buffered saline (PBS). To promote lysis, 100 μL of ice-cold lysis buffer (RIPA, 0.1% SDS supplemented with phosphatase inhibitor cocktails 1 & 2 (Sigma), 4 mM Na_3VO_4, 4 mM NaF, 25 nM calyculin A, 1 mM PMSF, and protease inhibitor cocktail (Roche)) was added to each well. The cells were then scraped, transferred to Eppendorf tubes and vortexed at 4 ^oC. Following lysis, cell debris was removed from the lysates by centrifugation at 15,000 x *g*

for 30 – 60 minutes at 4 ^{o}C. To ensure that equivalent amounts of lysate were used for immunoprecipitation (IP) experiments, the total protein concentration of each supernatant was determined by the bicinchonic acid (BCA) assay (Pierce). Lysates were then incubated for 2 hours at 4 ^{o}C with 10 μL (20 μL of 50% slurry) of anti-FLAG-M2-agarose beads (Sigma) suspended in lysis buffer. The final volume of each IP was brought to 400 μL by the addition of lysis buffer. To isolate FLAG-tagged proteins, the beads were pelleted at 6,000 x *g* for 30 seconds, washed 4 times with RIPA, 0.1% SDS, and eluted in 20 μL 2x SDS-Laemmli buffer. Beads were then boiled for 3 - 5 minutes and centrifuged at 8,000 x *g* for 30 seconds to remove unmelted beads. Finally, supernatants were loaded onto 4 – 12 % Bis-Tris NuPAGE gels (Invitrogen), resolved in 2-(N-morpholino)ethanesulfonic acid (MES)-SDS running buffer, transferred to nitrocellulose membranes using the iBlot semi-dry transfer system and subjected to western blot analysis, as described above. The following phospho-substrate specific antibodies were used during these experiments: 1) to detect phosphorylated PKA substrates, a p(S/T)-PKA substrate antibody raised against the motif RRXpS/pT was diluted 1:1,000 in blocking buffer; 2) to detect phosphorylated PKC substrates, a p(S)-PKC substrate antibody raised against the motif R/KXpSϕR/K was diluted 1:1,000 in blocking buffer; 3) to detect phosphorylated Akt substrates, a p(S/T) Akt substrate antibody raised against the motif RXRXXp(S/T) was diluted 1:1,000 in blocking buffer; 4) to detect phosphorylated substrates of Erk1, a p(S/T)P antibody was diluted 1:1,000 in blocking buffer. All phospho-substrate antibodies were obtained from Cell Signaling Technologies (Danvers, MA). After probing with phospho-substrate antibody, each blot was stripped in stripping buffer (67.5 mM Tris-HCl at pH 6.7 with 2% SDS and 100 mM β-mercaptoethanol) and re-probed with anti-FLAG antibody, as described above. The

intensity of each band was then measured using ImageJ software (NIH). Each phospho-substrate signal was normalized to the amount of immunoprecipiated protein, as determined from the anti-FLAG immunoblot.

1.7 M3: A STRATEGY TO IDENTIFY KINASE PHOSPHORYLATION MOTIFS

We developed an integrated algorithm, termed M3 (Motif discovery based on Microarray and MS/MS), which combines our KSR data with in vivo phosphorylation sites determined primarily by MS/MS analysis, to systematically identify phosphorylation motifs. First, 13,244 of the 70,422 known sites were mapped to 1,644 of the substrates identified in this study. The short amino acid sequences (i.e., 15-mers) containing these sites were then binned into groups according to their identified KSRs. Finally, M3 used an iterative approach to identifying statistically significant motifs within each group. In the case of dual-specificity kinases, we also separately considered motifs that contained pS/T or pY sites.

1.7.1 Classification of kinase specificity. Prior to M3 analysis, each kinase was classified as either a Ser/Thr, Tyr, or dual-specificity kinase. This was done by collecting information from two major sources: the human kinome (Manning et al, 2002) and the KEGG database (http://www.genome.jp/kegg-bin/get_htext?ko01001.keg). For those kinases not covered by these two datasets, we obtained the specificity information from their GO functional annotation. For example, GO:0004674 (protein serine/threonine kinase activity) indicates that the associated kinase phosphorylates Ser and/or Thr residues while GO:004713 (protein tyrosine kinase activity) indicates that the associated kinase phosphorylates tyrosine residues.

1.7.2 Phosphorylation sites. Experimentally verified phosphorylation sites can be classified into two classes: 1) phosphorylation sites with no known upstream kinase(s) (termed S_c sites) and 2) phosphorylation sites with a known upstream kinase (S_p sites). The S_c sites, which are based on MS/MS experiments, were collected from SwissProt annotation, HPRD, and the literature (Diella et al, 2004; Molina et al, 2007; Olsen et al, 2006; Wang et al, 2007; Yang et al, 2006). Meanwhile, the S_p sites were collected from three sources: PhosphoELM (phospho.elm.eu.org), PhosphoSitePlus (www.phosphosite.org) and HPRD (www.hprd.org). However, the number of sites that fall into this category is relatively small. In fact, 96 human kinases have no S_p sites and 55.4% of human kinases have less than 5 S_p sites. Once collected, the S_p and S_c sites were merged to obtain a non-redundant dataset covering 70,422 experimentally verified phosphorylation sites. We then mapped these phosphorylation sites to the protein sequences of the substrates identified in the protein microarray experiments.

1.7.3 Prediction of phosphorylation motifs by an iterative algorithm. To predict consensus phosphorylation motifs for the kinases in our collection, we integrated the following data sources: the rawKSRs determined by protein microarray, the phosphorylation sites determined by MS/MS (i.e., the S_c sites described above), and the phosphorylation sites linked to certain kinases in the literature (i.e., the S_p sites). Each site was mapped to the protein sequences of K_a's substrates identified in the rawKSR dataset and subject to analysis by the M3 algorithm. This algorithm takes two major steps.

Step 1: Initiation. First, a set of seed sites was constructed. We constructed a foreground positional weight matrix, M_F, based on all sites in S_p and S_c for K_a.. To prevent the interference of sites phosphorylated by other kinases, sites in S_c in proteins with many upstream kinases (>100) were excluded when we constructed M_F. In this matrix, the frequencies of amino acids are based on proteins. In other words, if multiple sites on one substrate have the same amino acid at one position, this type of amino acid is only counted once at this position. A background matrix, M_B, was also calculated from MS/MS phosphorylation sites found in all protein sequences in the human proteome. We then scanned all the MS/MS phosphorylation sites on the substrates according to a relative entropy formula

$$score(s) = \sum_{i=-7, i \neq 0}^{7} f_F(aa_i) \times \log \frac{f_F(aa_i)}{f_B(aa_i)} \tag{6}$$

where aa_i is the amino acid at position i in the phosphorylation site, and f_F (aa_i) and f_B (aa_i) are the frequency of amino acid *aa* at position *i* in the foreground and background positional weight matrices (PWM), respectively. The MS/MS phosphorylation sites were then ranked according to their scores. The top 10 best-matched sites were considered as seed sites.

Step 2: Iteration. Based on the seed sites, we constructed an initial M_F and then used this profile to scan all the remaining MS/MS sites. The site with the best score was added to the seed sites and removed from the remaining phosphorylation sites on the substrates, and then the M_F was updated. We repeated the process until the best score of the remaining sites was below a cutoff or until the number of seed sequences was equal to the number of substrates for the kinases determined by protein microarray. To determine the

score cutoff, we calculated the score distribution for a negative set of sites (i.e., the MS/MS sites mapped on the proteins not phosphorylated by K_a) with the initial M_F. The cutoff was arbitrarily determined as the score at the top 5% of the distribution. To ensure that the motifs were from multiple substrates, at most two sites were used for each substrate. Once determined, the phosphorylation motif was produced using the program, WebLogo (Crooks et al, 2004).

1.7.4 Evaluation. We compared the predicted PWMs with those obtained from peptide libraries. The PWMs were transformed into a one-dimensional vector with size of 200=20*10 (20 types of amino acids and 10 flanking positions). A similarity score is defined as the correlation coefficient between two PWMs,

$$s = \frac{\sum_{a=1}^{200} (f_1(a) - 0.05) \bullet (f_2(a) - 0.05)}{\sqrt{\sum_{a=1}^{200} (f_1(a) - 0.05)^2 \bullet \sum_{a=1}^{200} (f_2(a) - 0.05)^2}} \quad (7)$$

where f_1 and f_2 are the respective PWMs obtained from the two approaches, and 0.05 is the expected probability for each amino acid.

To evaluate the statistical significance of the similarity between two PWMs, we calculated the similarity score distribution for randomized PWMs. For a given PWM, we permuted the frequency for the 20 amino acids at each position. We then calculated the similarity score for 1,000 pairs of randomized PWMs. In the random set, only 5% of the PWM pairs exhibited a similarity score above 0.164. In contrast, a comparison between our motifs and those generated from the peptide libraries revealed that 75% of PWM pairs have a similarity score above that cutoff.

1.8 Construction and Evaluation of a High-Resolution Map of Human Phosphorylation Networks

1.8.1 Construction of high-resolution map. For each kinase with a phosphorylation motif, we scanned the motifs against the MS/MS sites mapped to the substrates in the comKSR dataset. The match score is defined as equation (6). If the match score was above the threshold, we predicted that the corresponding site is targeted by the corresponding kinase. Each kinase has a specific threshold because different phosphorylation motifs have different information content and thus, are characterized by different ranges of matching scores. For a given kinase, we scanned a set of MS/MS sites that are not located on the substrates of the kinase and obtained a distribution of the matching score. We defined the score at the top 5% of the distribution as the threshold for the motif.

We scanned 284 kinases against their corresponding substrate sets. On average we predicted 19 sites for each kinase. We recovered 758 known phosphorylation sites for these kinases, which corresponds to a prediction sensitivity of 65.6%.

1.8.2 Experimental validation. To experimentally evaluate our phosphorylation site predictions, three KSR pairs that had initially exhibited a kinase-dependent change in the stability of the substrate during the first-stage validation were selected for further analysis. These pairs, along with their predicted site of phosphorylation, are as follows: 1) PKA is predicted to phosphorylate DAXX at S688; 2) MAPK15 is predicted to phosphorylate CBL at S900; 3) PKA is predicted to phosphorylate PRKAR1A at S77. The phosphoacceptor site within each substrate gene was then mutated to Ala using QuikChange mutagenesis (Stratagene). All mutations were introduced within the gene-of-

interest in the context of the pDONR221 Entry vector (Invitrogen). The primer sets used for mutagenesis are as follows: DAXX^{S688A}, sense: 5'-CACGAGGGTGGACGCTCCCAGCCATG-3', antisense: 5'-CATGGCTGGGAGCGTCCACCCTCGTG-3'; CBL^{S900A}, sense: 5'-GAATTTGTTTCCATTTCTGCTCCTGCCCATGTAGC-3', antisense: 5'-GCTACATGGGCAGGAGCAGAAATGGAAACAAATTC-3'; PRKAR1A^{S77A}, sense: 5'-GCACTCGTACAGACGCAAGGGAGGATGAG-3', antisense: 5'-CTCATCCTCCCTTGCGTCTGTACGAGTGC-3'. All primers were obtained from Integrated DNA technologies (Coralville, IA). Following mutagenesis, each clone was verified by restriction digestion, sequenced and shuttled into the appropriate expression vector (see below) using the Gateway cloning system (Invitrogen).

To determine what effect mutation of the putative phosphorylation site has on the stability of each substrate in the presence of its cognate kinase inside cells, mutant genes were shuttled into the pSG-FLAG vector and assayed using the cell-based assay described above (see "First Stage KSR Validation" for details). During these experiments, the mutated proteins were expressed in the presence or absence of their cognate kinase in HeLa cells. As a positive control, each mutant protein's wild-type counterpart was also expressed in the presence or absence of the cognate kinase in adjacent wells. Aside from acting as a positive control, the latter set also served as a point of comparison when assessing kinase-dependent changes among the mutated substrates.

To demonstrate that the differences in the kinase-dependent changes observed between the wild-type and mutant proteins inside cells is due to decreased phosphorylation of the mutant proteins, the phosphorylation status of both the wild-type

and mutant proteins was examined in vitro in the presence of kinase and [γ-^{32}P]-ATP using a standard liquid phase phosphorylation assay. During these analyses, we focused on the PKA substrates DAXX and PRKAR1A, both of which underwent kinase-dependent stabilization and no change of the wild-type and mutant protein levels, respectively, in the cell-based assay. While wild-type PRKAR1A was purified from yeast, as described (Hu et al., 2009), DAXX purified from yeast showed low levels of phosphorylation in the absence of PKA suggesting contamination by a yeast kinase(s). Therefore, wild-type DAXX and DAXX^{S688A} were expressed and purified from bacteria. Briefly, both the wild-type and mutant genes were shuttled into the bacterial expression vector pDEST15, which encodes an N-terminal GST-fusion under control of the T7 promoter. Plasmids were then transformed into BL21(λDE3) *E. coli* and the expression of each protein was induced at an optical density between 0.6-0.8 O.D. by the addition of 1 mM isopropyl-β-D-thio-galactoside (IPTG, Sigma). Following induction, cells were incubated overnight at 16 ^{o}C under vigorous shaking before being pelleted at 3,450 x *g*. Cells were then lysed by treatment with lysis buffer containing lysozyme followed by three freeze-thaw cycles. Finally, proteins were purified by GST-affinity chromatography as described above, except no phosphatase inhibitors were used. Protein purity was > 85%, as assessed by Coomassie staining.

To measure the extent of PKA-mediated phosphorylation of the purified substrates, equivalent amounts of the wild-type and mutant proteins were incubated with the PKA catalytic subunit (New England Biolabs) in the presence of [γ-^{32}P]-ATP for 30 minutes at 30 ^{o}C. As a negative control, wild-type and DAXX^{S688A} were also incubated with GST-tagged PAK1 (EMD/Calbiochem). Following incubation, the reactions were quenched by the addition of 4X lithium dodecyl sulfate (LDS) loading buffer

supplemented with 5% βME before being resolved on a 4-12% Bis-Tris NuPAGE gel (Invitrogen) in MES/SDS running buffer. After electrophoresis, the proteins were transferred to a nitrocellulose membrane using the iBlot transfer system (Invitrogen) and exposed to Kodak BioMax high-resolution X-ray film for 16 hours (short exposure) and 4 weeks (long exposure). To ensure that equivalent amounts of protein were in each well, the blot was blocked and probed with polyclonal anti-GST antibody (Millipore), as described in Supplemental section 1.5.2. Finally, the intensity of each autoradiographic band was quantitated using Image J software.

1.9 CHARACTERIZATION OF PKA AS A MISSING LINK BETWEEN BTK AND ARID3A DURING B CELL RECEPTOR SIGNALING

1.9.1 Identification of ARID3A S333 and S353 as PKA phosphorylation sites. Based on the high resolution map, two putative PKA phosphorylation sites were identified in ARID3A: a strong consensus site at S353 (REGRRQSFGGSLF) and a looser consensus site at S333 (CEKRGLSNPNELQ). To determine which site(s) contributes to the stabilization phenotype observed for the ARID3A, each of the putative phosphorylation sites was mutated to alanine in the context of the FLAG-ARID3A vector using QuikChange mutagenesis. The following primer sets were used for mutagenesis: ARID3A^{S333A}, sense: 5'-GAGAAGCGGGGCCTCGCTAACCCCAATGAGC-3', antisense: 5'-GCTCATTGGGGTTAGCGAGGCCCCGCTTCTC-3'; ARID3A^{S353A}, sense: 5'-GCCGGCGCCAGGCCTTTGGTGGCTCC-3'; antisense: 5'-GGAGCCACCAAAGGCCTGGCGCCGGC-3'. Following mutagenesis, positive clones were verified by restriction digestion and sequenced.

Each mutant protein was then expressed in HeLa cells in the presence or absence of V5-PKA, as described above (see “First Stage KSR Validation” for details). In addition to kinase-dependent changes in stability, the extent of PKA-mediated phosphorylation was determined for each mutant protein according to the protocol described in “Second Stage KSR Validation”. For these analyses, we also evaluated the S333A/S353A double mutant to rule out the possibility that additional PKA phosphorylation sites exist in ARID3A.

1.9.2 Identification of PKA Y331 as Btk phosphorylation site. PKA residue Y331 was identified as a likely site of Btk-mediated phosphorylation based on the similarity of its surrounding sequence to the Btk phosphorylation motif, as determined by M3. To experimentally validate this residue as a site of Btk-mediated phosphorylation, Y331 was mutated to Phe in the context of pDONR221 using the GeneArt Site directed mutagenesis kit (Invitrogen). The following primer set was used for mutagenesis: sense: 5’-GATACGAGTAACTTTGACGACTTTGAGGAAGAAGAAATCCGGGTC-3’, antisense: 5’-GACCCGGATTTCTTCTTCCTCAAAGTCGTCAAAGTTACTCGTATC-3’. Following mutagenesis, positive clones were verified by restriction digestion and sequenced. Both wild type PKA (WT) and PKA^{Y331F} (MT) genes were then shuttled into the pDEST15 bacterial expression vector using the Gateway cloning system (Invitrogen), as described, and transformed into BL21(λDE3) *E. coli*. Each protein was expressed and purified by GST-affinity chromatography according to Zhu et al. (2001).

To assess the extent of Btk-mediated Tyr phosphorylation on each protein, WT and MT PKA were incubated with 140 ng of commercial Btk (Invitrogen) for 30 minutes at 30 ^{o}C, isolated by SDS-PAGE and transferred to a nitrocellulose membrane using the

iBlot transfer system. Tyr phosphorylation levels were then measured by western blotting using an anti-pTyr antibody (clone 4G10; Upstate/Millipore). Finally, the membrane was stripped and the total PKA in each lane was determined using an antibody against the PKA catalytic subunit (BD Biosciences).

1.9.3 Effect of Btk-mediated phosphorylation on PKA activity *in vitro*. Recombinant GST-PKA, purified from yeast, was first incubated in the presence or absence of commercial Btk (Invitrogen) in reaction buffer containing cold ATP. To remove Btk from the subsequent reaction, GST-PKA was then re-purified by GST-affinity chromatography before being incubated with recombinant ARID3A in the presence of [γ-^{32}P]-ATP. Despite the fact that Btk does not appear to phosphorylate ARID3A (Webb et al., 2000), the Btk inhibitor, terreic acid (10 μM), was also included in this reaction buffer to ensure that residual Btk did not contribute to ARID3A phosphorylation. The extent of ARID3A phosphorylation after 5, 10 and 20 minutes was then determined by autoradiography. The intensity of each band was measured using ImageJ software (NIH) and plotted as “percent maximum signal intensity” (i.e., the signal intensity of p-ARID3A in the “PKA + Btk” lane after 20 minutes).

1.9.4 Btk-mediated phosphorylation of endogenous PKA following BCR activation. Ramos B cells at a density of 10^6 cells/mL were incubated in the presence or absence of 20 μM terric acid (TA) for 30 minutes at 37° C. Cells were then treated with goat anti-human F(ab’)$_2$ (10 μg/mL) for 10 minutes, as indicated, washed once with ice cold PBS, and lysed in 400 μL ice cold lysis buffer (RIPA, 0.1% SDS supplemented with phosphatase inhibitor cocktails 2 & 3 (Sigma), 4 mM Na_3VO_4, 4 mM NaF, 1 mM PMSF,

and protease inhibitor cocktail (Roche)), as described above. Prior to immunoprecipitation, all lysates were pre-cleared with 10 μL (20 μL of 50% slurry) Protein A/G Plus agarose beads (Santa Cruz Biotech) for 10 minutes at 4 ^{o}C. Cleared lysates were then incubated for 1 hour at 4 ^{o}C with a polyclonal antibody against the catalytic subunit of PKA (Santa Cruz Biotech). The antibody was added at a concentration of 10 μg of antibody/mg lysate. After 1 hour, 10 μL (20 μL of 50% slurry) of Protein A/G Plus-agarose beads in lysis buffer were added to the lysate/antibody solution and incubated for an additional 1 hour at 4 ^{o}C. To isolate endogenous PKA, immune complexes were pelleted at 1,000 x *g* for 1 minute, washed 4 times with RIPA, 0.1% SDS, and eluted in 20 μL of 2x SDS-Laemmli buffer. Beads were then boiled for 3 - 5 minutes and centrifuged at 1,000 x *g* for 2 minutes to remove unmelted agarose. Finally, supernatants were loaded onto 4 – 12% Bis-Tris NuPAGE gels (Invitrogen), resolved in MES-SDS running buffer, transferred to nitrocellulose membranes using the iBlot semi-dry transfer system and subjected to western blot analysis. The extent of Tyr phosphorylation was determined using the anti-pTyr antibody (clone 4G10), described above, stripped and re-probed with a monoclonal antibody against the catalytic subunit of PKA. The intensity of each band was measured using ImageJ software (NIH). Each pTyr signal was normalized to the amount of immunoprecipiated protein, as determined from the anti-PKA immunoblot.

1.9.5 Effects of BCR activation on PKA activity. Ramos B cells at a density of 10^6 cells/mL were incubated in the presence or absence of the PKA inhibitor H89 (10 μM) for 30 minutes at 37° C. Cells were then treated with F(ab')$_2$ (10 μg/mL) for 10 minutes, as indicated, and lysed in 2x SDS-PAGE loading buffer. The lysates were resolved by

SDS-PAGE and probed with the α-phospho-(S/T) PKA substrate antibody described above. To compare the changes in PKA-mediated phosphorylation caused by $F(ab')_2$ crosslinking to those caused by global increases in cAMP levels, forskolin (Fsk, 50 μM) was substituted for $F(ab')_2$ in the assay. Actin served as a loading control.

1.9.6 PKA-mediated phosphorylation of ARID3A following BCR activation. Ramos B cells at a density of 10^6 cells/mL were incubated in the presence or absence of either H89 (10 μM) or TA (20 μM) for 30 minutes at 37° C. Cells were then treated with $F(ab')_2$ (10 μg/mL) for 10 minutes, as indicated, and lysed in non-denaturing lysis buffer (20 mM Tris, pH 8.0, 137 mM NaCl, 10% glycerol, 1% NP-40, 2 mM EDTA) supplemented with protease and phosphatase inhibitor cocktails (see above). Endogenous ARID3A was then immunoprecipitated using rabbit antisera against ARID3A (a kind gift from P. Tucker) and Protein A/G Plus agarose beads. The extent of PKA-mediated phosphorylation was then determined by western blotting using the α-phospho-(S/T) PKA substrate antibody and normalized to total ARID3A levels using either a monoclonal anti-ARID3A antibody (Sigma) or by stripping the blot and re-probing with anti-ARID3A antisera.

1.9.7 Effect of PKA inhibition on Ca^{2+} dynamics following BCR activation. Indo-1 loaded Ramos B cells at a density of 10^6 cells/mL were incubated in the presence or absence of H89 for 30 minutes at room temperature. To assess the effects of PKA inhibition on Ca^{2+} dynamics following BCR activation, cells were stimulated with $F(ab')_2$ (10 μg/mL) and intracellular Ca^{2+} levels were measured by FACS over a 10 minute time

period. Calcium levels are expressed as Indo-1 emission at 400 nm divided by Indo-1 emission at 475 nm.

2. REFERENCES

Crooks GE, Hon G, Chandonia JM, Brenner SE (2004) WebLogo: a sequence logo generator. *Genome Res* **14:** 1188-1190

Diella F, Cameron S, Gemund C, Linding R, Via A, Kuster B, Sicheritz-Ponten T, Blom N, Gibson TJ (2004) Phospho.ELM: a database of experimentally verified phosphorylation sites in eukaryotic proteins. *BMC Bioinformatics* **5:** 79

Gelman A, Carlin JB, Stern H, Rubin DB *Bayesian Data Analysis*: Chapman & Hall/CRC.

Hu J, Wan J, Hackler L, Jr., Zack DJ, Qian J (2010) Computational analysis of tissue-specific gene networks: application to murine retinal functional studies. *Bioinformatics* **26:** 2289-2297

Hu S, Xie Z, Onishi A, Yu X, Jiang L, Lin J, Rho HS, Woodard C, Wang H, Jeong JS, Long S, He X, Wade H, Blackshaw S, Qian J, Zhu H (2009) Profiling the human protein-DNA interactome reveals ERK2 as a transcriptional repressor of interferon signaling. *Cell* **139:** 610-622

Jansen R, Yu H, Greenbaum D, Kluger Y, Krogan NJ, Chung S, Emili A, Snyder M, Greenblatt JF, Gerstein M (2003) A Bayesian networks approach for predicting protein-protein interactions from genomic data. *Science* **302:** 449-453

Manning G, Whyte DB, Martinez R, Hunter T, Sudarsanam S (2002) The protein kinase complement of the human genome. *Science* **298:** 1912-1934

Molina H, Horn DM, Tang N, Mathivanan S, Pandey A (2007) Global proteomic profiling of phosphopeptides using electron transfer dissociation tandem mass spectrometry. *Proc Natl Acad Sci U S A* **104:** 2199-2204

Olsen JV, Blagoev B, Gnad F, Macek B, Kumar C, Mortensen P, Mann M (2006) Global, in vivo, and site-specific phosphorylation dynamics in signaling networks. *Cell* **127:** 635-648

Su AI, Wiltshire T, Batalov S, Lapp H, Ching KA, Block D, Zhang J, Soden R, Hayakawa M, Kreiman G, Cooke MP, Walker JR, Hogenesch JB (2004) A gene atlas of the mouse and human protein-encoding transcriptomes. *Proc Natl Acad Sci U S A* **101:** 6062-6067

Wang Y, Ding SJ, Wang W, Jacobs JM, Qian WJ, Moore RJ, Yang F, Camp DG, 2nd, Smith RD, Klemke RL (2007) Profiling signaling polarity in chemotactic cells. *Proc Natl Acad Sci U S A* **104:** 8328-8333

Yang F, Stenoien DL, Strittmatter EF, Wang J, Ding L, Lipton MS, Monroe ME, Nicora CD, Gristenko MA, Tang K, Fang R, Adkins JN, Camp DG, 2nd, Chen DJ, Smith RD (2006) Phosphoproteome profiling of human skin fibroblast cells in response to low- and high-dose irradiation. *J Proteome Res* **5:** 1252-1260

Zhu H, Bilgin M, Bangham R, Hall D, Casamayor A, Bertone P, Lan N, Jansen R, Bidlingmaier S, Houfek T, Mitchell T, Miller P, Dean RA, Gerstein M, Snyder M (2001) Global analysis of protein activities using proteome chips. *Science* **293:** 2101-2105

3. SUPPLEMENTARY FIGURES

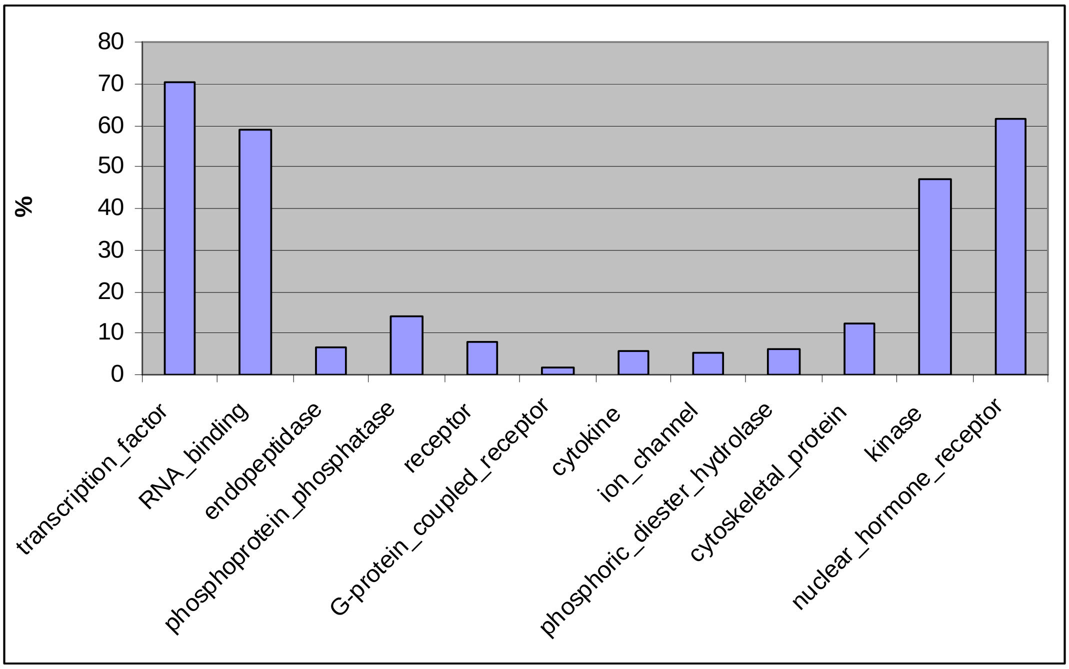

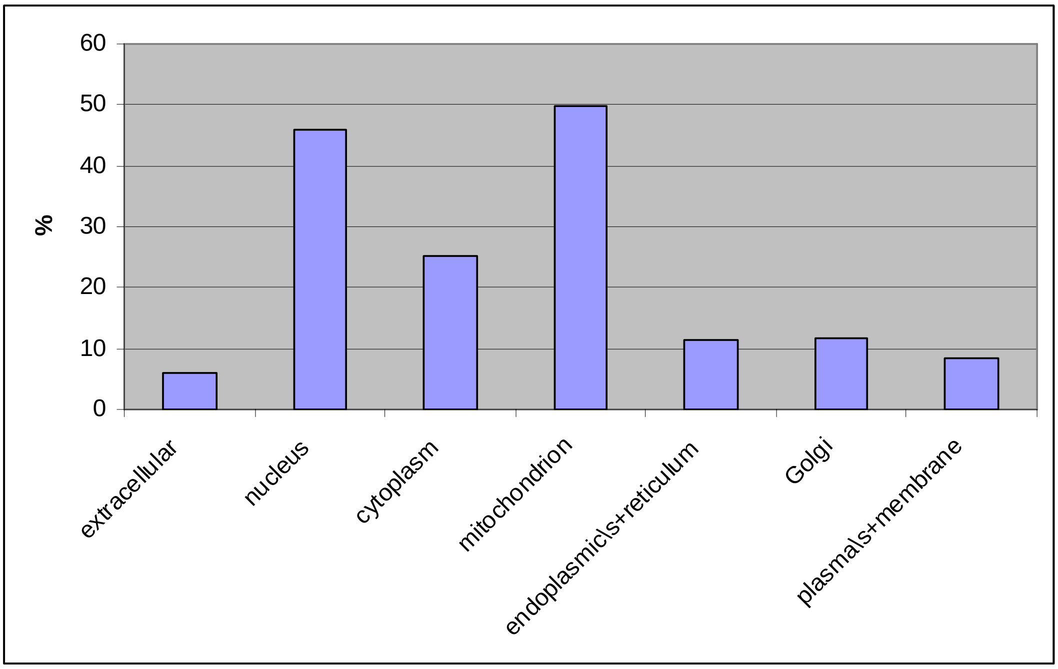

Supplementary Figure 1. The distribution of protein families (top) and cellular localizations (bottom) of proteins on the protein microarray. The percentage was computed based on the ratio between the number of the proteins in a given category on the protein microarray and the total number of such proteins in that same category in the human proteome.

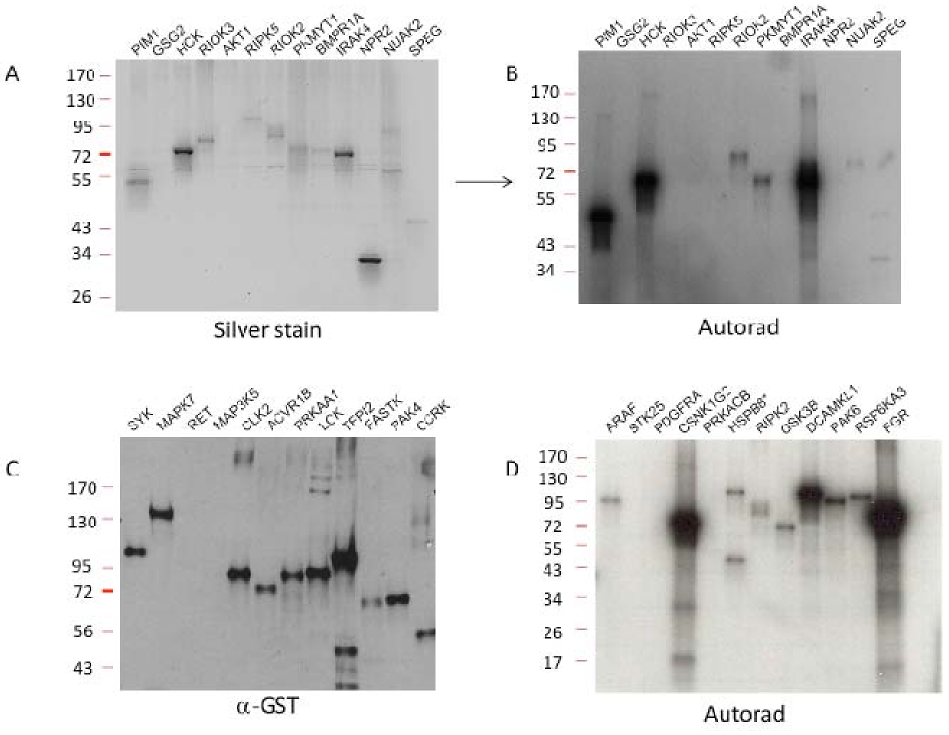

Supplementary Figure 2. **Determination of kinase purity**. GST-kinase fusion proteins were purified by glutathione-affinity chromotography and their purity assessed by silver stain (A), autoradiography (B & D) and western blotting. The proteins shown in (A) and (B) are from the same preparation while those shown in (C) and (D) were from separate preparations. In (D) an asterisk indicates that HSPB8 is known to form disulfide dimers that are resistant to reducing agent.

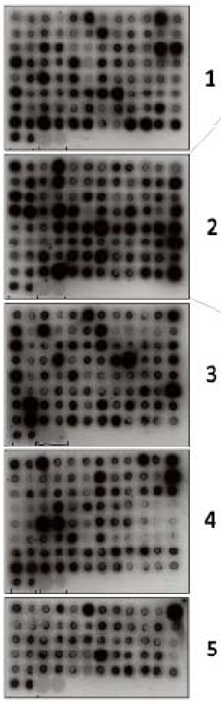

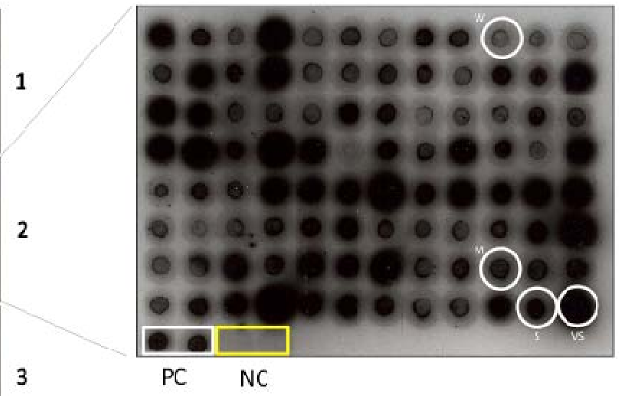

Kinase Activity Summary

Plate	1	2	3	4	5	Total
None	0	0	0	1	0	1
W	16	10	21	15	16	78
M	43	34	36	34	33	180
S	13	21	21	15	8	78
VS	24	31	18	16	3	92
Total	96	96	96	81	60	429

W, weak; M, moderate; S, strong; VS, very strong

Supplementary Figure 3. **Assessment of the apparent enzymatic activity of purified kinases**. Dot blots demonstrating the apparent activity of each purified kinase. Apparent activities were assigned according to the intensity of the spot versus that of a positive control (PC, i.e., commercially-available kinase). Each kinase was designated as either very strong (VS, intensity greater than that of PC), strong (S, intensity similar to that of PC), moderate (M, intensity slightly less than that of PC) or weak (W, intensity substantially less than that of PC). The results are tabulated according to plate number (inset). Overall, 350/429 kinases (~82 %) exhibited an apparent activity ≥ M.

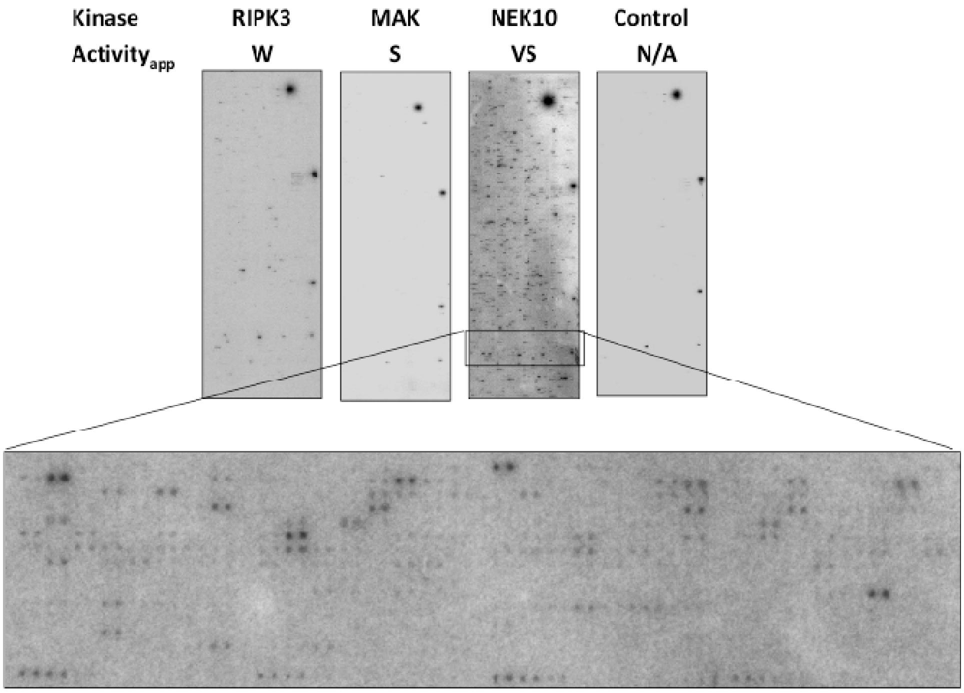

Supplementary Figure 4. **Human protein kinase trans-phosphorylation assay using protein microarrays**. Autoradiograms of individual microarrays treated with the indicated human kinases or a negative control lacking kinase. The apparent activity of each kinase (activity$_{app}$) is shown below each kinase. Notice that a higher activity$_{app}$ does not necessarily correlate with more substrates on the microarray due to the specificity of each kinase (compare RIPK3 vs. MAK).

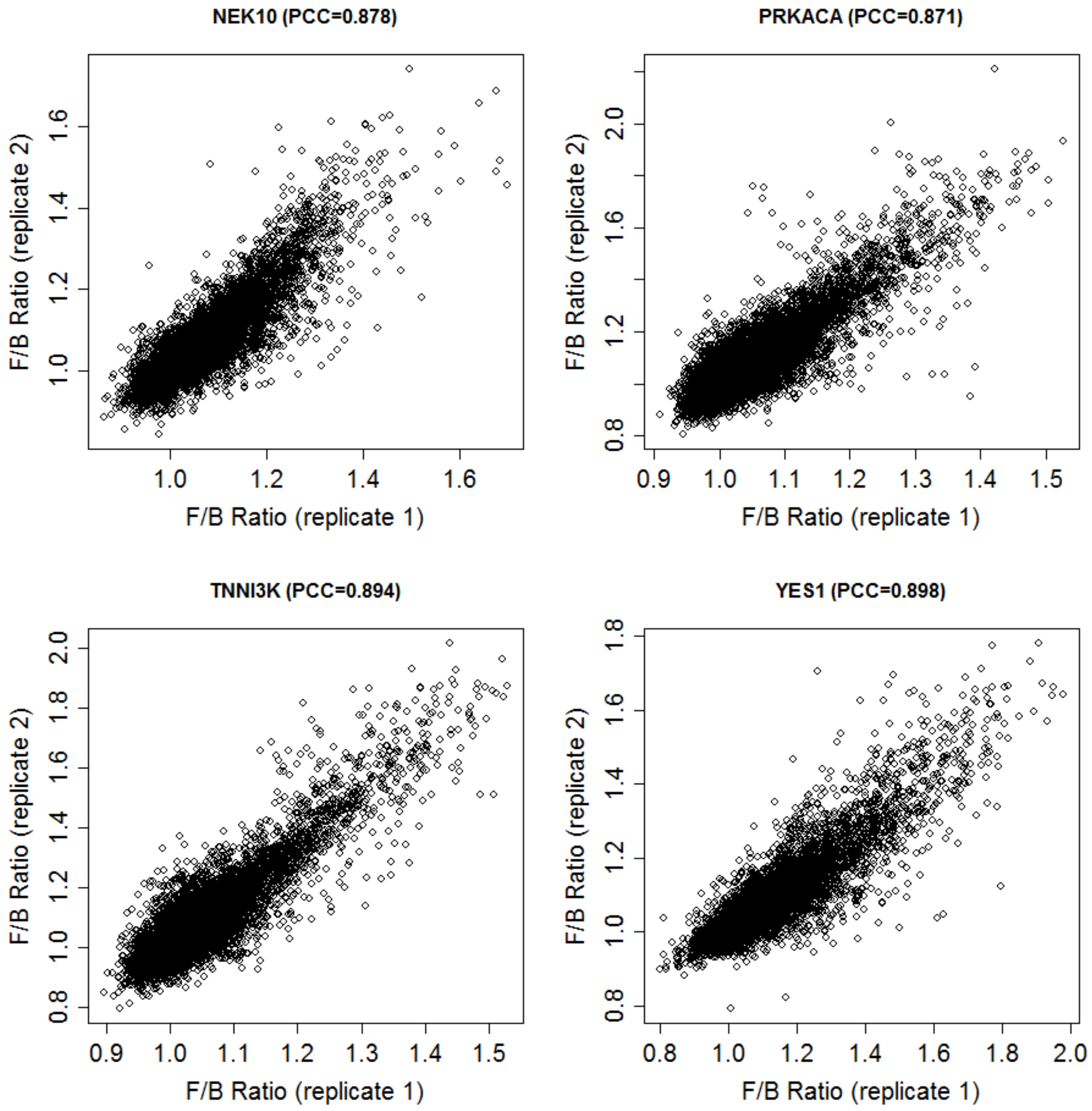

Supplementary Figure 5. Reproducibility of protein microarray. Four duplicate phosphorylation reactions were performed using the indicated kinases. The Pearson Correlation Coefficient (PCC) of signal intensity between duplicates ranged from 0.871 to 0.894. The overlap of positive hits between duplicates ranged from 66% to 72%. F/B Ratio represents the ratio of median signal intensity of foreground and background.

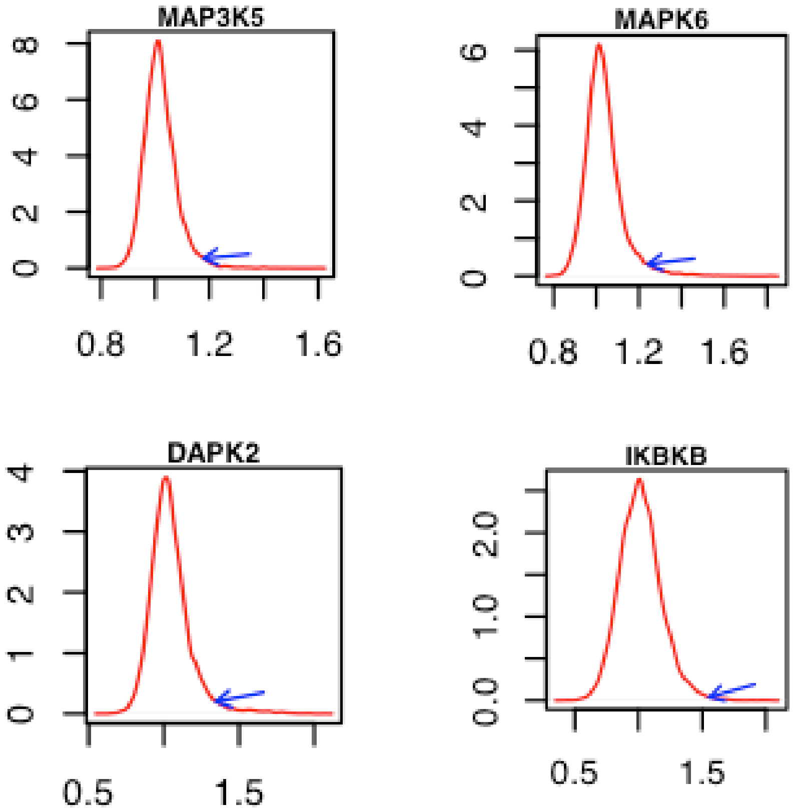

Supplementary Figure 6. **Signal distribution for 4 example kinases**. The signal is defined as the ratio between foreground and background intensities. Signals for most of the spots on the array are close to 1, indicating that the majority of the proteins are not phosphorylated by the kinase-of-interest. Only the spots with signals greater than 3 standard deviations above the mean (indicated by blue arrows) were considered as potential substrates of the kinase.

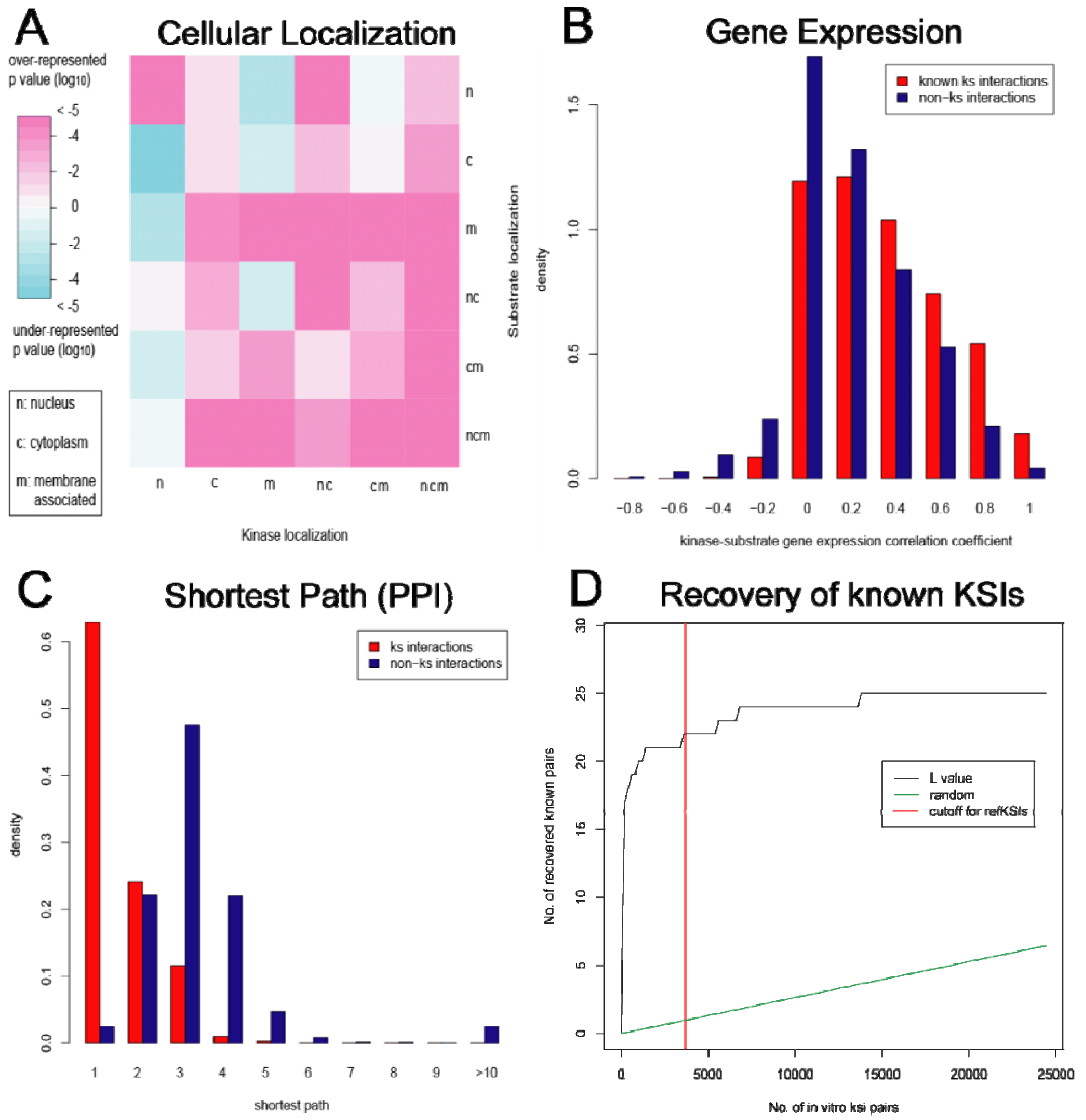

Supplementary Figure 7. **Bayesian analysis of KSR interactions**. (A) Cellular localization. Heat map representing the subcellular localization of kinases (X-axis) and substrates (Y-axis). Those regions which are over-represented are shown as warmer colors while regions that are under-represented are depicted as cooler colors. Different subcellular regions are abbreviated as follows: nucleus (n), cytoplasm (c), membrane (m). (B) Co-expression across tissues. The X-axis shows the correlation coefficient of gene expression profiles between two genes while the Y-axis depicts the density of KSR pairs exhibiting the respective correlation coefficient. (C) Protein-protein interaction; X-axis is

the shortest path length between two proteins in a PPI network. (D) Recovery of known KSRs. The X-axis is the number of KSRs while the Y-axis represents the number of known KSRs in the dataset. The green line is for a random KSR set. The vertical red line indicates the cutoff value for refKSRs.

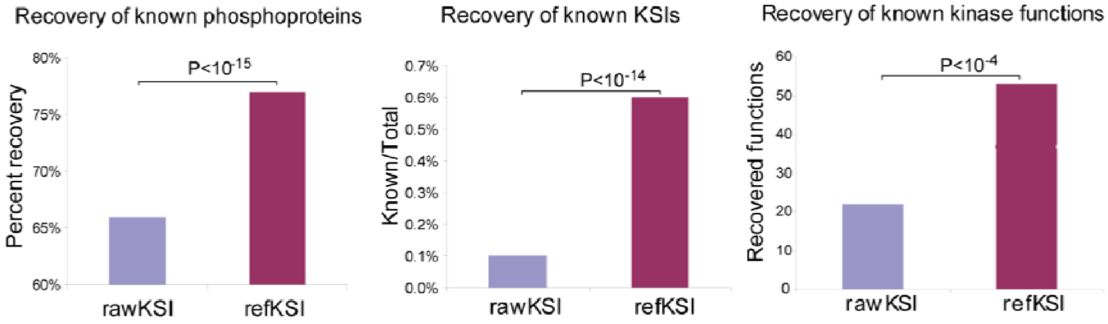

Supplementary Figure 8. **Computational validation of Bayesian analysis**. By comparing the phosphoproteins identified by MS/MS, we calculated the percentage of phosphoproteins that were identified as substrates in the rawKSR and refKSR datasets, respectively (left panel). The number of known KSRs in the rawKSR and refKSR are 25 and 22, respectively. However, because the sizes of the rawKSR and refKSR sets are quite different (24,046 versus 3,654), the recovery rate was improved significantly in the refKSR dataset (middle panel). We also predicted functions of kinases based on the enriched biological functions (GO terms) of their substrates. Using the rawKSR set, we recovered known functions for 22 kinases. In contrast, we correctly predicted the functions for 53 kinases using the refKSRs (right panel).

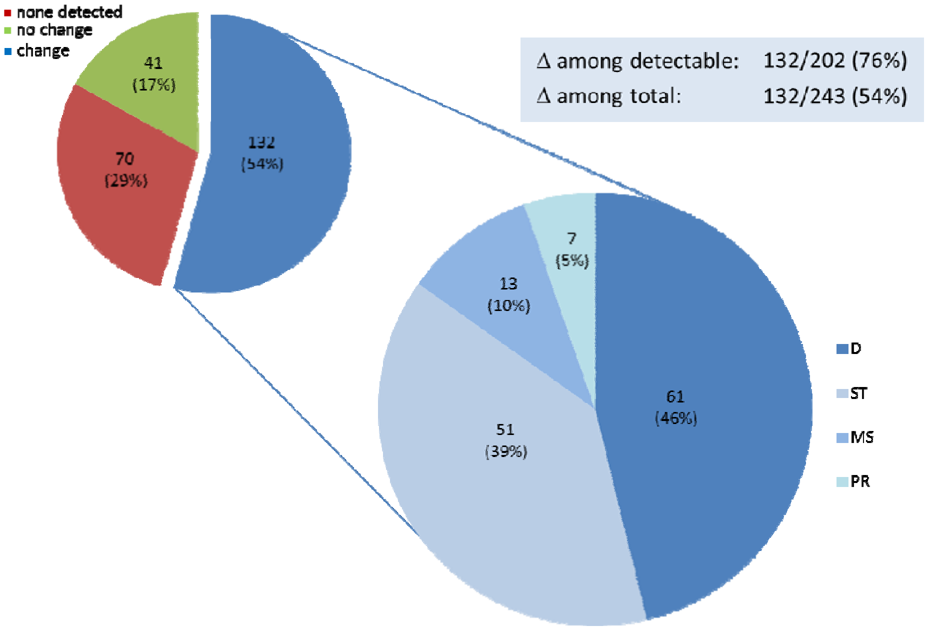

Supplementary Figure 9. **Summary of results from the first-stage validation studies**. The chart in the upper left illustrates the number of substrates, which 1) showed no expression in either the substrate or the substrate plus kinase samples (red), 2) showed no change in their electrophoretic mobility or protein levels in the presence of co-expressed kinase (green), or 3) exhibited kinase-dependent changes in their electrophoretic mobility and/or protein levels (blue). The number of KSRs found in each category is indicated as well as the percent among the total (parenthesis). The chart in the lower right depicts the distribution of specific kinase-dependent changes observed in the assay. ST, stabilization (i.e., increase in substrate levels); D, degradation (i.e., decrease in substrate levels); MS, mobility shift; and PR, processing. Inset: kinase-dependent changes observed among the entire set (among total) and among those substrates that could be detected in the assay (among detectable).

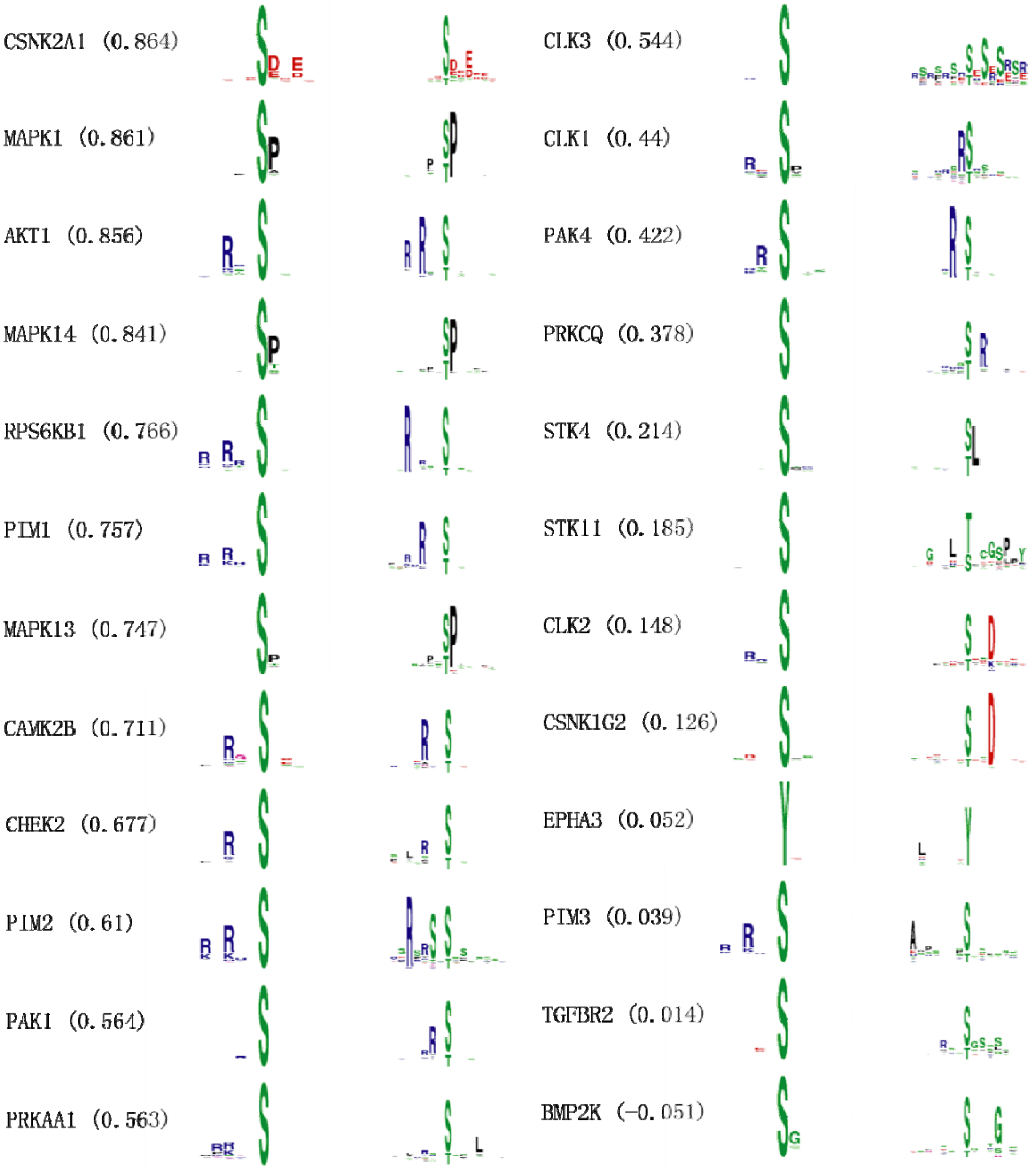

Supplementary Figure 10. Validation of motif predictions. Comparison between the motifs predicted by the M3 algorithm (right) and those determined by the scanning peptide library approach (left). The numbers in the parenthesis represent the similarity scores (i.e., correlation coefficients). The motifs are ranked according to their similarity scores.

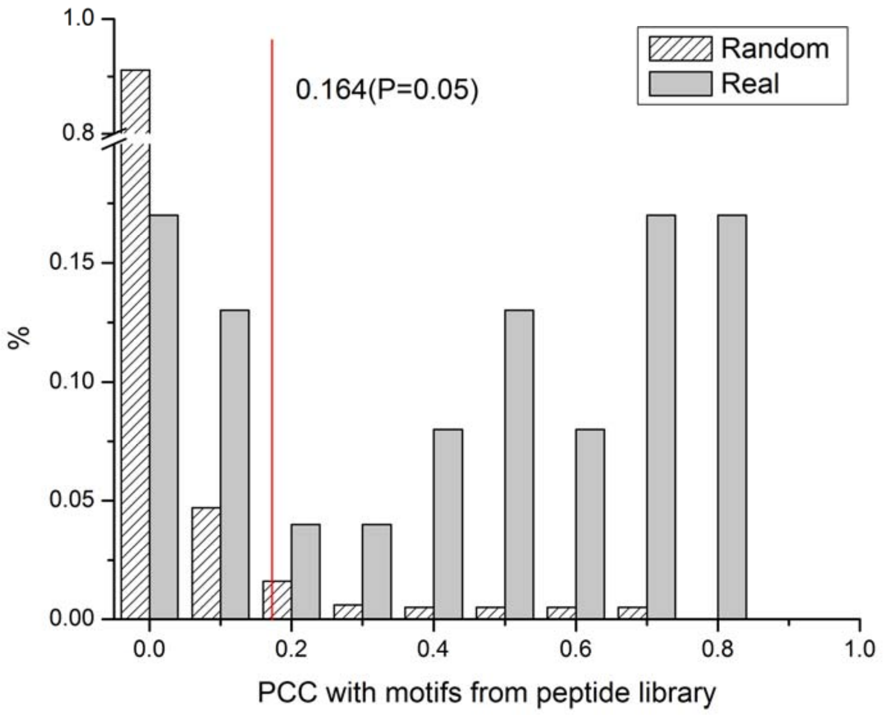

Supplementary Figure 11. **Distribution of the similarity scores between the M3 motifs and those determined using the scanning peptide library approach.** We performed a random simulation by shuffling the position weight matrices (PWMs) and then calculated the similarity scores between the randomized motifs. The red line indicates the cutoff value (0.164) corresponding to a *P* value of 0.05. This cutoff value was used as a criterion to determine the number of M3 motifs with significant similarity to those identified by a scanning peptide array library.

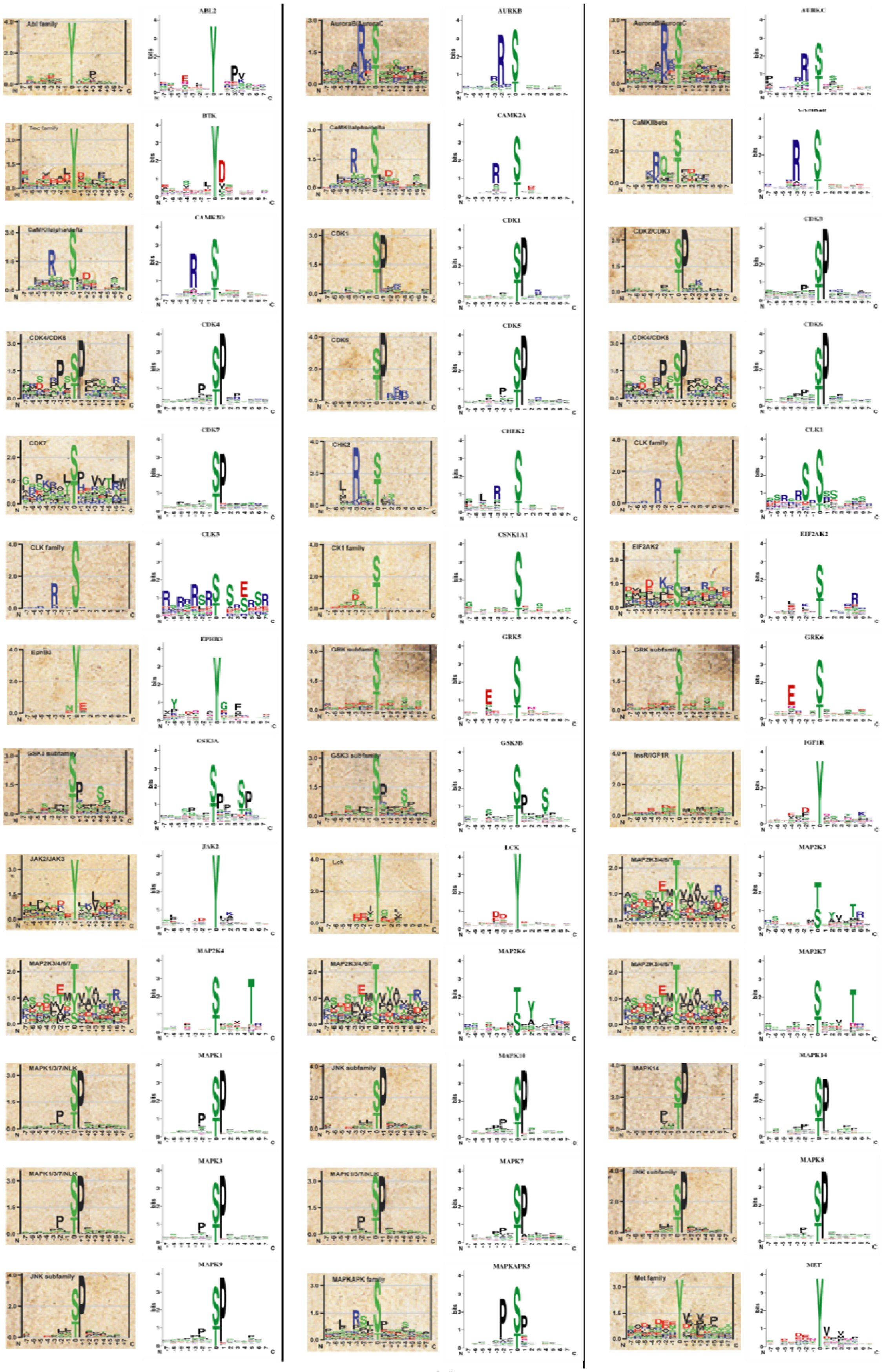
Abl family
ABL2
AURKB
AURKC
BTK
CAMK2A
CAMK2D
CDK1
CDK3
CDK4
CDK5
CDK6
CDK7
CHEK2
CLK1
CLK3
CSNK1A1
EIF2AK2
EPHB3
GRK5
GRK6
GSK3A
GSK3B
IGF1R
JAK2
LCK
MAP2K3
MAP2K4
MAP2K6
MAP2K7
MAPK1
MAPK10
MAPK14
MAPK3
MAPK7
MAPK8
MAPK9
MAPKAPK5
MET

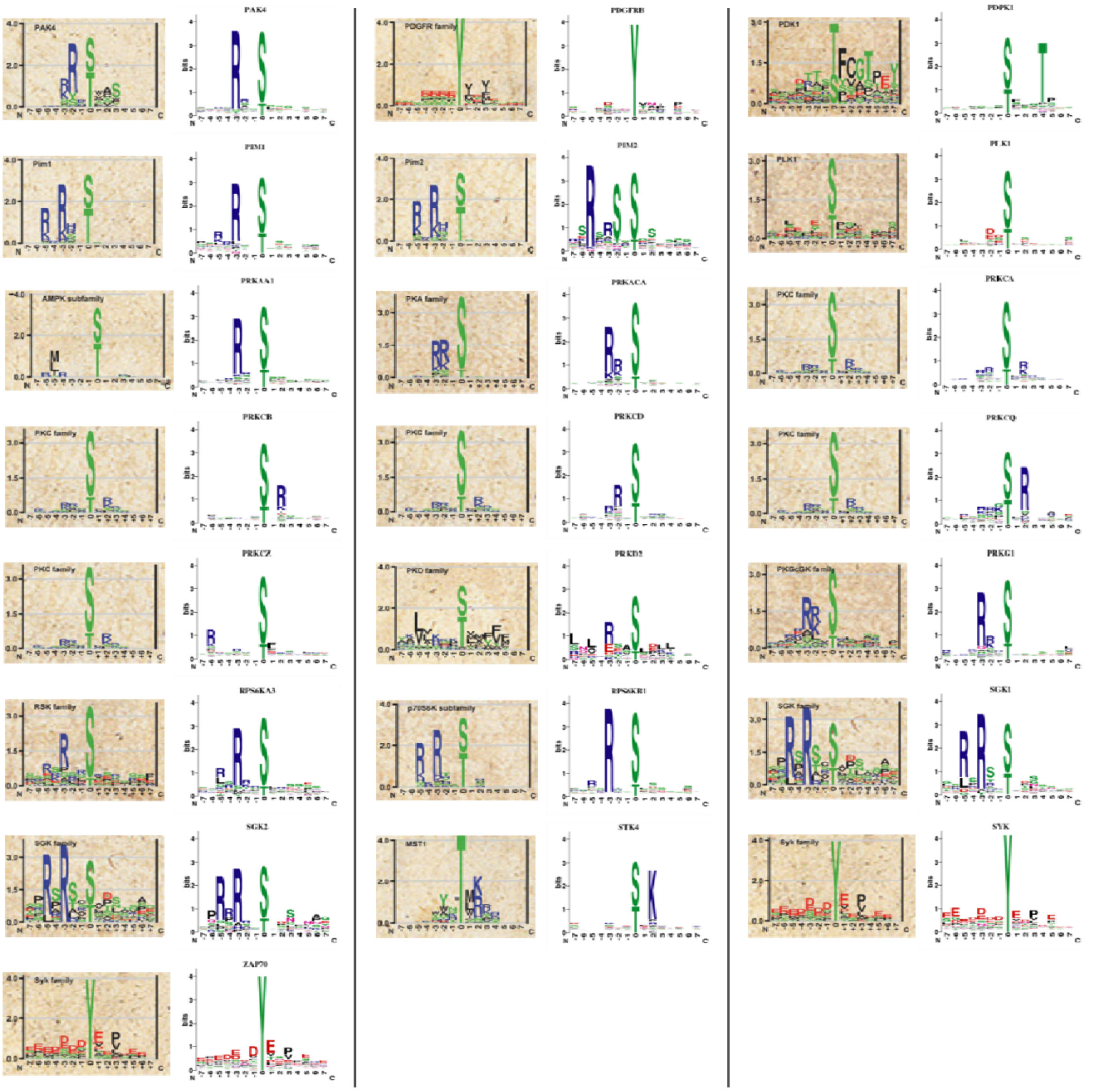

Supplementary Figure 12. Recovery of motifs predicted in the literature. M3 motifs (right) were compared with those predicted by Miller et al. (left). Please note that the similarity comparison between these motifs was based on visual inspection because the PWMs of the published motifs are not available.

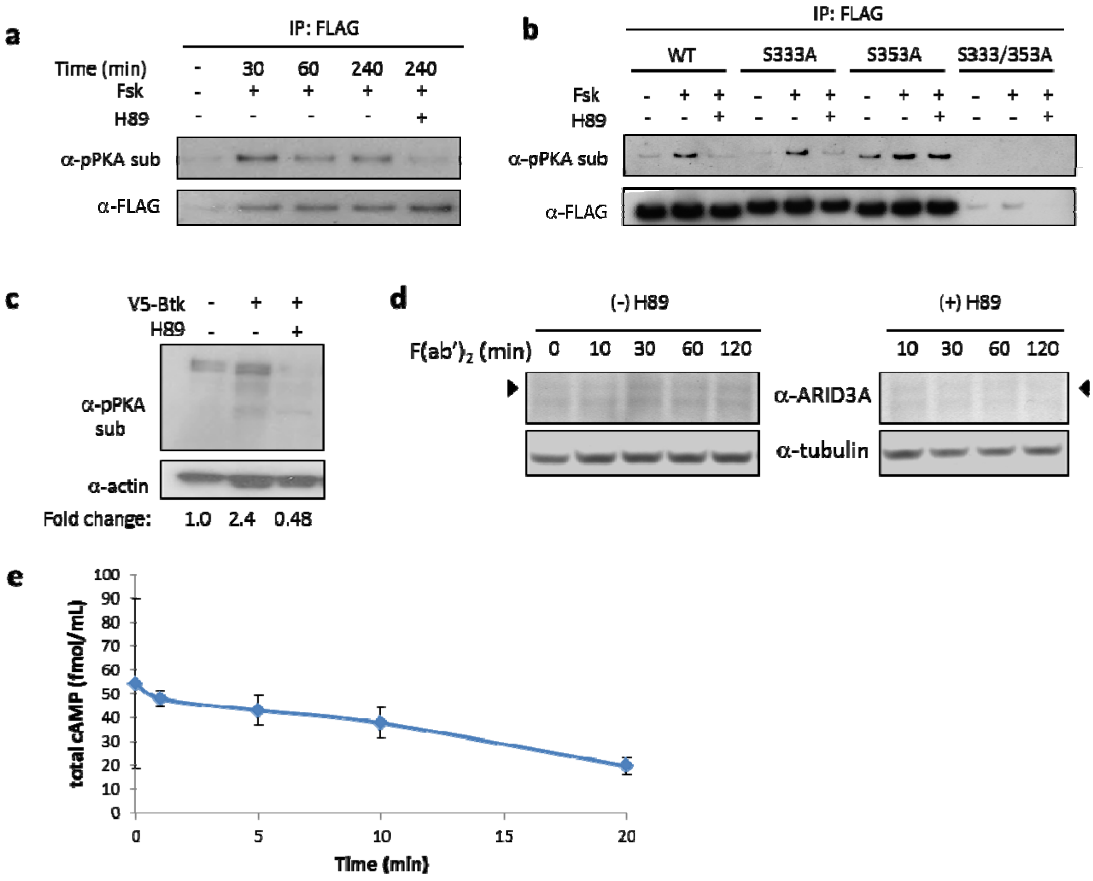

Supplementary Figure 13. Btk-PKA-ARID3A validation. **a)** FLAG-tagged ARID3A was expressed in HeLa cells for 20 hours. Endogenous PKA was then activated for the indicated times with 50 mM forskolin (Fsk) in the presence or absence of the PKA inhibitor, H89. FLAG-ARID3A was then immunoprecipitated and the extent of PKA-mediated phosphorylation assessed by an anti-p(S/T) PKA substrate antibody (α-pPKA sub) (Cell Signaling Technologies). Blots were then stripped and reprobed with an anti-FLAG antibody (α-FLAG). **b)** The indicated ARID3A variant was expressed in HeLa cells and the extent of PKA-mediated phosphorylation measured as described in (a). During these experiments, endogenous PKA was stimulated with 50 mM Fsk for 30 minutes in the presence or absence of H89. **c)** V5-tagged Btk was expressed in the presence or absence of H89 in HeLa cells and the extent of PKA-mediated

phosphorylation in the lysate was assessed using the anti-p(S/T) PKA substrate antibody used in (a). An anti-actin antibody (α-actin) was used to ensure that similar amount of protein was loaded into each lane. The normalized signal intensity (pPKA substrate/actin) of the entire lane is shown. **d)** Endogenous ARID3A was monitored in Ramos B cell lysates following a time course of $F(ab')_2$ treatment in the presence or absence of H89. ARID3A levels were normalized to a non-specific band recognized by the primary antibody. **e)** Global cAMP levels, expressed in fmol/mL assay buffer, were measured at various times after $F(ab')_2$ addition in Ramos B cells using a commercially-available cAMP EIA kit (Sigma). In the time course shown, cells were at a density of $\sim 10^6$ cells/mL. Similar results were also obtained at cell densities of 10^4 and 10^5 cells/mL. Each point is the average of 3 independent experiments. Error bars represent the standard deviation at each time point. No $F(ab')_2$ was added at time 0.

4. SUPPLEMENTARY TABLES

Supplementary Table 1. List of 289 unique kinases analyzed in this study.

AAK1	ABL2	ACVR1	ACVR1B	ACVR2A	ACVR2B	ACVRL1	ADCK1
ADCK3	ADCK5	ADRBK1	ADRBK2	AKT1	AKT3	ARAF	AURKB
AURKC	AXL	BLK	BMP2K	BMPR1A	BMPR1B	BMPR2	BMX
BTK	BUB1	CAMK1D	CAMK2A	CAMK2B	CAMK2D	CAMK2G	CAMK4
CAMKK1	CAMKK2	CAMKV	CDK1	CDK10	CDK13	CDK14	CDK15
CDK16	CDK17	CDK18	CDK19	CDK3	CDK4	CDK5	CDK6
CDK7	CDK9	CDKL3	CDKL5	CHEK2	CLK1	CLK2	CLK3
CLK4	CSK	CSNK1A1	CSNK1A1L	CSNK1D	CSNK1E	CSNK1G1	CSNK1G2
CSNK1G3	CSNK2A1	CSNK2A2	DCLK1	DCLK2	DDR1	DDR2	DMPK
DSTYK	DYRK1B	DYRK2	DYRK3	DYRK4	EEF2K	EIF2AK1	EIF2AK2
EPHA3	EPHB1	EPHB3	EPHB4	FASTK	FGFR1	FGFR2	FGFR4
FGR	FRK	FYN	GRK5	GRK6	GSG2	GSK3A	GSK3B
HCK	HIPK1	HIPK4	ICK	IGF1R	IKBKB	ILK	IRAK1
IRAK3	ITK	JAK2	KIT	KSR2	LATS1	LCK	LIMK2
LYN	MAK	MAP2K1	MAP2K2	MAP2K3	MAP2K4	MAP2K5	MAP2K6
MAP2K7	MAP3K11	MAP3K13	MAP3K14	MAP3K7	MAP3K8	MAP4K2	MAP4K5
MAPK1	MAPK10	MAPK11	MAPK12	MAPK13	MAPK14	MAPK15	MAPK3
MAPK6	MAPK7	MAPK8	MAPK9	MAPKAPK3	MAPKAPK5	MARK2	MARK3
MAST1	MAST2	MATK	MET	MKNK1	MKNK2	MLKL	MOS
MST4	MYLK	MYLK2	MYO3A	NEK10	NEK11	NEK2	NEK3
NEK4	NEK6	NEK7	NEK8	NEK9	NIM1	NLK	NPR2
NRBP1	NRBP2	NTRK3	NUAK1	NUAK2	OXSR1	PAK1	PAK4
PAK6	PBK	PDGFRB	PDIK1L	PDK3	PDK4	PDPK1	PHKG2
PIK3C3	PIK3R4	PIM1	PIM2	PIM3	PINK1	PKMYT1	PKN1
PKN3	PLK1	PLK2	PLK3	PNCK	PRKAA1	PRKAA2	PRKACA
PRKACB	PRKACG	PRKCA	PRKCB	PRKCD	PRKCH	PRKCI	PRKCQ
PRKCZ	PRKD2	PRKG1	PRKX	PRKY	PRPF4B	PTK2	PTK2B
PTK6	PXK	RAF1	RAGE	RET	RIOK1	RIPK2	RIPK3
RPS6KA1	RPS6KA2	RPS6KA3	RPS6KA4	RPS6KA5	RPS6KA6	RPS6KB1	RPS6KB2
RPS6KL1	SCYL1	SCYL2	SCYL3	SGK1	SGK196	SGK2	SGK3
SGK494	SIK1	SIK2	SNRK	SRPK1	SRPK2	STK11	STK16
STK17A	STK24	STK25	STK3	STK31	STK32A	STK32B	STK33
STK36	STK38	STK38L	STK4	STK40	STYK1	SYK	TBCK
TBK1	TESK2	TGFBR2	TNK2	TNNI3K	TRIB2	TRIB3	TSSK1B
TSSK2	TSSK3	TTBK2	TTK	TYK2	TYRO3	UHMK1	ULK3
ULK4	VRK1	VRK2	VRK3	WEE1	WNK1	YES1	YSK4
ZAP70							

Supplementary Table 2. List of rawKSRs.

"rawKSR.xls" contains all original raw kinase substrate relationships (KSRs) with signal intensities greater than a certain cutoff (equivalent to 3 standard deviations above the mean for each microarray). This file includes 24,046 KSRs involving 289 unique kinases and 1,967 unique substrates.

Supplementary Table 3. List of refKSRs.

"refKSR.xls" contains kinase substrate pairs that are predicted to be physiologically relevant using the Bayesian method. This file includes 3,656 refined KSRs involving 255 unique kinases and 742 substrates.

Supplementary Table 4. Summary of the first-stage KSR validation results.

Class	No.	%
D (Degradation)	61	25.1%
ST (Stabilization)	51	21.0%
MS (Mobility Shift)	13	5.3%
PR (Processing)	7	2.9%
NC (No Change)	41	16.9%
NE (No Expression)	70	28.8%
Total	243	100%

Supplementary Table 5. Summary of the second-stage validation results. D, degradation; ST, stabilization; MS, mobility shift; ND, not determined; Y, yes; N, no.

Substrate	Kinase	Results in 1st round	Antibody	Confirmed?
HCLS1	PKC	D	p(S) PKC sub	Y
MAGEB6	PKC	D	p(S) PKC sub	Y
ANKS1A	PKC	D	p(S) PKC sub	N
DDEFL1	PKC	ST	p(S) PKC sub	Y
PRKAR1A	PKA	ST	p(S/T) PKA sub	Y
HLCS	PKA	D	p(S/T) PKA sub	Y
DAXX	PKA	ST	p(S/T) PKA sub	Y
NFATC3	PKA	ST	p(S/T) PKA sub	Y
TRIP10	PKA	ST	p(S/T) PKA sub	Y
NEUROD1	PKA	ST	p(S/T) PKA sub	N
HCLS1	PKA	MS	p(S/T) PKA sub	Y
ARID3A	PKA	ST	p(S/T) PKA sub	Y
PKCD	AKT	D	p(S/T) AKT sub	Y
ZRANB2	AKT	D	p(S/T) AKT sub	Y
TRIP10	AKT	D	p(S/T) AKT sub	Y
SFRS1	AKT	D/MS	p(S/T) AKT sub	Y
LUC7L2	AKT	D	p(S/T) AKT sub	N
SMAD3	AKT	D	p(S/T) AKT sub	N
PPP1R13L	MAPK	ST	p(S/T)-Pro	Y
**Summary**				**15/19**
**Confirmation rate**				**79%**

Supplementary Table 6. List of comKSRs.

"comKSR.xls" contains the 3,656 refKSRs listed in Table S3 as well as 741 known KSRs curated from the literature.

5. SUPPLEMENTARY FILES

5.1 Logos of 300 predicted phosphorylation motifs.

"logo.rar" file contains 300 motif logos for 284 human kinases predicted by our M3 algorithm, representing 55% of the human kinome. In the case of dual-specificity kinases, we separately considered motifs that contained pS/T or pY sites.

5.2 Phosphorylation network.

A computational algorithm was developed to connect the *in vivo* phosphorylation sites to their putative upstream kinases. For a given kinase, the *in vivo* phosphorylation sites within its substrates in the comKSR dataset were screened for those sites that closely matched its phosphorylation motif. This analysis resulted in a high-resolution phosphorylation map that allows phosphorylation events to be identified at the amino acid level.

The "cytoscape.cys" session file includes the final high-resolution phosphorylation map connecting 230 kinases to 2,591 in vivo phosphorylation sites in 652 substrates, representing 4,417 kinase-to-phosphorylation site relationships.

To facilitate the usage of this network, we built an interactive website at http://phosphonetworks.org, which allows the user easily to browse and search kinases- and substrates-of-interest.

5.3 Validation files

"Validation.rar" is a compressed file (~ 1 GB), which contains all the raw experimental validation evidence (gel images) that we produced in this project. It includes one summary excel file and four sub-directories, "1st stage validation", "2nd stage validation", "high resolution map validation", and "missing link validation".
