# Supplementary figures and images for: Construction of human activity-based phosphorylation networks (part 2 of 2)
Source: Mol Syst Biol. 2013 Apr 2;9:655. doi: 10.1038/msb.2013.12 (PMC3658267; doi:10.1038/msb.2013.12)

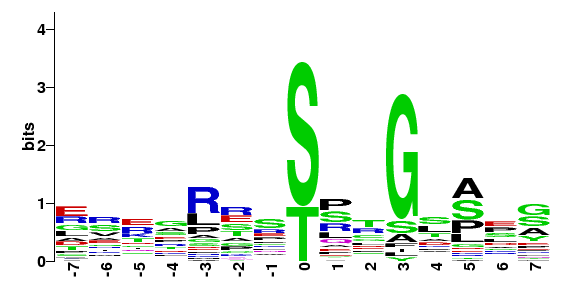

Supplement: Logos of 300 phosphorylation motifs predicted [file msb201312-s6.zip › Logo/HIPK1.png]

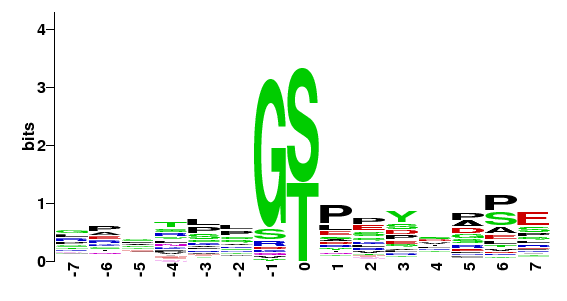

Supplement: Logos of 300 phosphorylation motifs predicted [file msb201312-s6.zip › Logo/HIPK4.png]

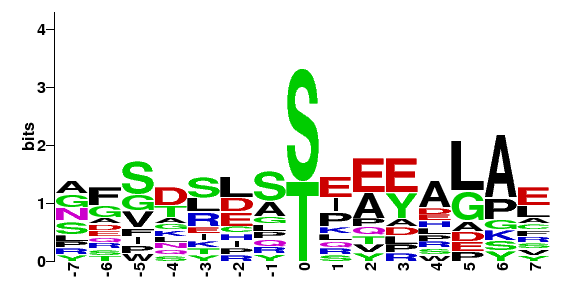

Supplement: Logos of 300 phosphorylation motifs predicted [file msb201312-s6.zip › Logo/ICK.png]

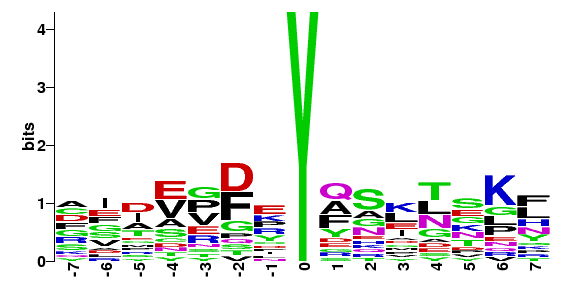

Supplement: Logos of 300 phosphorylation motifs predicted [file msb201312-s6.zip › Logo/IGF1R.png]

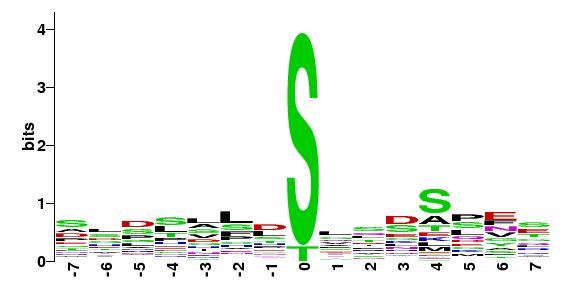

Supplement: Logos of 300 phosphorylation motifs predicted [file msb201312-s6.zip › Logo/IKBKB.png]

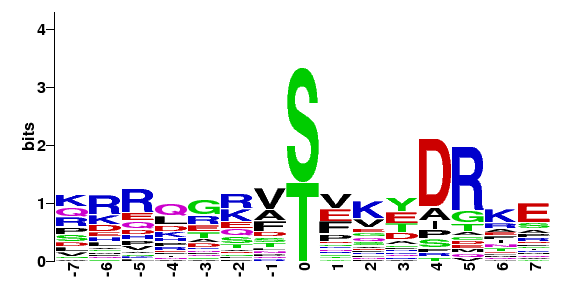

Supplement: Logos of 300 phosphorylation motifs predicted [file msb201312-s6.zip › Logo/ILK.png]

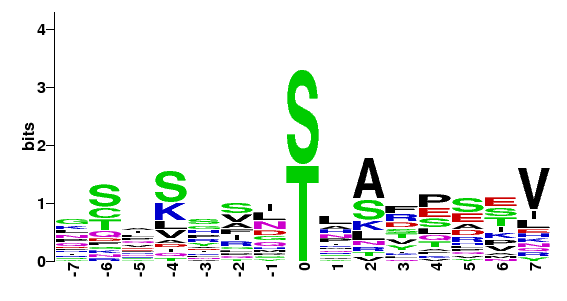

Supplement: Logos of 300 phosphorylation motifs predicted [file msb201312-s6.zip › Logo/IRAK1.png]

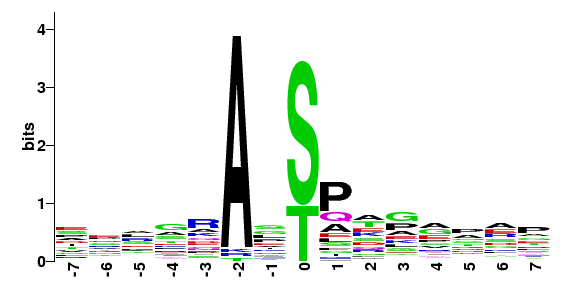

Supplement: Logos of 300 phosphorylation motifs predicted [file msb201312-s6.zip › Logo/IRAK3.png]

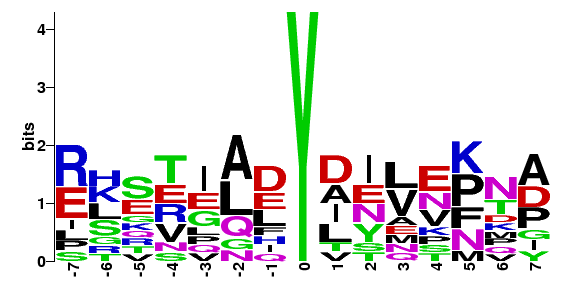

Supplement: Logos of 300 phosphorylation motifs predicted [file msb201312-s6.zip › Logo/ITK.png]

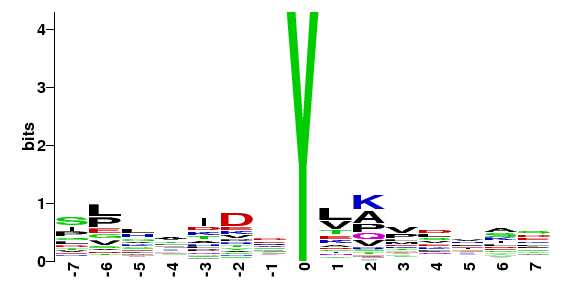

Supplement: Logos of 300 phosphorylation motifs predicted [file msb201312-s6.zip › Logo/JAK2.png]

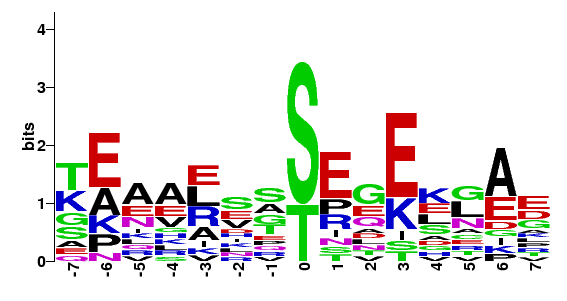

Supplement: Logos of 300 phosphorylation motifs predicted [file msb201312-s6.zip › Logo/KSR2.png]

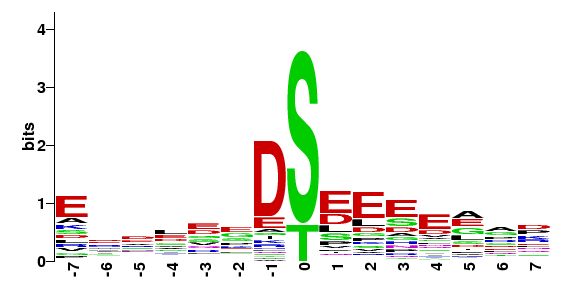

Supplement: Logos of 300 phosphorylation motifs predicted [file msb201312-s6.zip › Logo/LATS1.png]

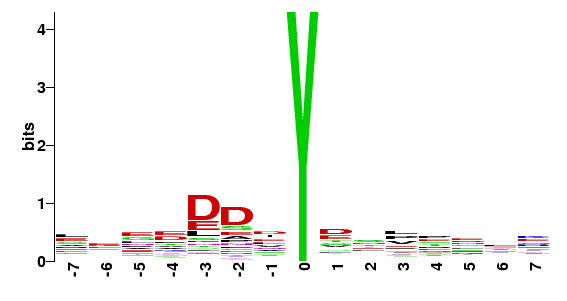

Supplement: Logos of 300 phosphorylation motifs predicted [file msb201312-s6.zip › Logo/LCK.png]

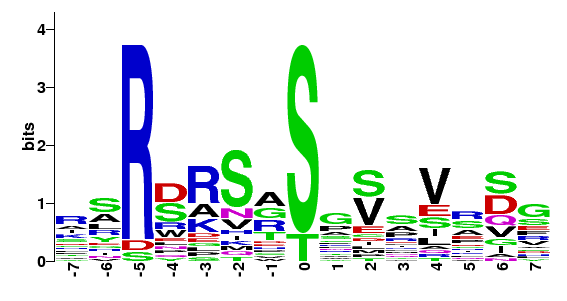

Supplement: Logos of 300 phosphorylation motifs predicted [file msb201312-s6.zip › Logo/LIMK2.png]

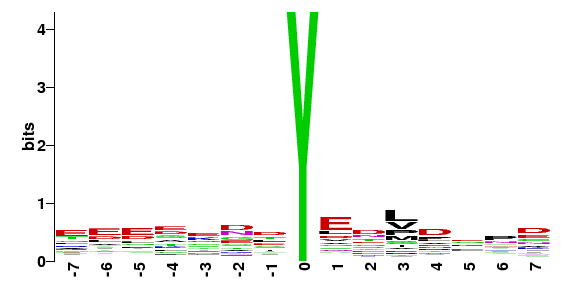

Supplement: Logos of 300 phosphorylation motifs predicted [file msb201312-s6.zip › Logo/LYN.png]

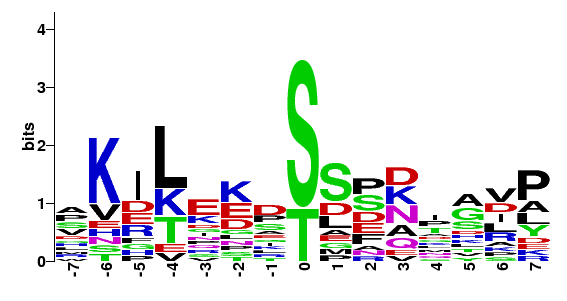

Supplement: Logos of 300 phosphorylation motifs predicted [file msb201312-s6.zip › Logo/MAK.png]

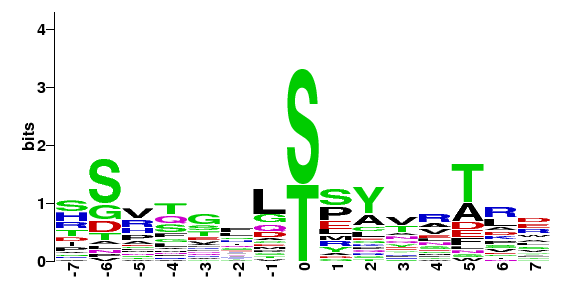

Supplement: Logos of 300 phosphorylation motifs predicted [file msb201312-s6.zip › Logo/MAP2K1.png]

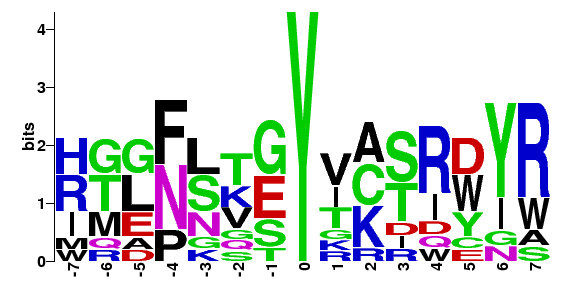

Supplement: Logos of 300 phosphorylation motifs predicted [file msb201312-s6.zip › Logo/MAP2K1_Y.png]

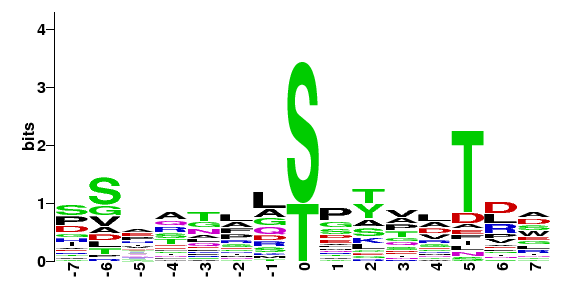

Supplement: Logos of 300 phosphorylation motifs predicted [file msb201312-s6.zip › Logo/MAP2K2.png]

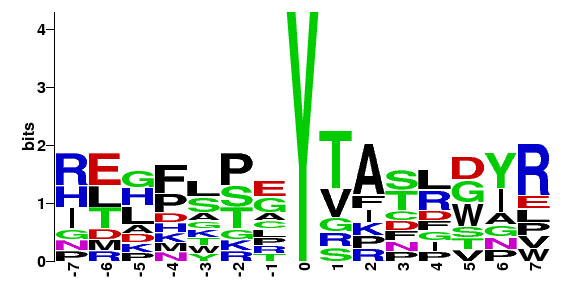

Supplement: Logos of 300 phosphorylation motifs predicted [file msb201312-s6.zip › Logo/MAP2K2_Y.png]

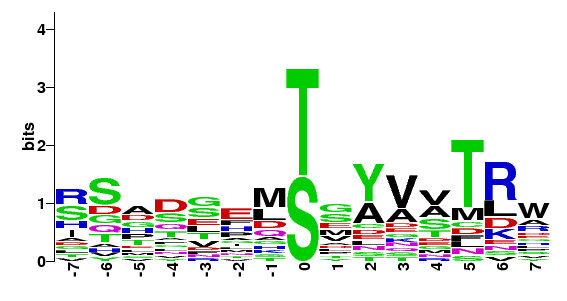

Supplement: Logos of 300 phosphorylation motifs predicted [file msb201312-s6.zip › Logo/MAP2K3.png]

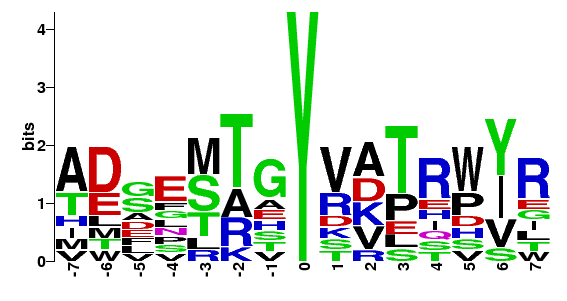

Supplement: Logos of 300 phosphorylation motifs predicted [file msb201312-s6.zip › Logo/MAP2K3_Y.png]

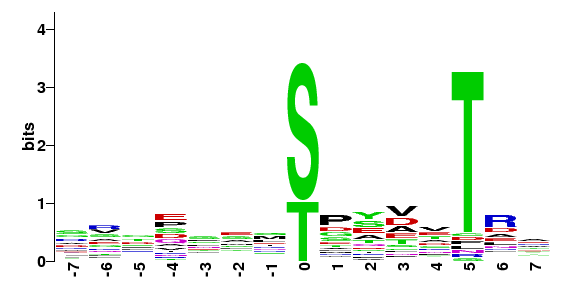

Supplement: Logos of 300 phosphorylation motifs predicted [file msb201312-s6.zip › Logo/MAP2K4.png]

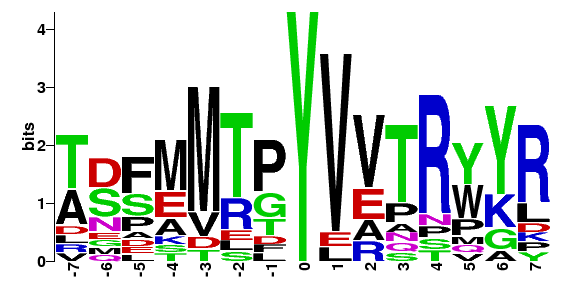

Supplement: Logos of 300 phosphorylation motifs predicted [file msb201312-s6.zip › Logo/MAP2K4_Y.png]

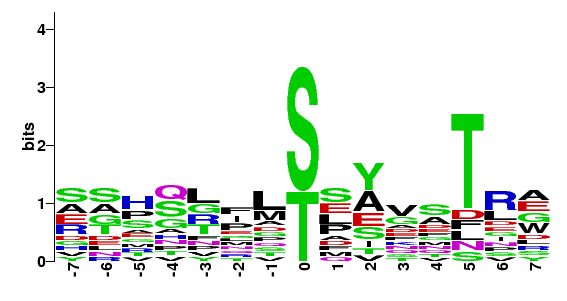

Supplement: Logos of 300 phosphorylation motifs predicted [file msb201312-s6.zip › Logo/MAP2K5.png]

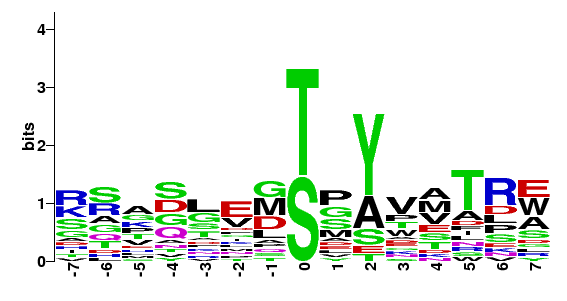

Supplement: Logos of 300 phosphorylation motifs predicted [file msb201312-s6.zip › Logo/MAP2K6.png]

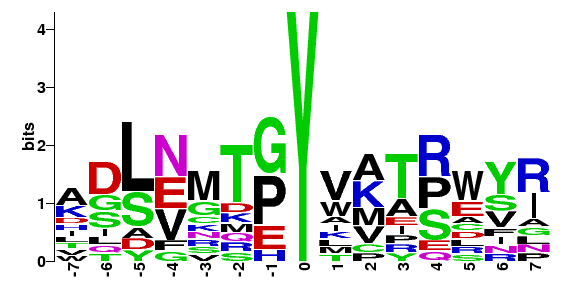

Supplement: Logos of 300 phosphorylation motifs predicted [file msb201312-s6.zip › Logo/MAP2K6_Y.png]

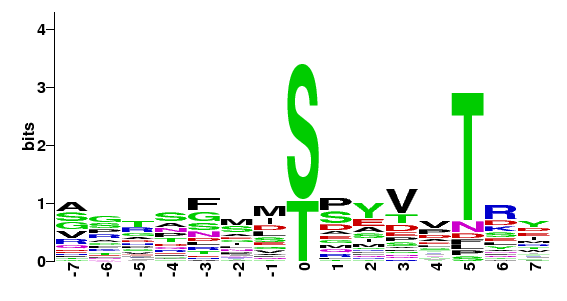

Supplement: Logos of 300 phosphorylation motifs predicted [file msb201312-s6.zip › Logo/MAP2K7.png]

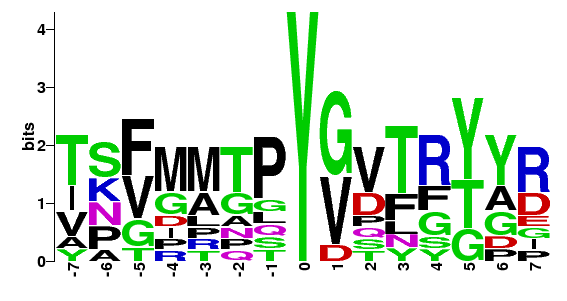

Supplement: Logos of 300 phosphorylation motifs predicted [file msb201312-s6.zip › Logo/MAP2K7_Y.png]

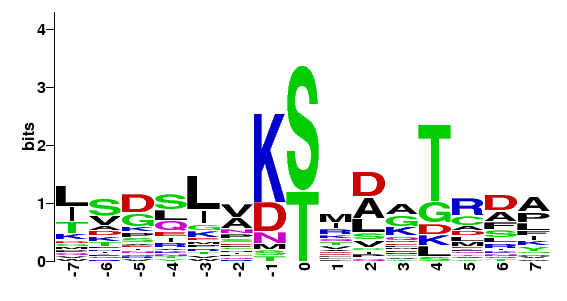

Supplement: Logos of 300 phosphorylation motifs predicted [file msb201312-s6.zip › Logo/MAP3K11.png]

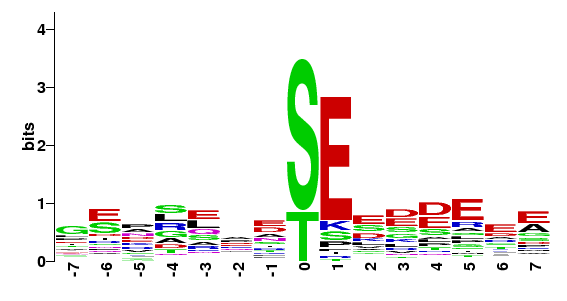

Supplement: Logos of 300 phosphorylation motifs predicted [file msb201312-s6.zip › Logo/MAP3K13.png]

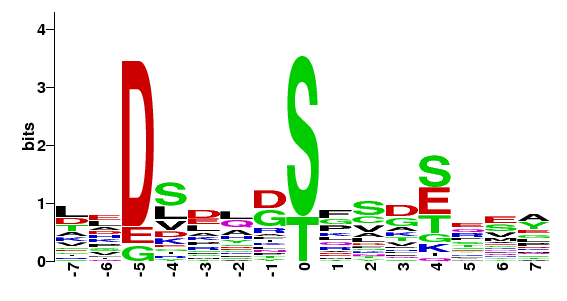

Supplement: Logos of 300 phosphorylation motifs predicted [file msb201312-s6.zip › Logo/MAP3K14.png]

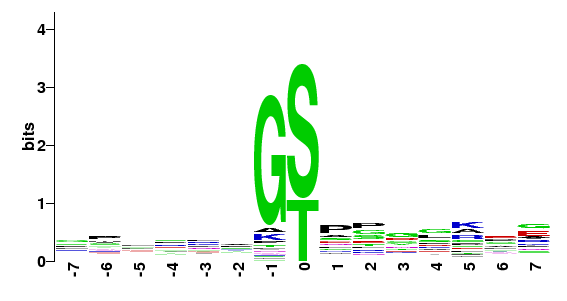

Supplement: Logos of 300 phosphorylation motifs predicted [file msb201312-s6.zip › Logo/MAP3K7.png]

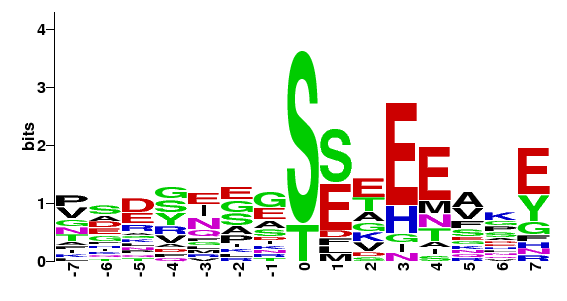

Supplement: Logos of 300 phosphorylation motifs predicted [file msb201312-s6.zip › Logo/MAP3K8.png]

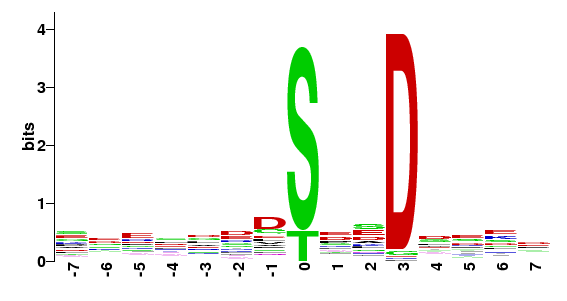

Supplement: Logos of 300 phosphorylation motifs predicted [file msb201312-s6.zip › Logo/MAP4K2.png]

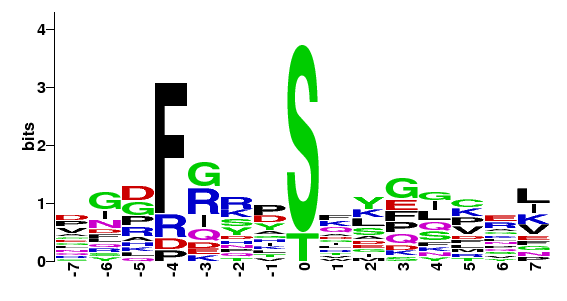

Supplement: Logos of 300 phosphorylation motifs predicted [file msb201312-s6.zip › Logo/MAP4K5.png]

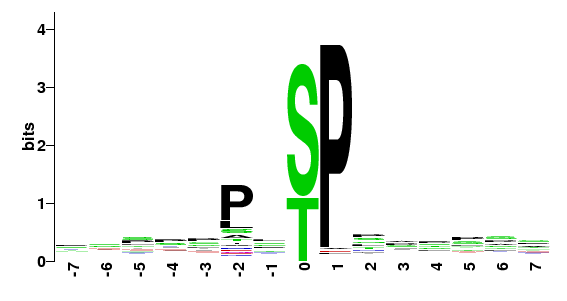

Supplement: Logos of 300 phosphorylation motifs predicted [file msb201312-s6.zip › Logo/MAPK1.png]

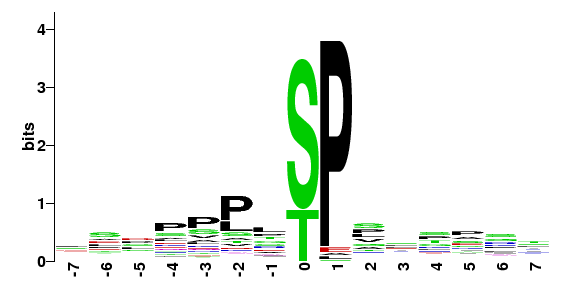

Supplement: Logos of 300 phosphorylation motifs predicted [file msb201312-s6.zip › Logo/MAPK10.png]

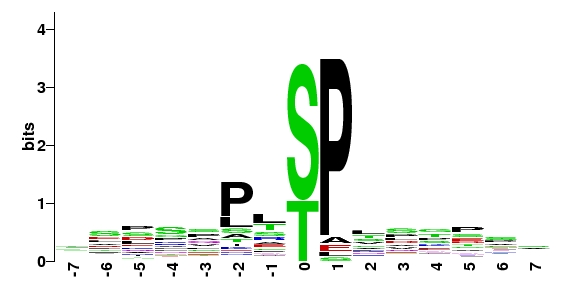

Supplement: Logos of 300 phosphorylation motifs predicted [file msb201312-s6.zip › Logo/MAPK11.png]

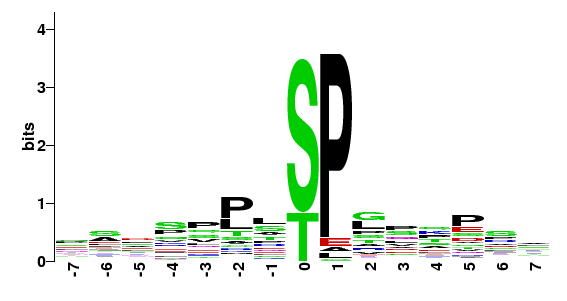

Supplement: Logos of 300 phosphorylation motifs predicted [file msb201312-s6.zip › Logo/MAPK12.png]

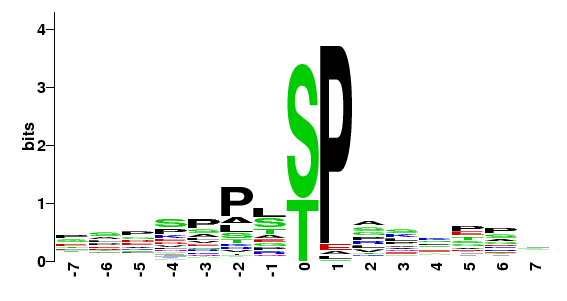

Supplement: Logos of 300 phosphorylation motifs predicted [file msb201312-s6.zip › Logo/MAPK13.png]

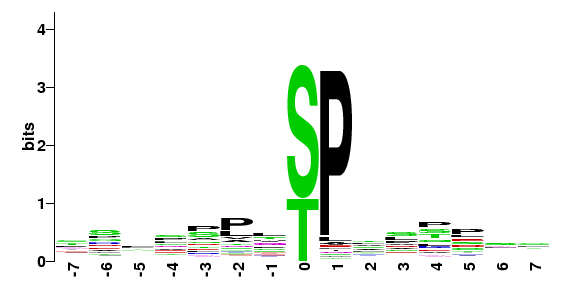

Supplement: Logos of 300 phosphorylation motifs predicted [file msb201312-s6.zip › Logo/MAPK14.png]

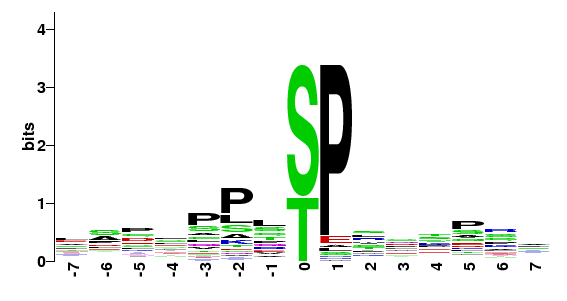

Supplement: Logos of 300 phosphorylation motifs predicted [file msb201312-s6.zip › Logo/MAPK15.png]

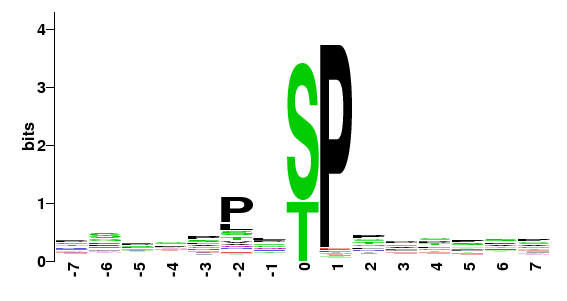

Supplement: Logos of 300 phosphorylation motifs predicted [file msb201312-s6.zip › Logo/MAPK3.png]

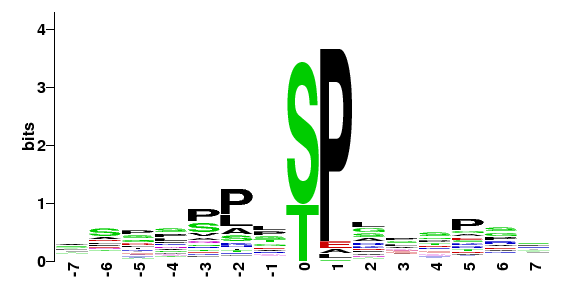

Supplement: Logos of 300 phosphorylation motifs predicted [file msb201312-s6.zip › Logo/MAPK6.png]

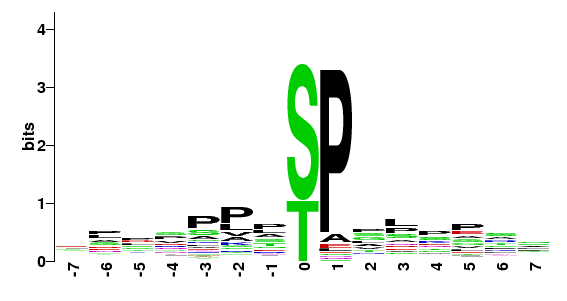

Supplement: Logos of 300 phosphorylation motifs predicted [file msb201312-s6.zip › Logo/MAPK7.png]

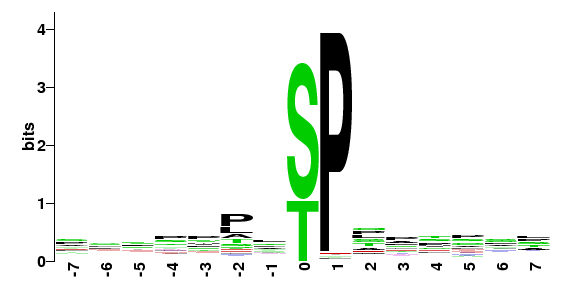

Supplement: Logos of 300 phosphorylation motifs predicted [file msb201312-s6.zip › Logo/MAPK8.png]

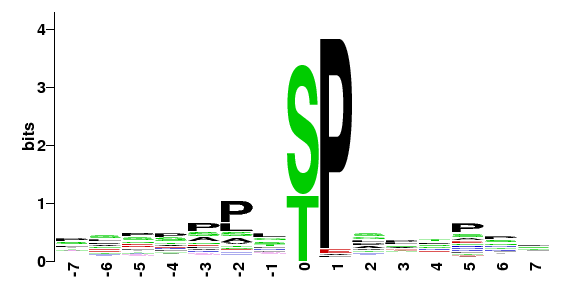

Supplement: Logos of 300 phosphorylation motifs predicted [file msb201312-s6.zip › Logo/MAPK9.png]

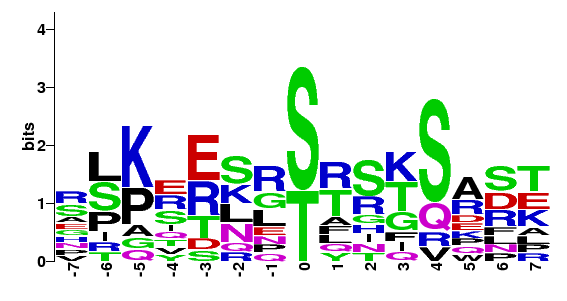

Supplement: Logos of 300 phosphorylation motifs predicted [file msb201312-s6.zip › Logo/MAPKAPK3.png]

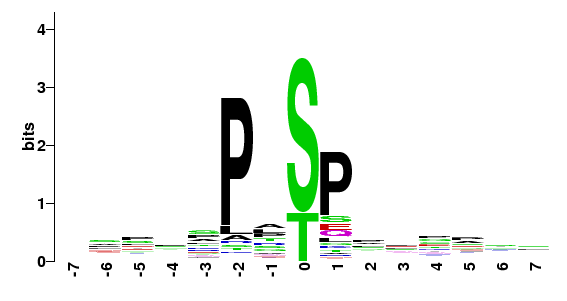

Supplement: Logos of 300 phosphorylation motifs predicted [file msb201312-s6.zip › Logo/MAPKAPK5.png]

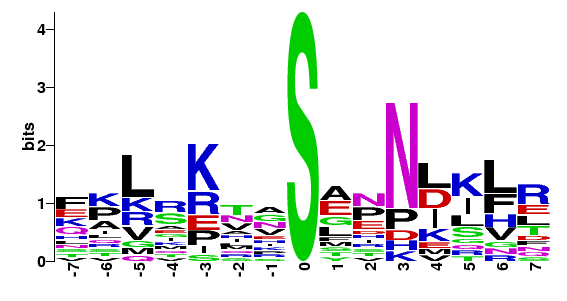

Supplement: Logos of 300 phosphorylation motifs predicted [file msb201312-s6.zip › Logo/MARK2.png]

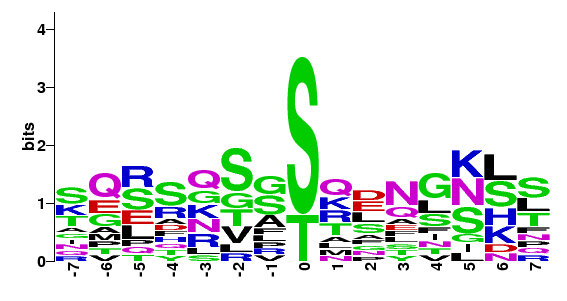

Supplement: Logos of 300 phosphorylation motifs predicted [file msb201312-s6.zip › Logo/MARK3.png]

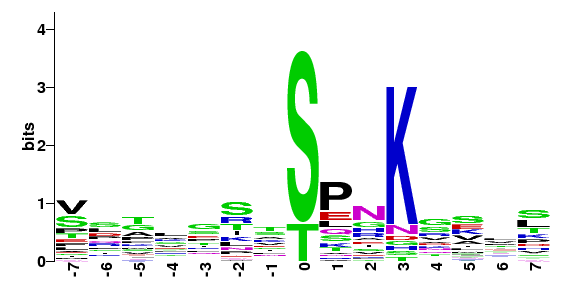

Supplement: Logos of 300 phosphorylation motifs predicted [file msb201312-s6.zip › Logo/MAST1.png]

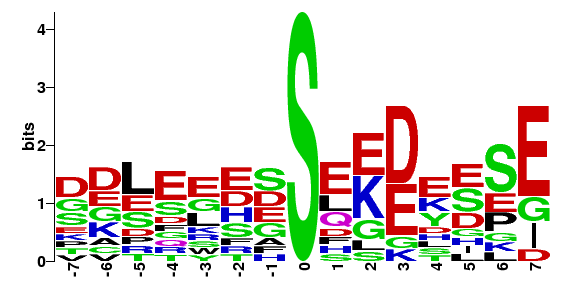

Supplement: Logos of 300 phosphorylation motifs predicted [file msb201312-s6.zip › Logo/MAST2.png]

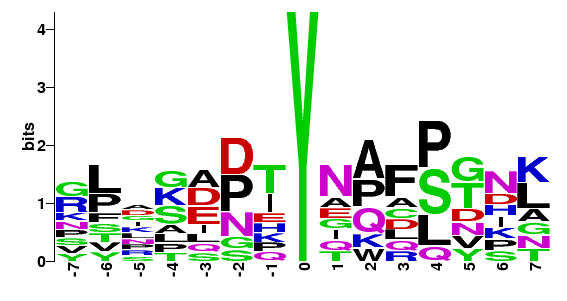

Supplement: Logos of 300 phosphorylation motifs predicted [file msb201312-s6.zip › Logo/MATK.png]

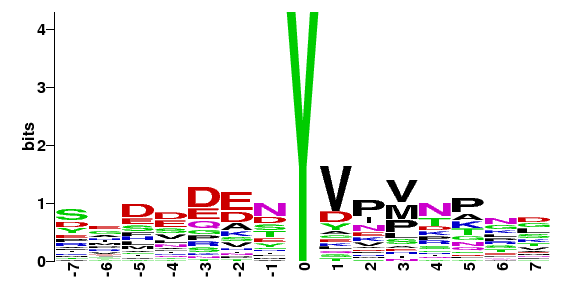

Supplement: Logos of 300 phosphorylation motifs predicted [file msb201312-s6.zip › Logo/MET.png]

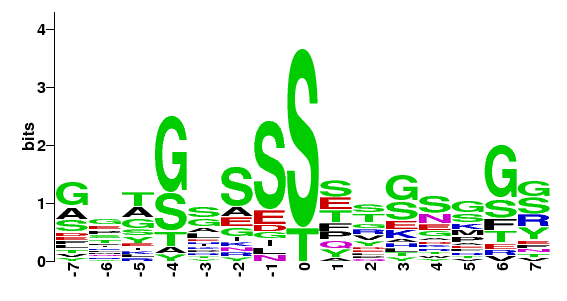

Supplement: Logos of 300 phosphorylation motifs predicted [file msb201312-s6.zip › Logo/MKNK1.png]

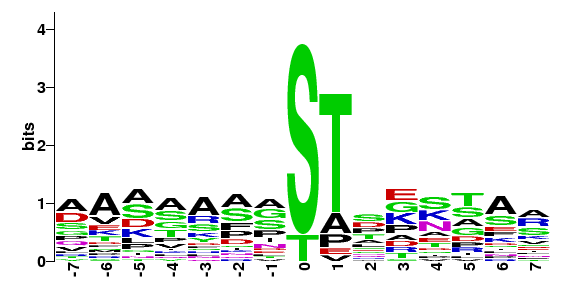

Supplement: Logos of 300 phosphorylation motifs predicted [file msb201312-s6.zip › Logo/MKNK2.png]

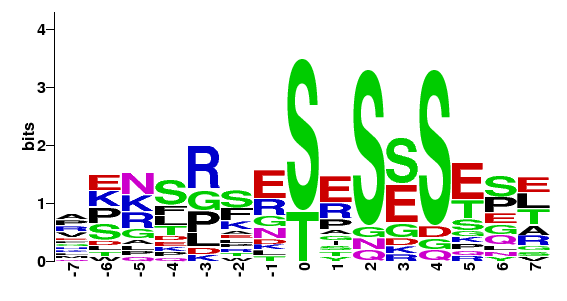

Supplement: Logos of 300 phosphorylation motifs predicted [file msb201312-s6.zip › Logo/MLKL.png]

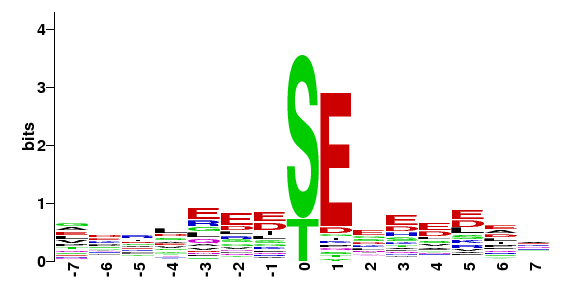

Supplement: Logos of 300 phosphorylation motifs predicted [file msb201312-s6.zip › Logo/MOS.png]

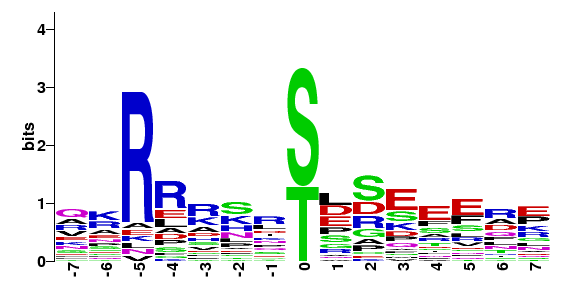

Supplement: Logos of 300 phosphorylation motifs predicted [file msb201312-s6.zip › Logo/MST4.png]

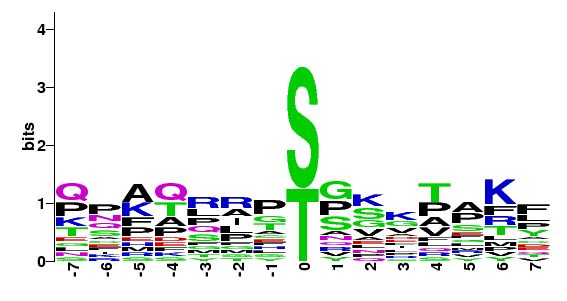

Supplement: Logos of 300 phosphorylation motifs predicted [file msb201312-s6.zip › Logo/MYLK.png]

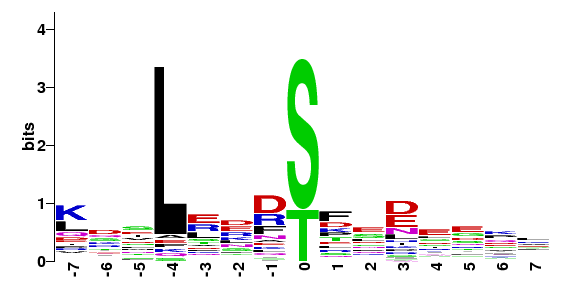

Supplement: Logos of 300 phosphorylation motifs predicted [file msb201312-s6.zip › Logo/MYLK2.png]

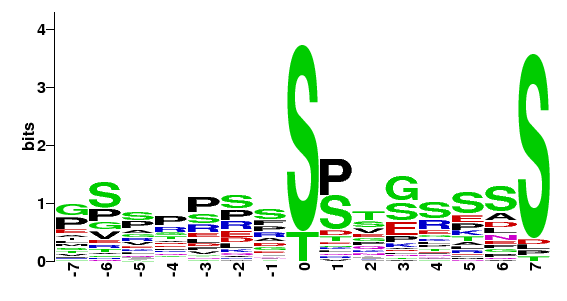

Supplement: Logos of 300 phosphorylation motifs predicted [file msb201312-s6.zip › Logo/MYO3A.png]

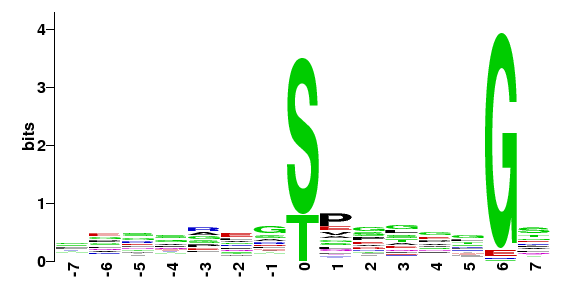

Supplement: Logos of 300 phosphorylation motifs predicted [file msb201312-s6.zip › Logo/NEK10.png]

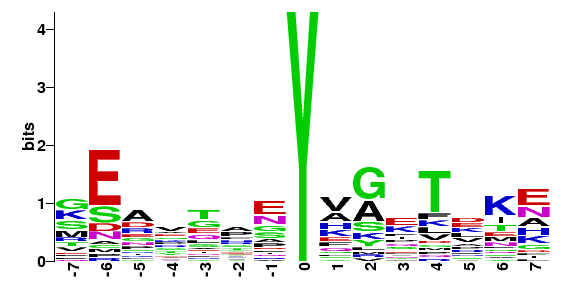

Supplement: Logos of 300 phosphorylation motifs predicted [file msb201312-s6.zip › Logo/NEK10_Y.png]

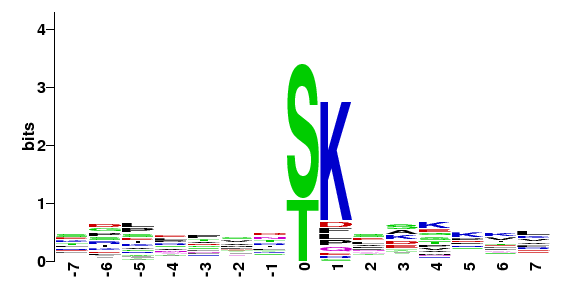

Supplement: Logos of 300 phosphorylation motifs predicted [file msb201312-s6.zip › Logo/NEK2.png]

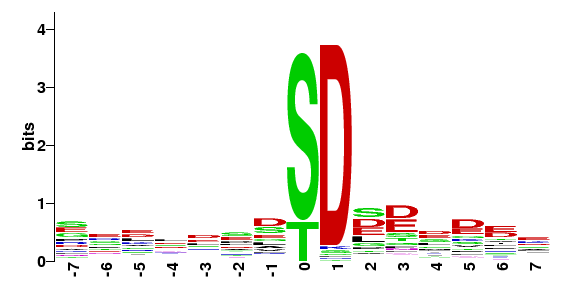

Supplement: Logos of 300 phosphorylation motifs predicted [file msb201312-s6.zip › Logo/NEK3.png]

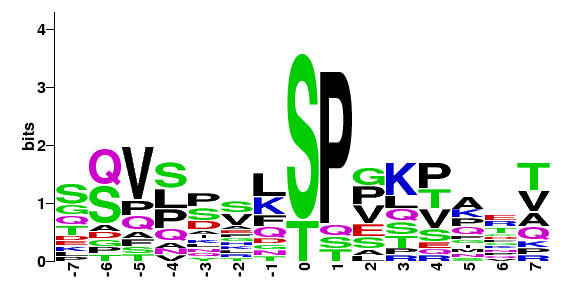

Supplement: Logos of 300 phosphorylation motifs predicted [file msb201312-s6.zip › Logo/NEK4.png]

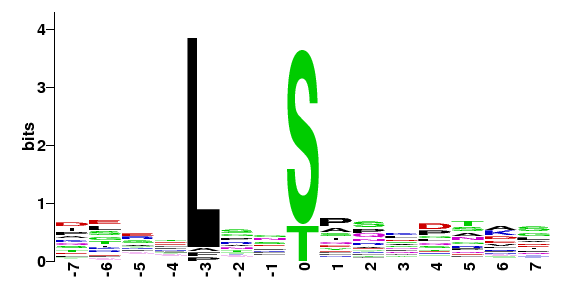

Supplement: Logos of 300 phosphorylation motifs predicted [file msb201312-s6.zip › Logo/NEK6.png]

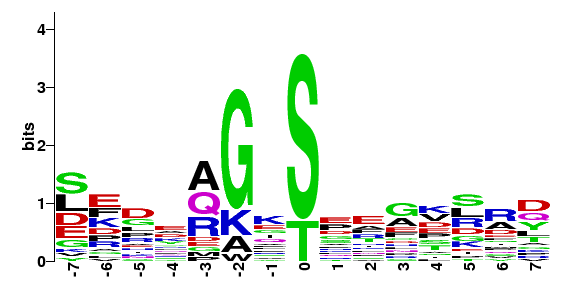

Supplement: Logos of 300 phosphorylation motifs predicted [file msb201312-s6.zip › Logo/NEK7.png]

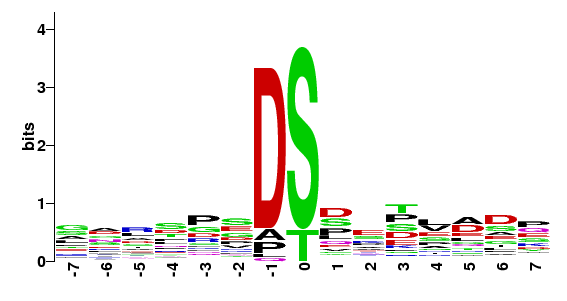

Supplement: Logos of 300 phosphorylation motifs predicted [file msb201312-s6.zip › Logo/NEK8.png]

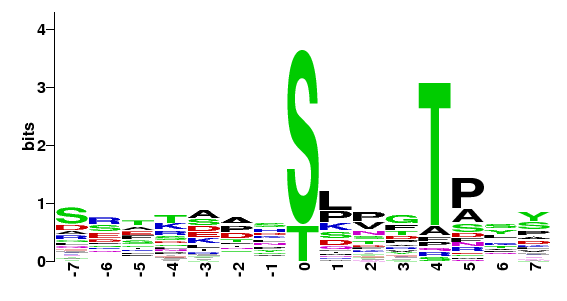

Supplement: Logos of 300 phosphorylation motifs predicted [file msb201312-s6.zip › Logo/NEK9.png]

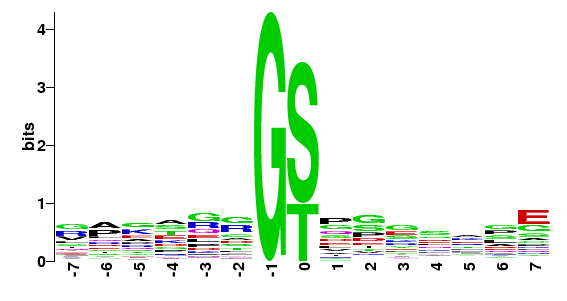

Supplement: Logos of 300 phosphorylation motifs predicted [file msb201312-s6.zip › Logo/NIM1.png]

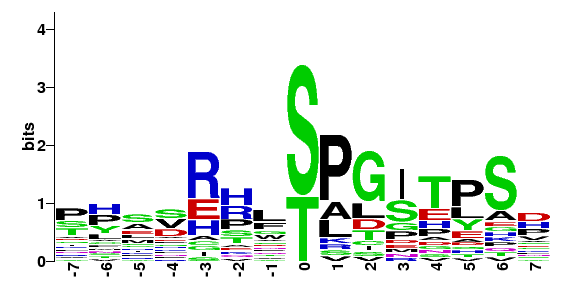

Supplement: Logos of 300 phosphorylation motifs predicted [file msb201312-s6.zip › Logo/NLK.png]

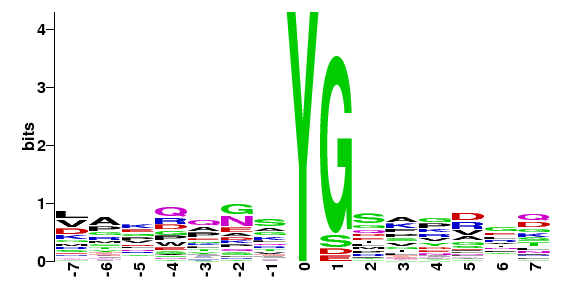

Supplement: Logos of 300 phosphorylation motifs predicted [file msb201312-s6.zip › Logo/NPR2.png]

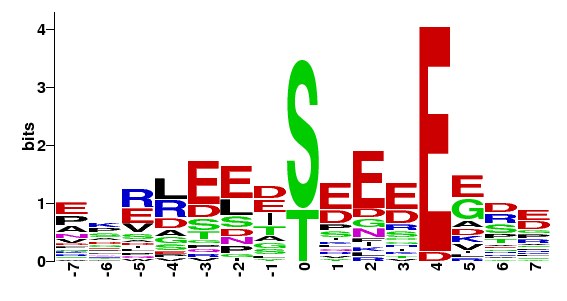

Supplement: Logos of 300 phosphorylation motifs predicted [file msb201312-s6.zip › Logo/NRBP1.png]

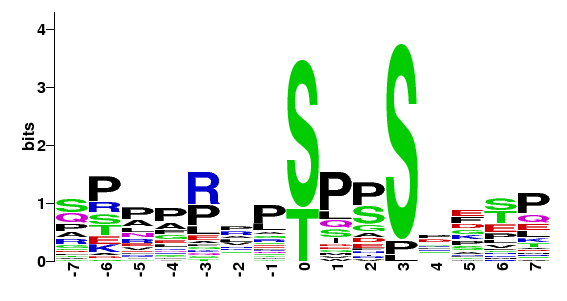

Supplement: Logos of 300 phosphorylation motifs predicted [file msb201312-s6.zip › Logo/NRBP2.png]

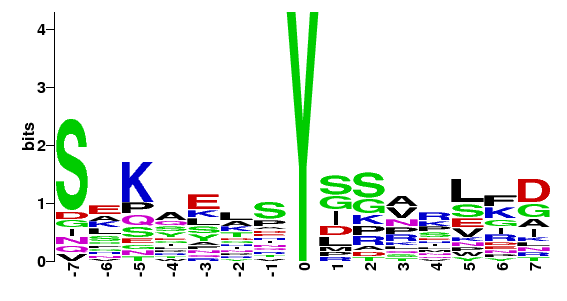

Supplement: Logos of 300 phosphorylation motifs predicted [file msb201312-s6.zip › Logo/NTRK3.png]

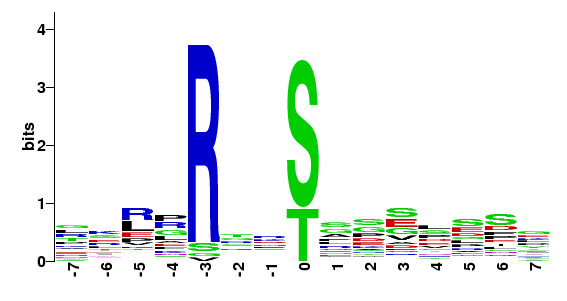

Supplement: Logos of 300 phosphorylation motifs predicted [file msb201312-s6.zip › Logo/NUAK1.png]

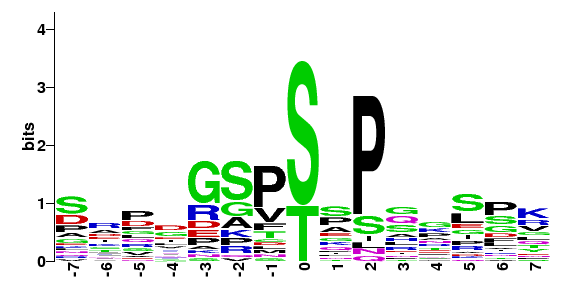

Supplement: Logos of 300 phosphorylation motifs predicted [file msb201312-s6.zip › Logo/NUAK2.png]

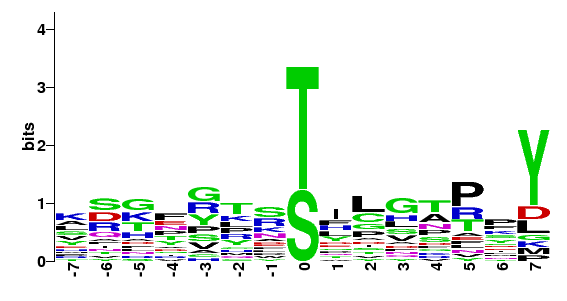

Supplement: Logos of 300 phosphorylation motifs predicted [file msb201312-s6.zip › Logo/OXSR1.png]

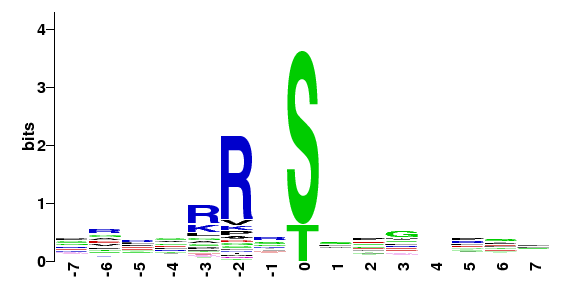

Supplement: Logos of 300 phosphorylation motifs predicted [file msb201312-s6.zip › Logo/PAK1.png]

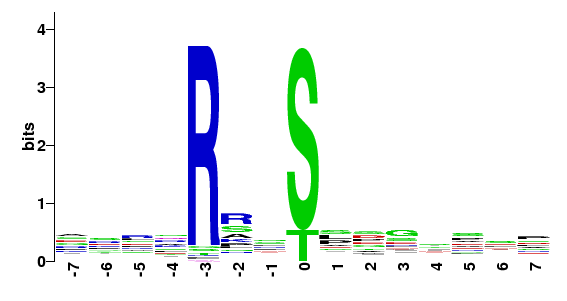

Supplement: Logos of 300 phosphorylation motifs predicted [file msb201312-s6.zip › Logo/PAK4.png]

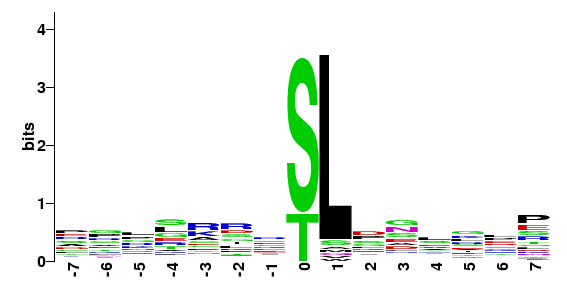

Supplement: Logos of 300 phosphorylation motifs predicted [file msb201312-s6.zip › Logo/PAK6.png]

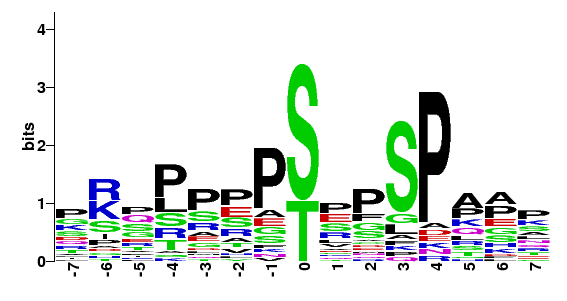

Supplement: Logos of 300 phosphorylation motifs predicted [file msb201312-s6.zip › Logo/PBK.png]

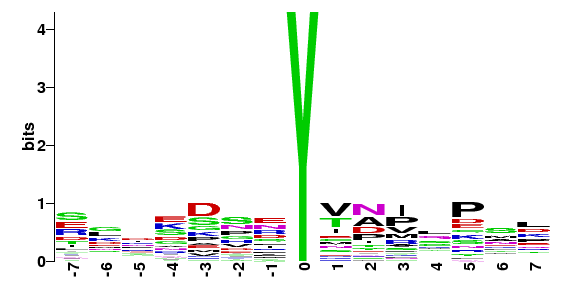

Supplement: Logos of 300 phosphorylation motifs predicted [file msb201312-s6.zip › Logo/PDGFRB.png]

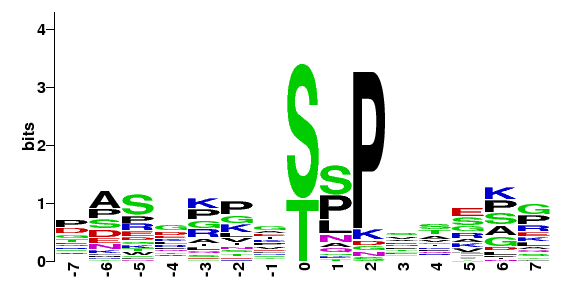

Supplement: Logos of 300 phosphorylation motifs predicted [file msb201312-s6.zip › Logo/PDIK1L.png]

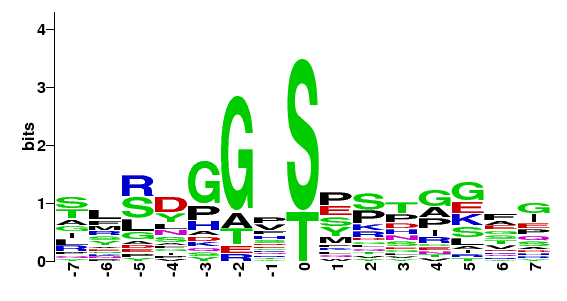

Supplement: Logos of 300 phosphorylation motifs predicted [file msb201312-s6.zip › Logo/PDK3.png]

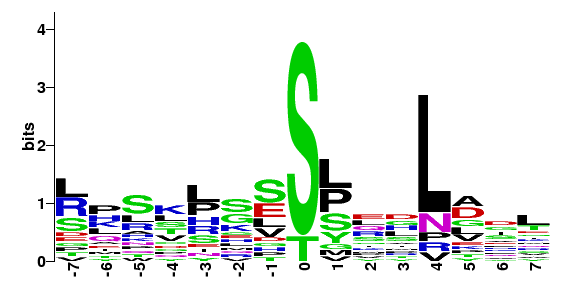

Supplement: Logos of 300 phosphorylation motifs predicted [file msb201312-s6.zip › Logo/PDK4.png]

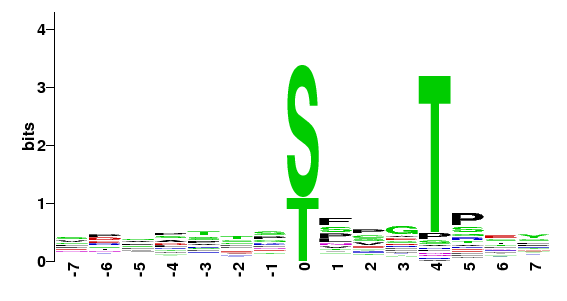

Supplement: Logos of 300 phosphorylation motifs predicted [file msb201312-s6.zip › Logo/PDPK1.png]

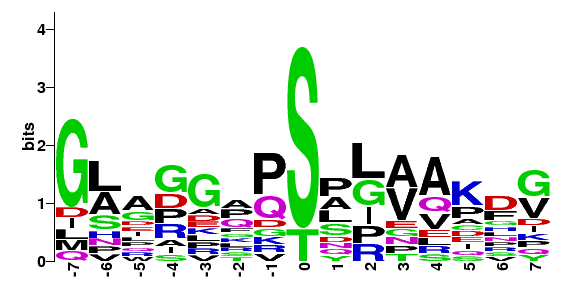

Supplement: Logos of 300 phosphorylation motifs predicted [file msb201312-s6.zip › Logo/PHKG2.png]

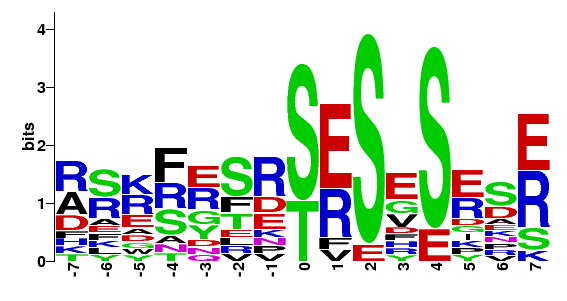

Supplement: Logos of 300 phosphorylation motifs predicted [file msb201312-s6.zip › Logo/PIK3C3.png]

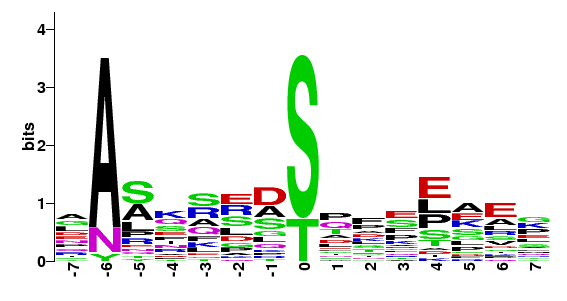

Supplement: Logos of 300 phosphorylation motifs predicted [file msb201312-s6.zip › Logo/PIK3R4.png]

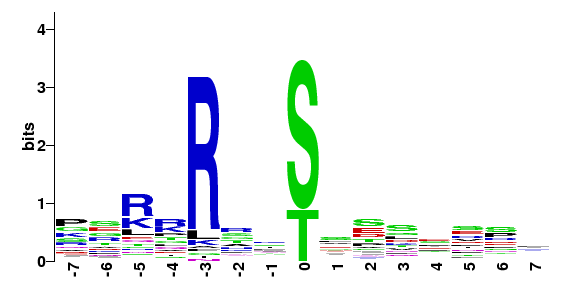

Supplement: Logos of 300 phosphorylation motifs predicted [file msb201312-s6.zip › Logo/PIM1.png]

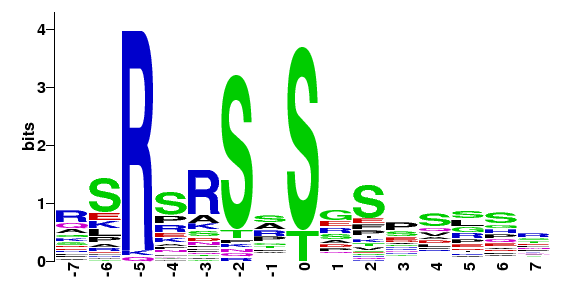

Supplement: Logos of 300 phosphorylation motifs predicted [file msb201312-s6.zip › Logo/PIM2.png]

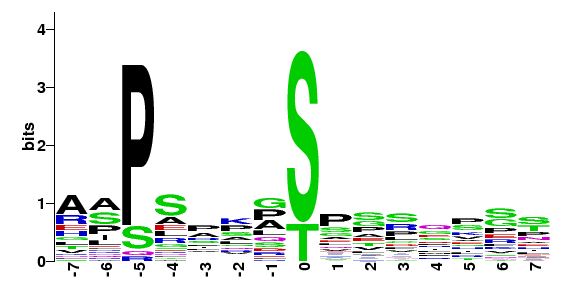

Supplement: Logos of 300 phosphorylation motifs predicted [file msb201312-s6.zip › Logo/PIM3.png]

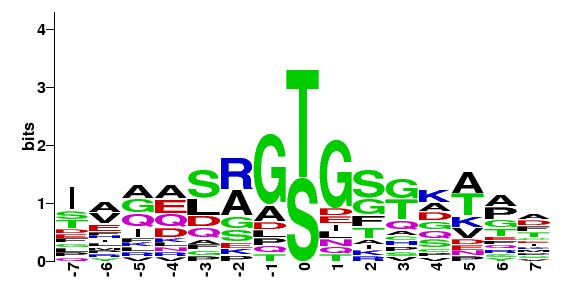

Supplement: Logos of 300 phosphorylation motifs predicted [file msb201312-s6.zip › Logo/PINK1.png]

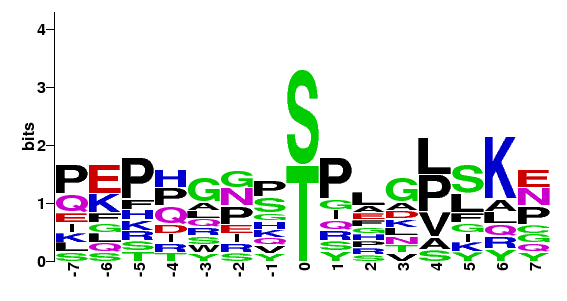

Supplement: Logos of 300 phosphorylation motifs predicted [file msb201312-s6.zip › Logo/PKMYT1.png]

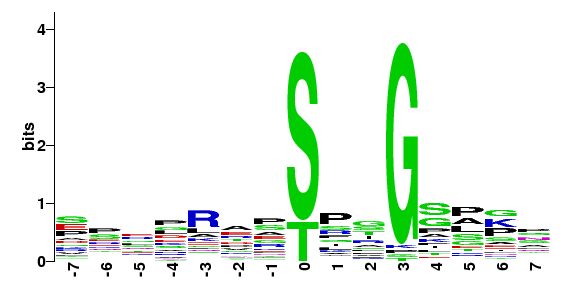

Supplement: Logos of 300 phosphorylation motifs predicted [file msb201312-s6.zip › Logo/PKN1.png]
