# Supplementary figures and images for: Construction of human activity-based phosphorylation networks (part 1 of 2)
Source: Mol Syst Biol. 2013 Apr 2;9:655. doi: 10.1038/msb.2013.12 (PMC3658267; doi:10.1038/msb.2013.12)

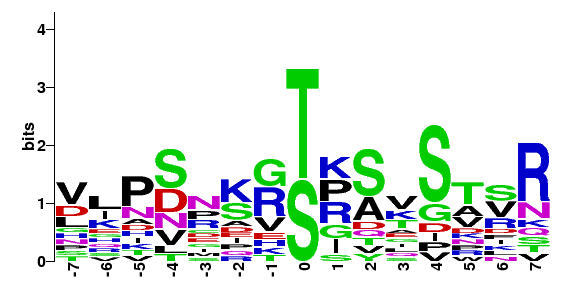

Supplement: Logos of 300 phosphorylation motifs predicted [file msb201312-s6.zip › Logo/AAK1.png]

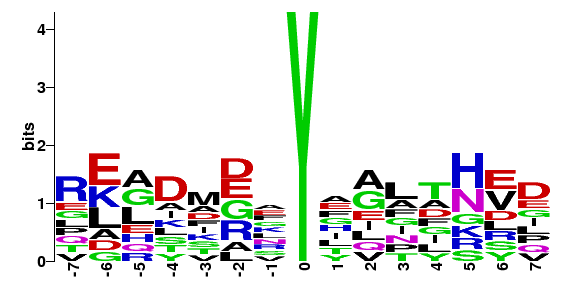

Supplement: Logos of 300 phosphorylation motifs predicted [file msb201312-s6.zip › Logo/AAK1_Y.png]

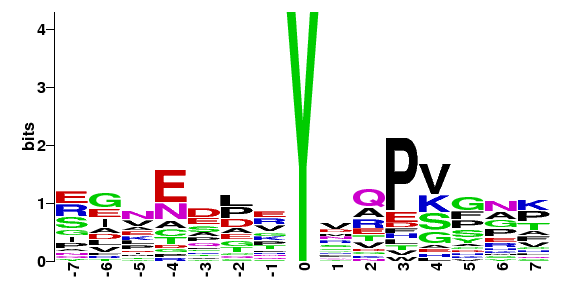

Supplement: Logos of 300 phosphorylation motifs predicted [file msb201312-s6.zip › Logo/ABL2.png]

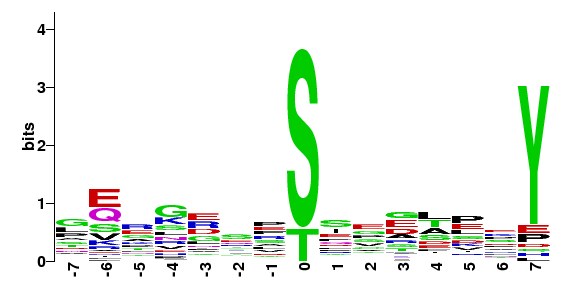

Supplement: Logos of 300 phosphorylation motifs predicted [file msb201312-s6.zip › Logo/ACVR1.png]

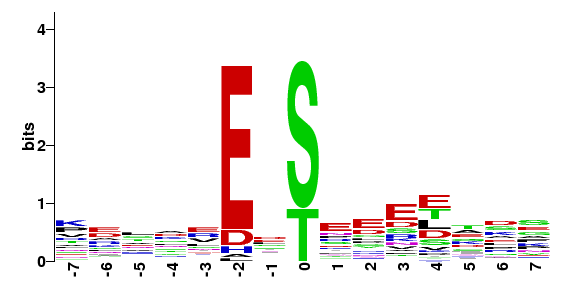

Supplement: Logos of 300 phosphorylation motifs predicted [file msb201312-s6.zip › Logo/ACVR1B.png]

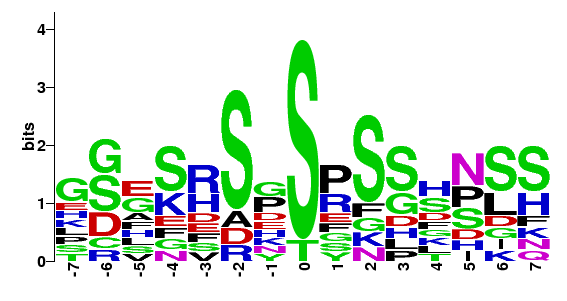

Supplement: Logos of 300 phosphorylation motifs predicted [file msb201312-s6.zip › Logo/ACVR2A.png]

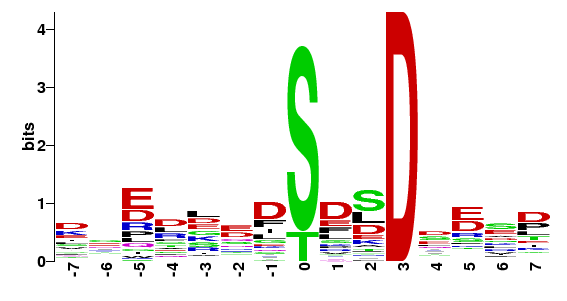

Supplement: Logos of 300 phosphorylation motifs predicted [file msb201312-s6.zip › Logo/ACVR2B.png]

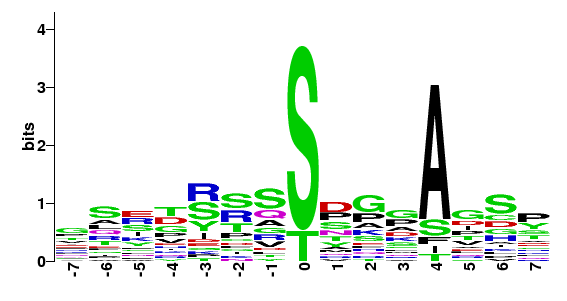

Supplement: Logos of 300 phosphorylation motifs predicted [file msb201312-s6.zip › Logo/ACVRL1.png]

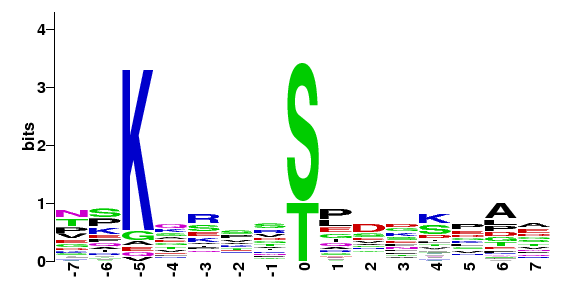

Supplement: Logos of 300 phosphorylation motifs predicted [file msb201312-s6.zip › Logo/ADCK1.png]

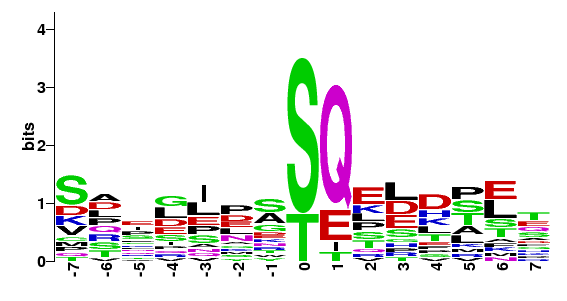

Supplement: Logos of 300 phosphorylation motifs predicted [file msb201312-s6.zip › Logo/ADCK3.png]

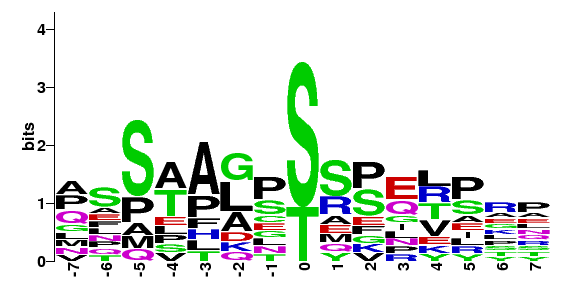

Supplement: Logos of 300 phosphorylation motifs predicted [file msb201312-s6.zip › Logo/ADCK5.png]

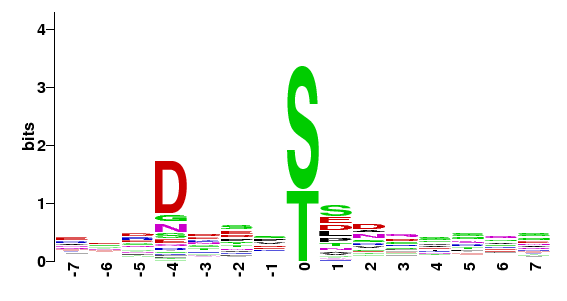

Supplement: Logos of 300 phosphorylation motifs predicted [file msb201312-s6.zip › Logo/ADRBK1.png]

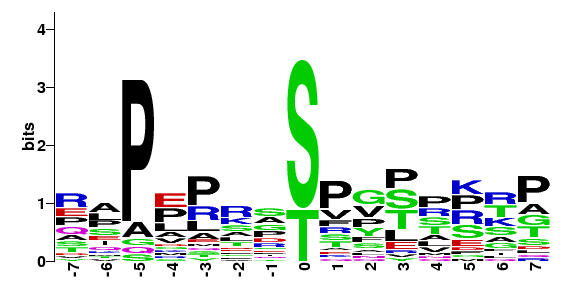

Supplement: Logos of 300 phosphorylation motifs predicted [file msb201312-s6.zip › Logo/ADRBK2.png]

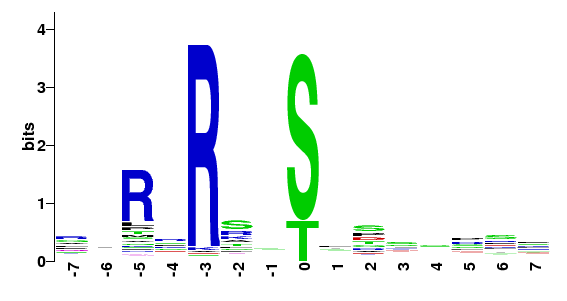

Supplement: Logos of 300 phosphorylation motifs predicted [file msb201312-s6.zip › Logo/AKT1.png]

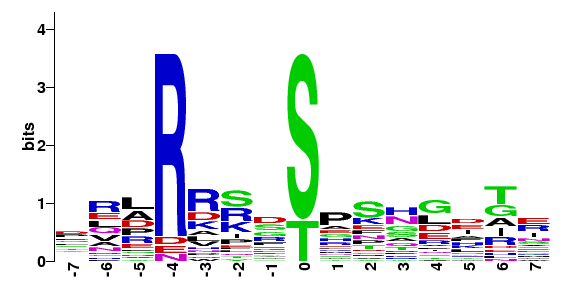

Supplement: Logos of 300 phosphorylation motifs predicted [file msb201312-s6.zip › Logo/AKT3.png]

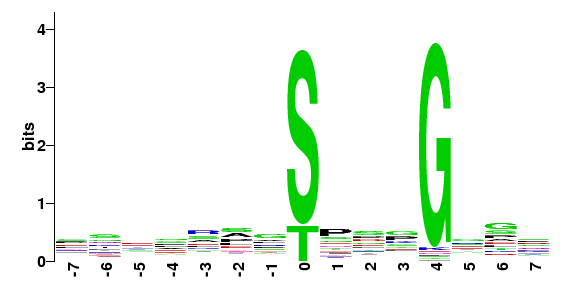

Supplement: Logos of 300 phosphorylation motifs predicted [file msb201312-s6.zip › Logo/ARAF.png]

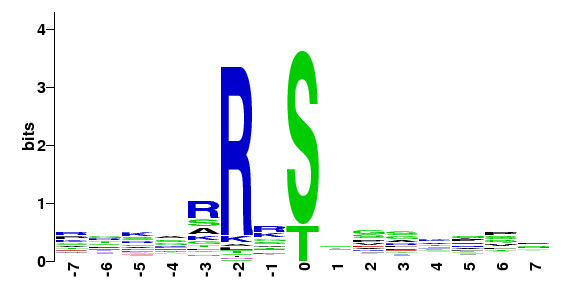

Supplement: Logos of 300 phosphorylation motifs predicted [file msb201312-s6.zip › Logo/AURKB.png]

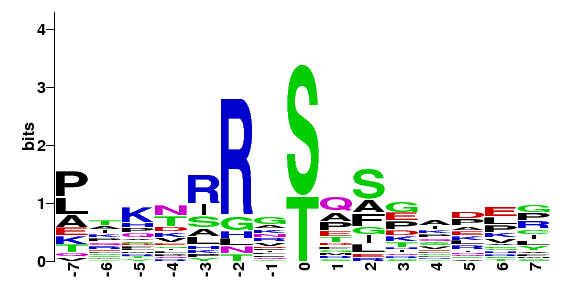

Supplement: Logos of 300 phosphorylation motifs predicted [file msb201312-s6.zip › Logo/AURKC.png]

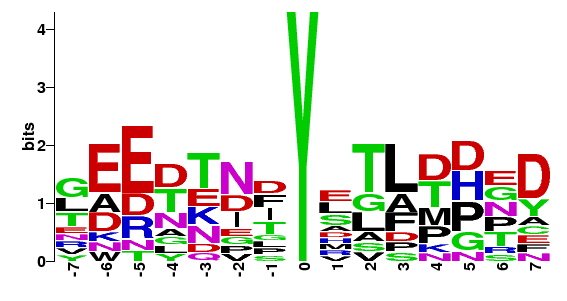

Supplement: Logos of 300 phosphorylation motifs predicted [file msb201312-s6.zip › Logo/BLK.png]

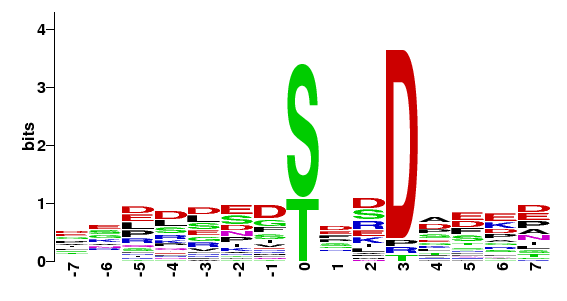

Supplement: Logos of 300 phosphorylation motifs predicted [file msb201312-s6.zip › Logo/BMP2K.png]

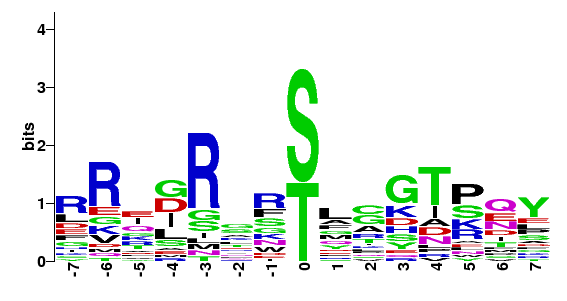

Supplement: Logos of 300 phosphorylation motifs predicted [file msb201312-s6.zip › Logo/BMPR1A.png]

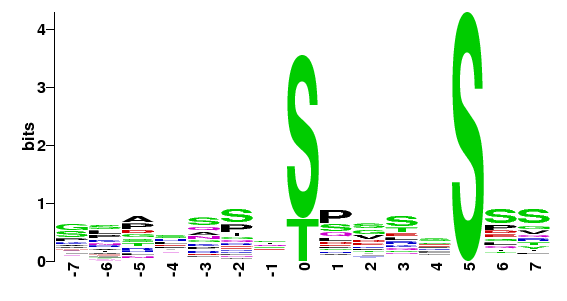

Supplement: Logos of 300 phosphorylation motifs predicted [file msb201312-s6.zip › Logo/BMPR1B.png]

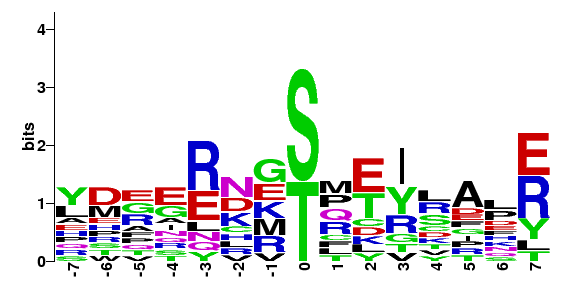

Supplement: Logos of 300 phosphorylation motifs predicted [file msb201312-s6.zip › Logo/BMPR2.png]

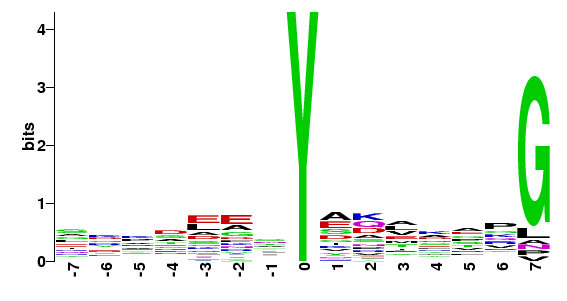

Supplement: Logos of 300 phosphorylation motifs predicted [file msb201312-s6.zip › Logo/BMX.png]

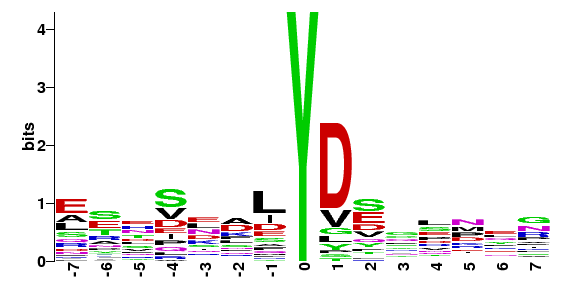

Supplement: Logos of 300 phosphorylation motifs predicted [file msb201312-s6.zip › Logo/BTK.png]

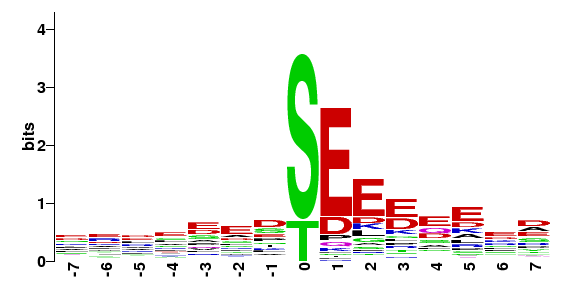

Supplement: Logos of 300 phosphorylation motifs predicted [file msb201312-s6.zip › Logo/BUB1.png]

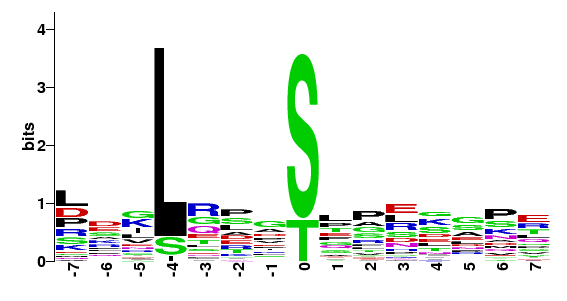

Supplement: Logos of 300 phosphorylation motifs predicted [file msb201312-s6.zip › Logo/CAMK1D.png]

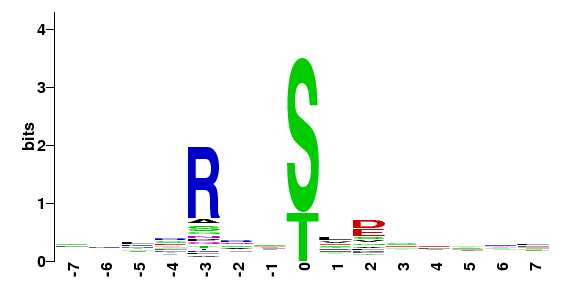

Supplement: Logos of 300 phosphorylation motifs predicted [file msb201312-s6.zip › Logo/CAMK2A.png]

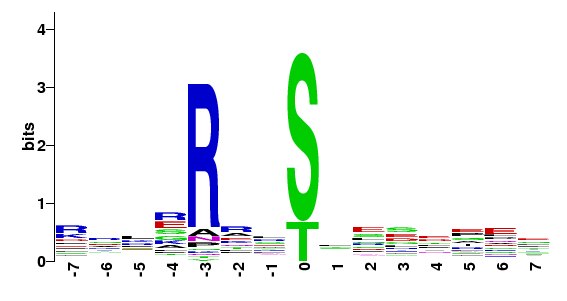

Supplement: Logos of 300 phosphorylation motifs predicted [file msb201312-s6.zip › Logo/CAMK2B.png]

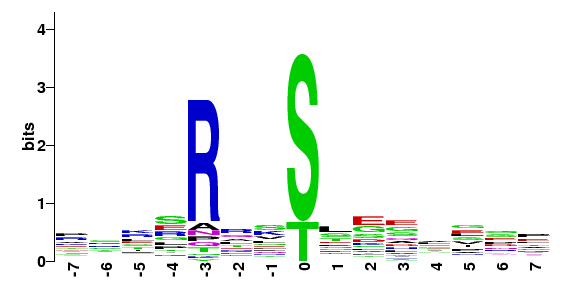

Supplement: Logos of 300 phosphorylation motifs predicted [file msb201312-s6.zip › Logo/CAMK2D.png]

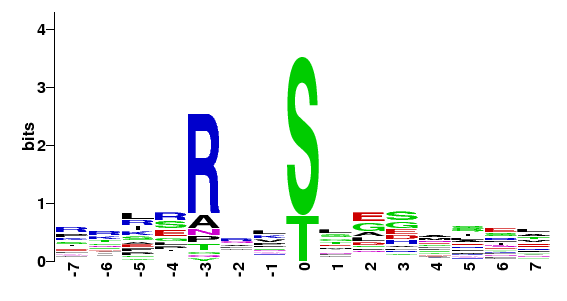

Supplement: Logos of 300 phosphorylation motifs predicted [file msb201312-s6.zip › Logo/CAMK2G.png]

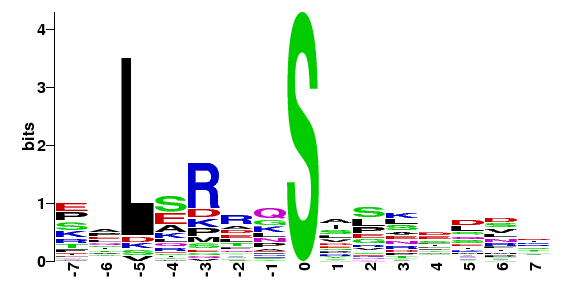

Supplement: Logos of 300 phosphorylation motifs predicted [file msb201312-s6.zip › Logo/CAMK4.png]

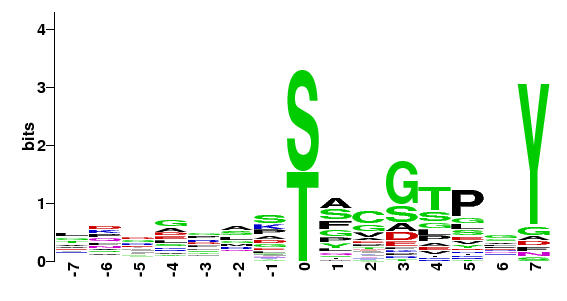

Supplement: Logos of 300 phosphorylation motifs predicted [file msb201312-s6.zip › Logo/CAMKK1.png]

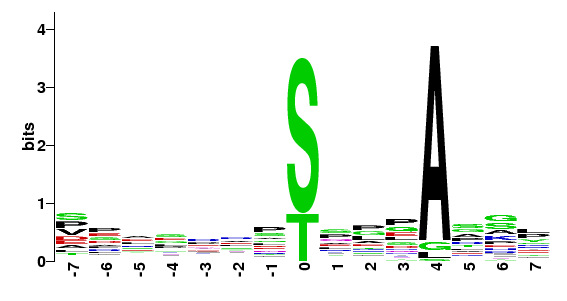

Supplement: Logos of 300 phosphorylation motifs predicted [file msb201312-s6.zip › Logo/CAMKK2.png]

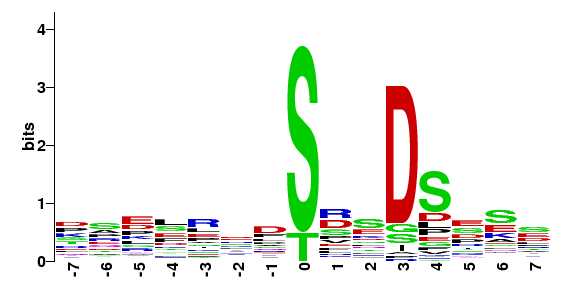

Supplement: Logos of 300 phosphorylation motifs predicted [file msb201312-s6.zip › Logo/CAMKV.png]

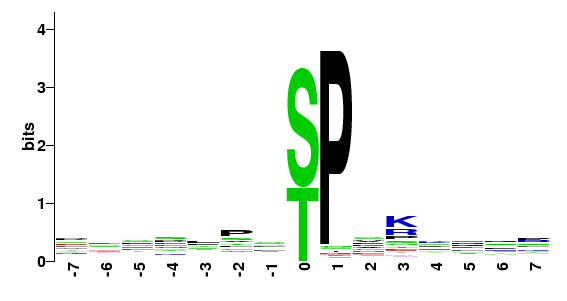

Supplement: Logos of 300 phosphorylation motifs predicted [file msb201312-s6.zip › Logo/CDK1.png]

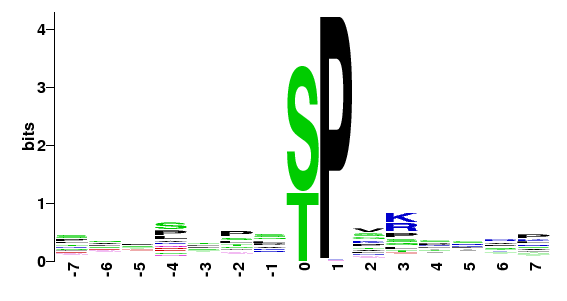

Supplement: Logos of 300 phosphorylation motifs predicted [file msb201312-s6.zip › Logo/CDK10.png]

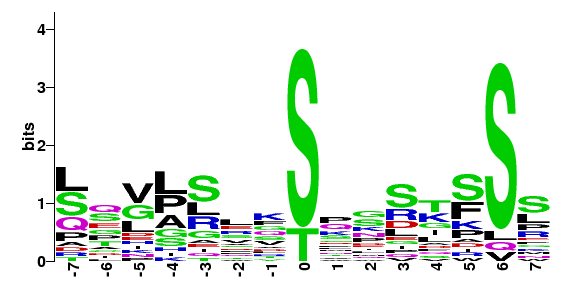

Supplement: Logos of 300 phosphorylation motifs predicted [file msb201312-s6.zip › Logo/CDK13.png]

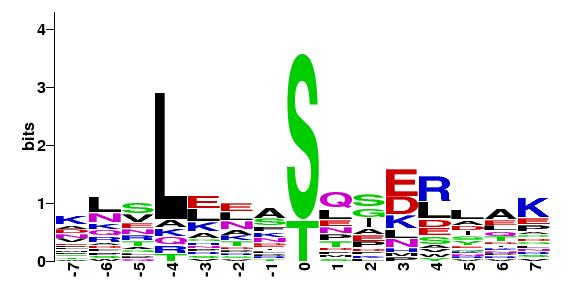

Supplement: Logos of 300 phosphorylation motifs predicted [file msb201312-s6.zip › Logo/CDK14.png]

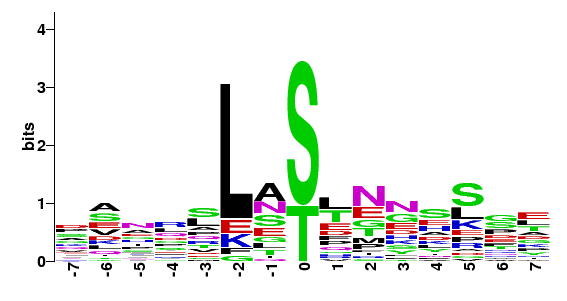

Supplement: Logos of 300 phosphorylation motifs predicted [file msb201312-s6.zip › Logo/CDK15.png]

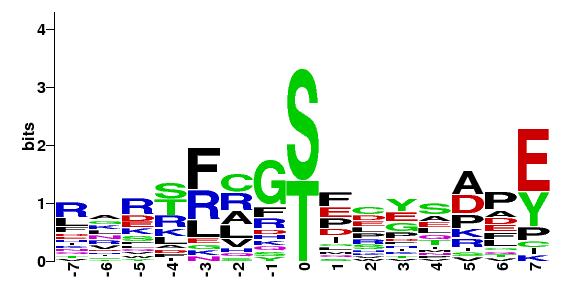

Supplement: Logos of 300 phosphorylation motifs predicted [file msb201312-s6.zip › Logo/CDK16.png]

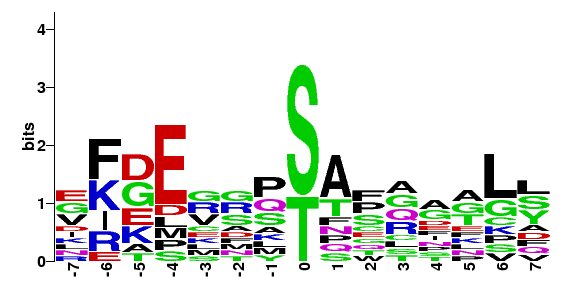

Supplement: Logos of 300 phosphorylation motifs predicted [file msb201312-s6.zip › Logo/CDK17.png]

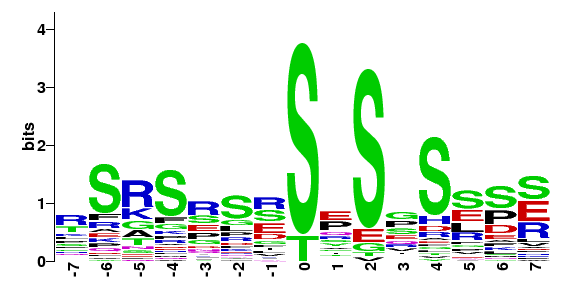

Supplement: Logos of 300 phosphorylation motifs predicted [file msb201312-s6.zip › Logo/CDK18.png]

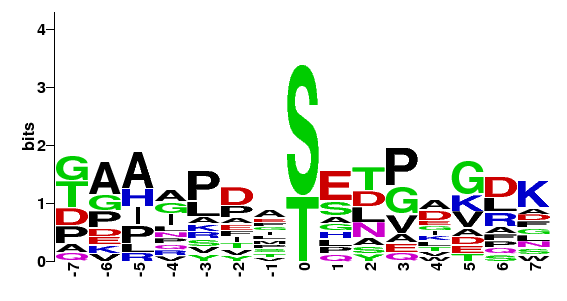

Supplement: Logos of 300 phosphorylation motifs predicted [file msb201312-s6.zip › Logo/CDK19.png]

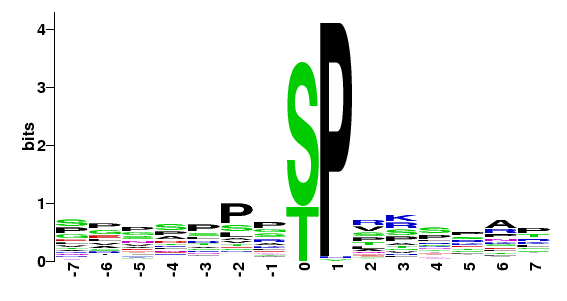

Supplement: Logos of 300 phosphorylation motifs predicted [file msb201312-s6.zip › Logo/CDK3.png]

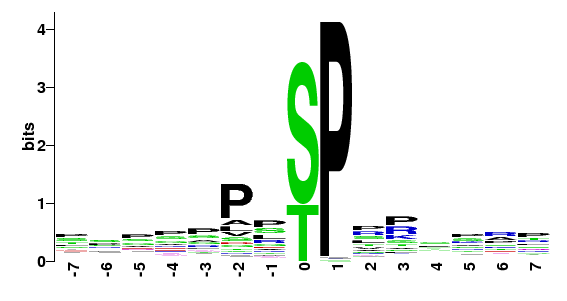

Supplement: Logos of 300 phosphorylation motifs predicted [file msb201312-s6.zip › Logo/CDK4.png]

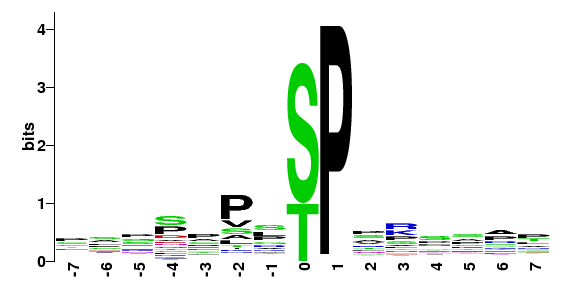

Supplement: Logos of 300 phosphorylation motifs predicted [file msb201312-s6.zip › Logo/CDK5.png]

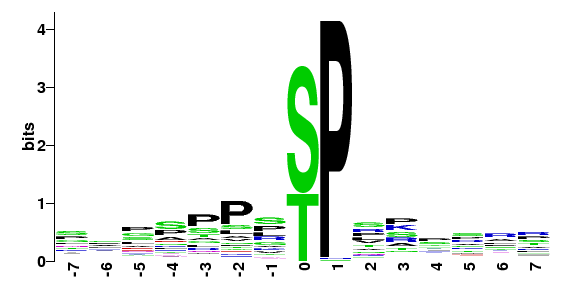

Supplement: Logos of 300 phosphorylation motifs predicted [file msb201312-s6.zip › Logo/CDK6.png]

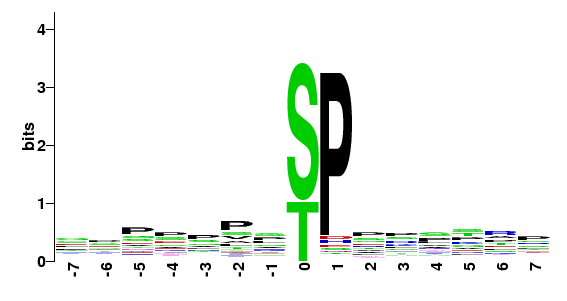

Supplement: Logos of 300 phosphorylation motifs predicted [file msb201312-s6.zip › Logo/CDK7.png]

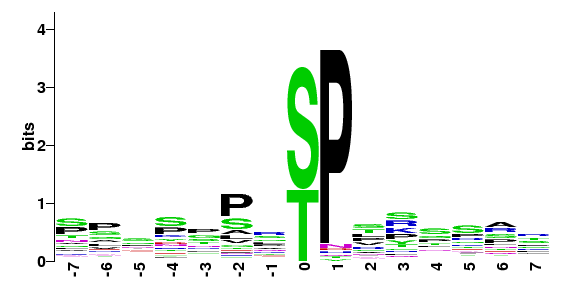

Supplement: Logos of 300 phosphorylation motifs predicted [file msb201312-s6.zip › Logo/CDK9.png]

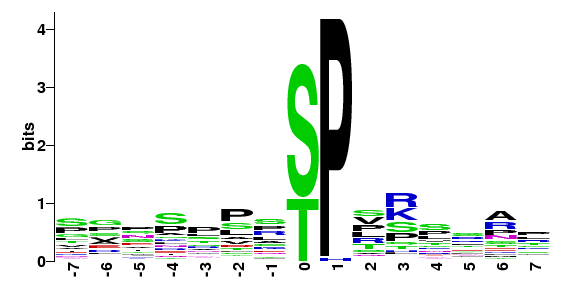

Supplement: Logos of 300 phosphorylation motifs predicted [file msb201312-s6.zip › Logo/CDKL3.png]

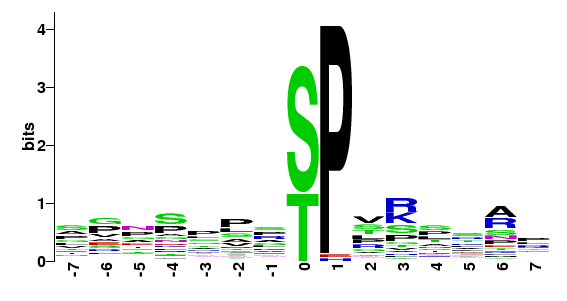

Supplement: Logos of 300 phosphorylation motifs predicted [file msb201312-s6.zip › Logo/CDKL5.png]

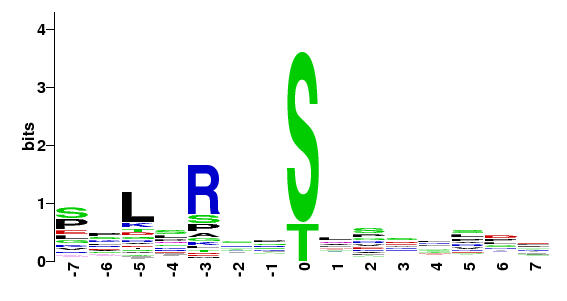

Supplement: Logos of 300 phosphorylation motifs predicted [file msb201312-s6.zip › Logo/CHEK2.png]

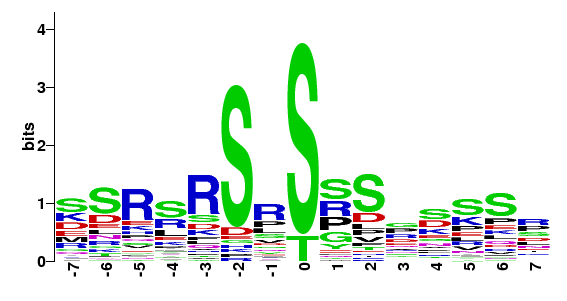

Supplement: Logos of 300 phosphorylation motifs predicted [file msb201312-s6.zip › Logo/CLK1.png]

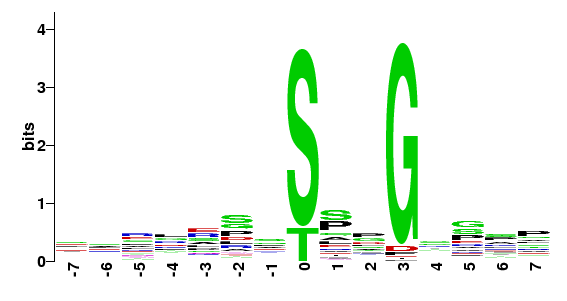

Supplement: Logos of 300 phosphorylation motifs predicted [file msb201312-s6.zip › Logo/CLK2.png]

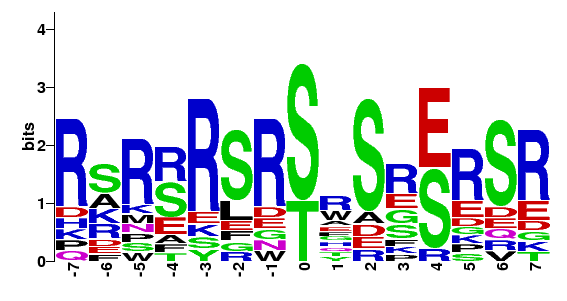

Supplement: Logos of 300 phosphorylation motifs predicted [file msb201312-s6.zip › Logo/CLK3.png]

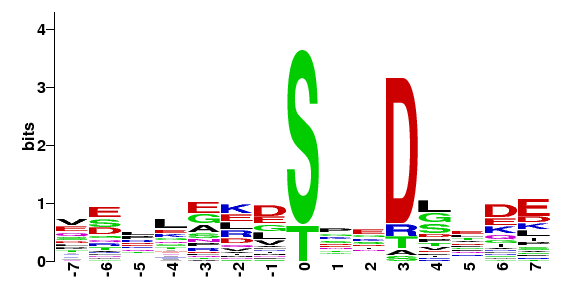

Supplement: Logos of 300 phosphorylation motifs predicted [file msb201312-s6.zip › Logo/CLK4.png]

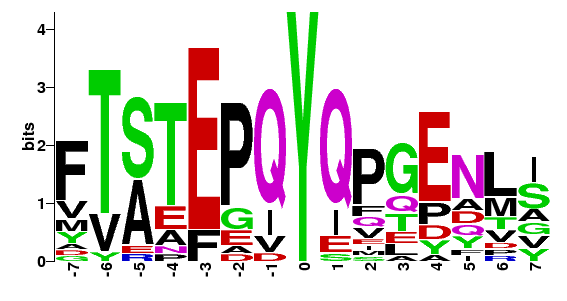

Supplement: Logos of 300 phosphorylation motifs predicted [file msb201312-s6.zip › Logo/CSK.png]

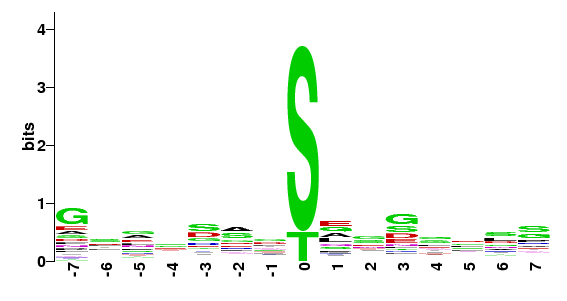

Supplement: Logos of 300 phosphorylation motifs predicted [file msb201312-s6.zip › Logo/CSNK1A1.png]

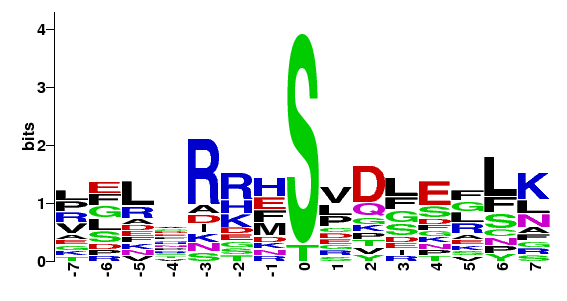

Supplement: Logos of 300 phosphorylation motifs predicted [file msb201312-s6.zip › Logo/CSNK1A1L.png]

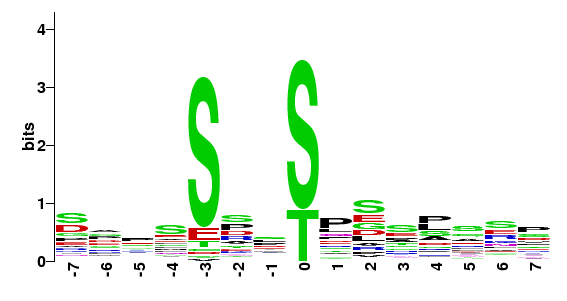

Supplement: Logos of 300 phosphorylation motifs predicted [file msb201312-s6.zip › Logo/CSNK1D.png]

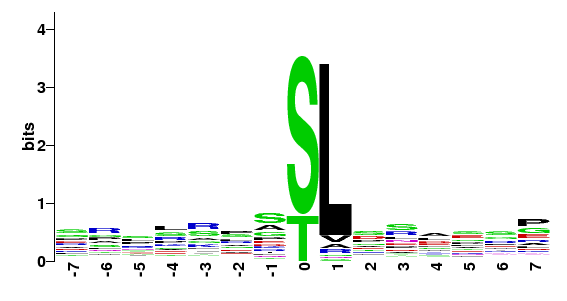

Supplement: Logos of 300 phosphorylation motifs predicted [file msb201312-s6.zip › Logo/CSNK1E.png]

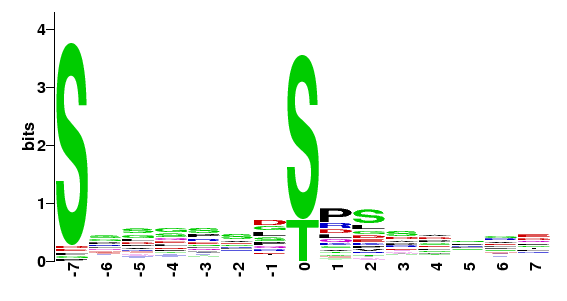

Supplement: Logos of 300 phosphorylation motifs predicted [file msb201312-s6.zip › Logo/CSNK1G1.png]

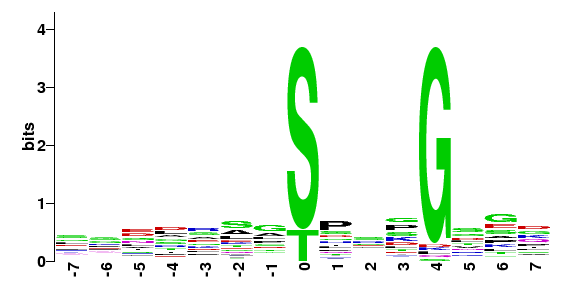

Supplement: Logos of 300 phosphorylation motifs predicted [file msb201312-s6.zip › Logo/CSNK1G2.png]

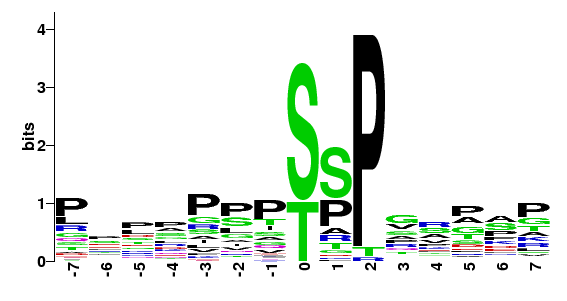

Supplement: Logos of 300 phosphorylation motifs predicted [file msb201312-s6.zip › Logo/CSNK1G3.png]

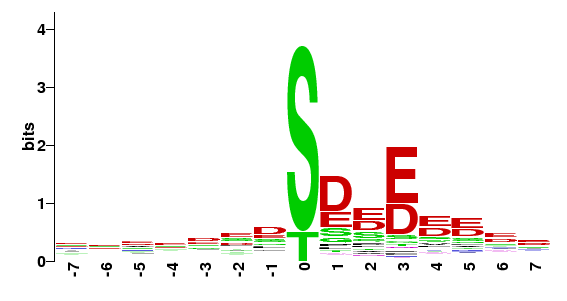

Supplement: Logos of 300 phosphorylation motifs predicted [file msb201312-s6.zip › Logo/CSNK2A1.png]

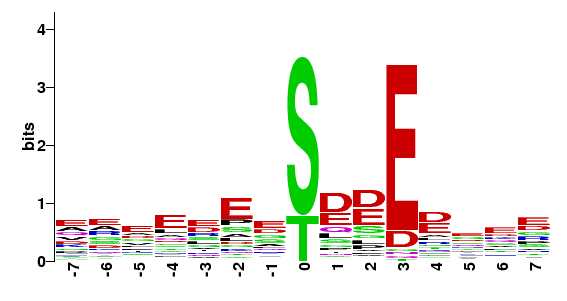

Supplement: Logos of 300 phosphorylation motifs predicted [file msb201312-s6.zip › Logo/CSNK2A2.png]

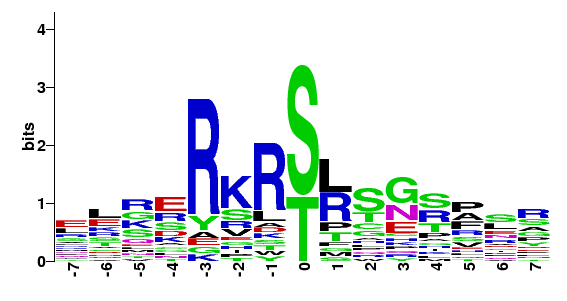

Supplement: Logos of 300 phosphorylation motifs predicted [file msb201312-s6.zip › Logo/DCLK1.png]

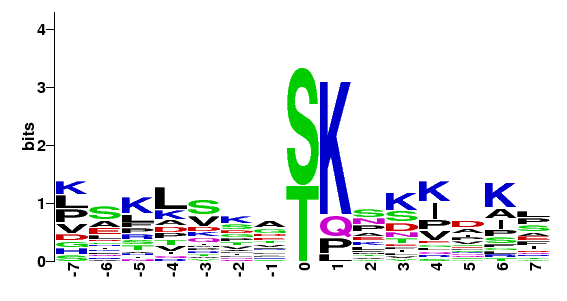

Supplement: Logos of 300 phosphorylation motifs predicted [file msb201312-s6.zip › Logo/DCLK2.png]

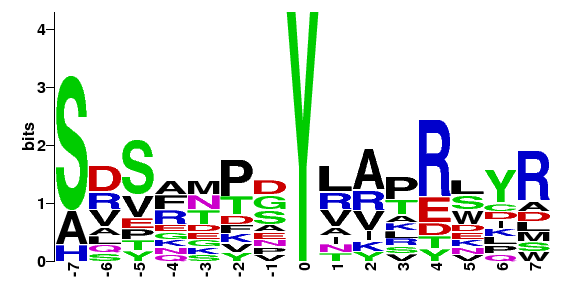

Supplement: Logos of 300 phosphorylation motifs predicted [file msb201312-s6.zip › Logo/DDR1.png]

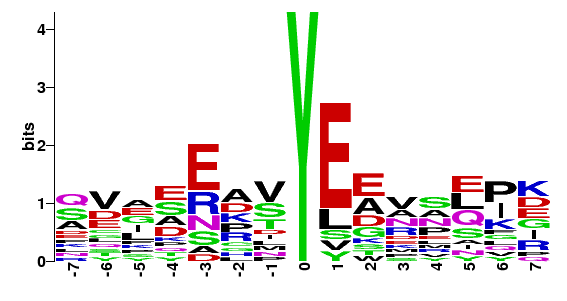

Supplement: Logos of 300 phosphorylation motifs predicted [file msb201312-s6.zip › Logo/DDR2.png]

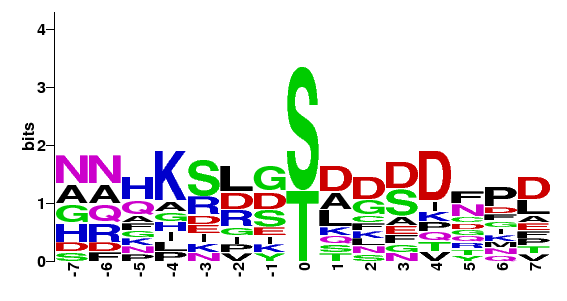

Supplement: Logos of 300 phosphorylation motifs predicted [file msb201312-s6.zip › Logo/DMPK.png]

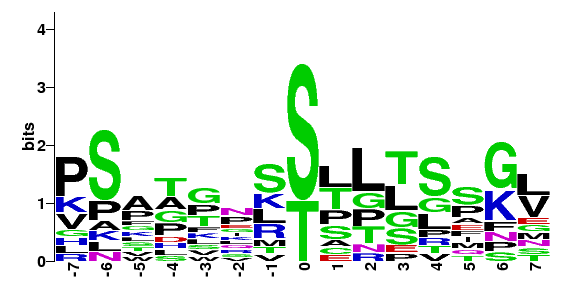

Supplement: Logos of 300 phosphorylation motifs predicted [file msb201312-s6.zip › Logo/DSTYK.png]

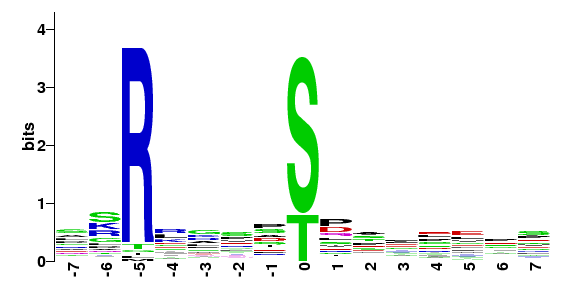

Supplement: Logos of 300 phosphorylation motifs predicted [file msb201312-s6.zip › Logo/DYRK1B.png]

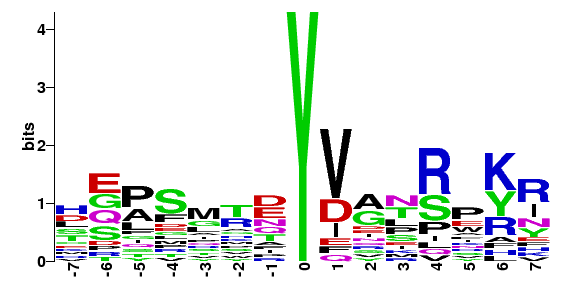

Supplement: Logos of 300 phosphorylation motifs predicted [file msb201312-s6.zip › Logo/DYRK1B_Y.png]

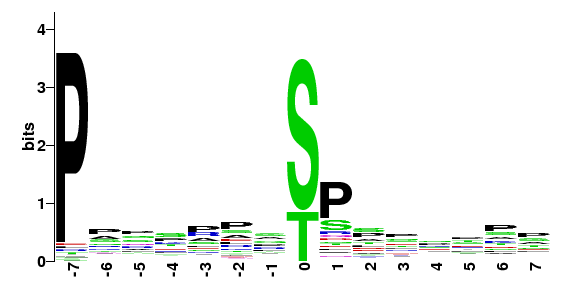

Supplement: Logos of 300 phosphorylation motifs predicted [file msb201312-s6.zip › Logo/DYRK2.png]

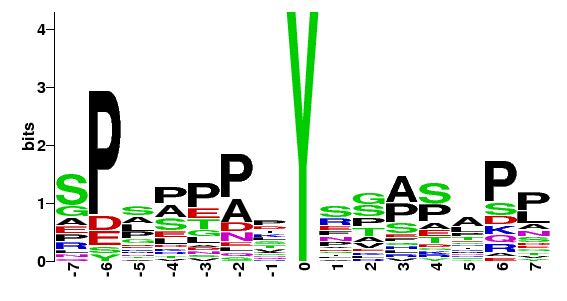

Supplement: Logos of 300 phosphorylation motifs predicted [file msb201312-s6.zip › Logo/DYRK2_Y.png]

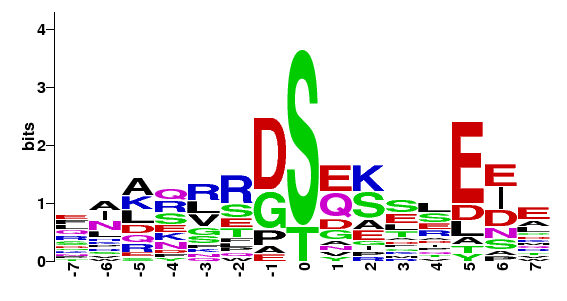

Supplement: Logos of 300 phosphorylation motifs predicted [file msb201312-s6.zip › Logo/DYRK3.png]

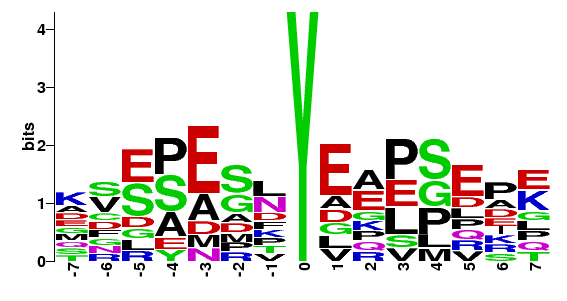

Supplement: Logos of 300 phosphorylation motifs predicted [file msb201312-s6.zip › Logo/DYRK3_Y.png]

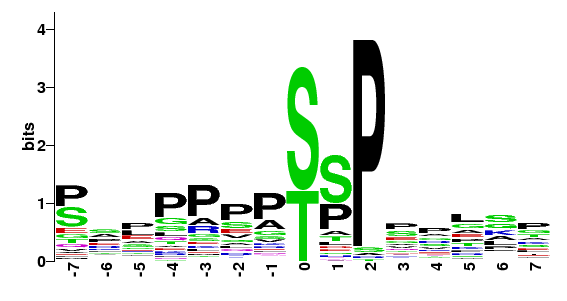

Supplement: Logos of 300 phosphorylation motifs predicted [file msb201312-s6.zip › Logo/DYRK4.png]

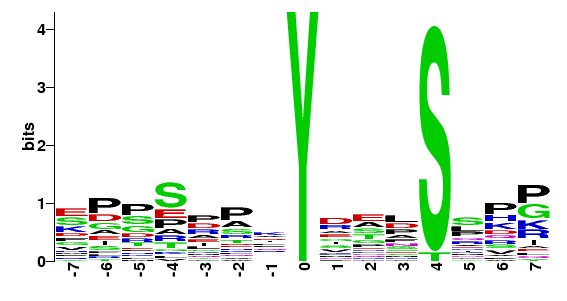

Supplement: Logos of 300 phosphorylation motifs predicted [file msb201312-s6.zip › Logo/DYRK4_Y.png]

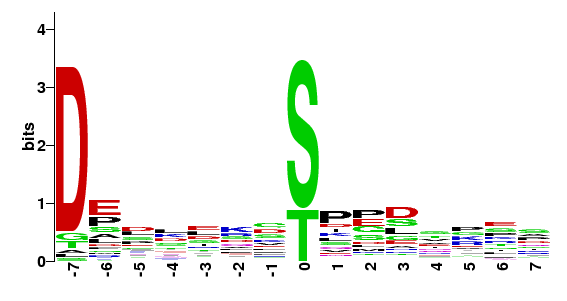

Supplement: Logos of 300 phosphorylation motifs predicted [file msb201312-s6.zip › Logo/EEF2K.png]

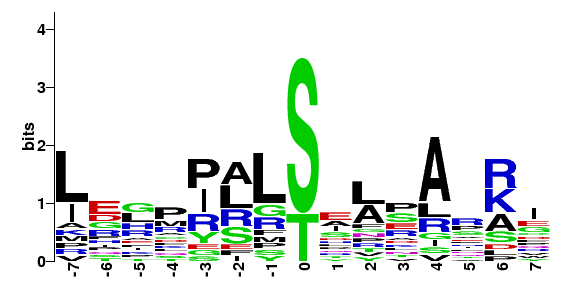

Supplement: Logos of 300 phosphorylation motifs predicted [file msb201312-s6.zip › Logo/EIF2AK1.png]

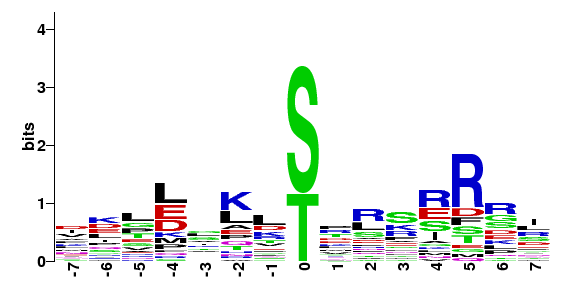

Supplement: Logos of 300 phosphorylation motifs predicted [file msb201312-s6.zip › Logo/EIF2AK2.png]

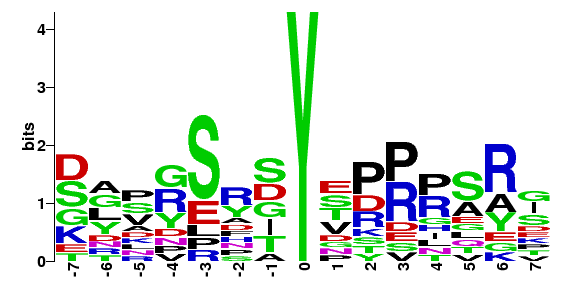

Supplement: Logos of 300 phosphorylation motifs predicted [file msb201312-s6.zip › Logo/EPHA3.png]

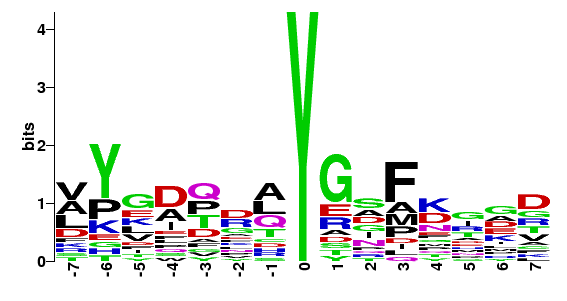

Supplement: Logos of 300 phosphorylation motifs predicted [file msb201312-s6.zip › Logo/EPHB3.png]

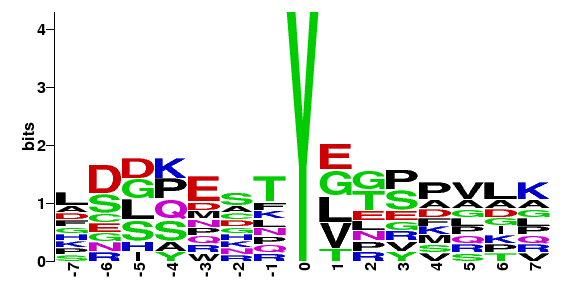

Supplement: Logos of 300 phosphorylation motifs predicted [file msb201312-s6.zip › Logo/EPHB4.png]

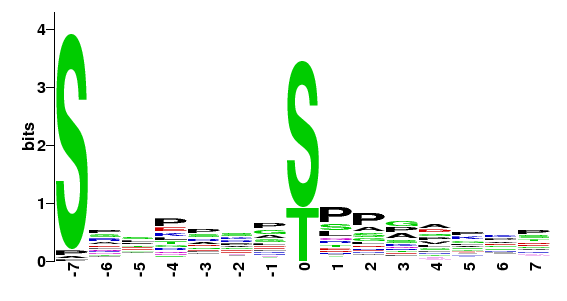

Supplement: Logos of 300 phosphorylation motifs predicted [file msb201312-s6.zip › Logo/FASTK.png]

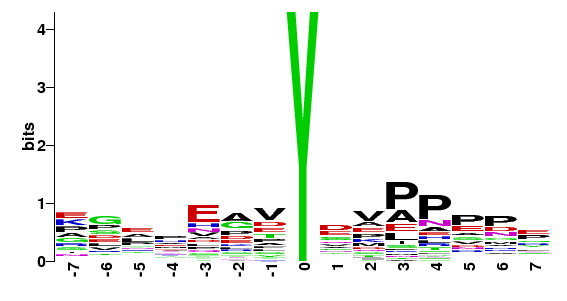

Supplement: Logos of 300 phosphorylation motifs predicted [file msb201312-s6.zip › Logo/FGFR1.png]

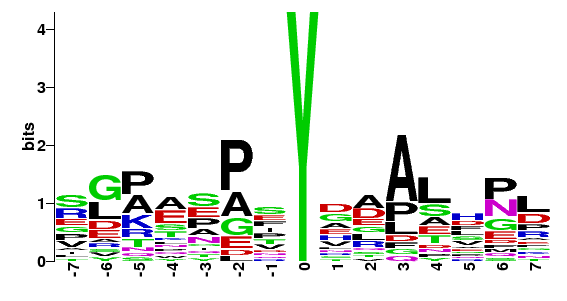

Supplement: Logos of 300 phosphorylation motifs predicted [file msb201312-s6.zip › Logo/FGFR2.png]

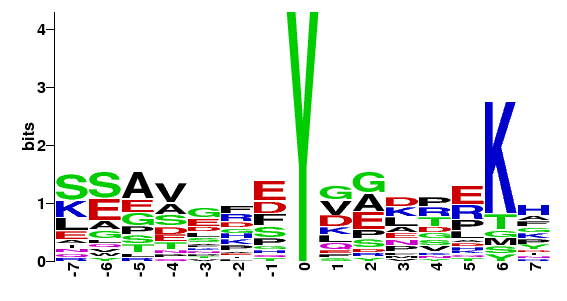

Supplement: Logos of 300 phosphorylation motifs predicted [file msb201312-s6.zip › Logo/FGFR4.png]

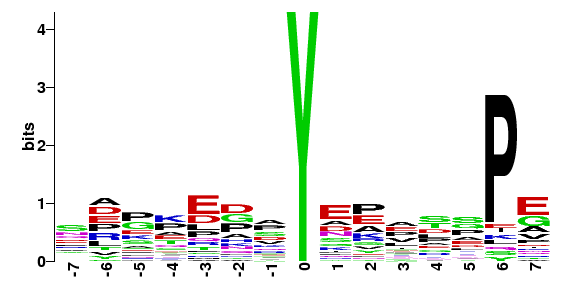

Supplement: Logos of 300 phosphorylation motifs predicted [file msb201312-s6.zip › Logo/FGR.png]

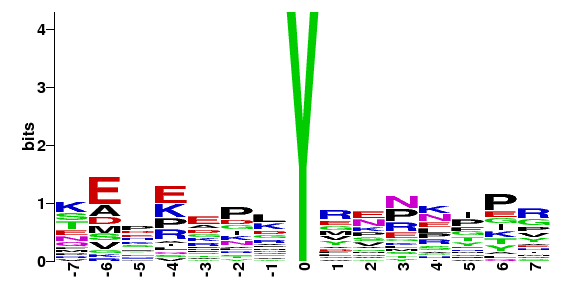

Supplement: Logos of 300 phosphorylation motifs predicted [file msb201312-s6.zip › Logo/FRK.png]

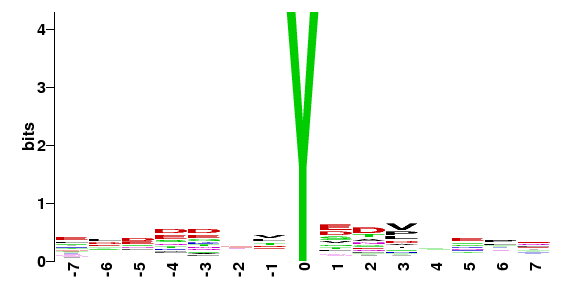

Supplement: Logos of 300 phosphorylation motifs predicted [file msb201312-s6.zip › Logo/FYN.png]

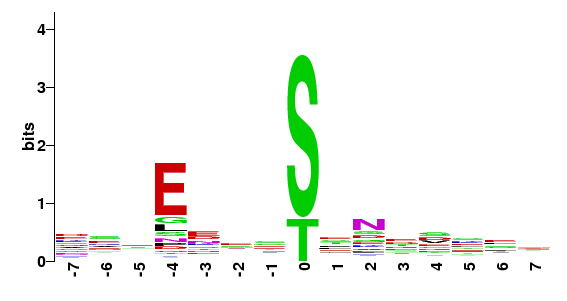

Supplement: Logos of 300 phosphorylation motifs predicted [file msb201312-s6.zip › Logo/GRK5.png]

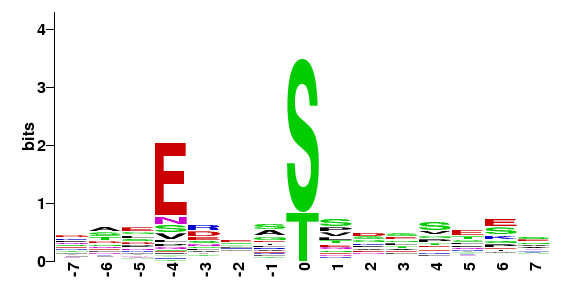

Supplement: Logos of 300 phosphorylation motifs predicted [file msb201312-s6.zip › Logo/GRK6.png]

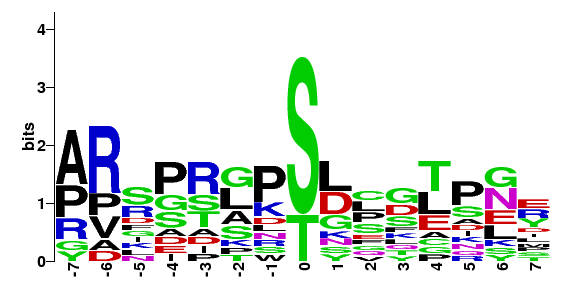

Supplement: Logos of 300 phosphorylation motifs predicted [file msb201312-s6.zip › Logo/GSG2.png]

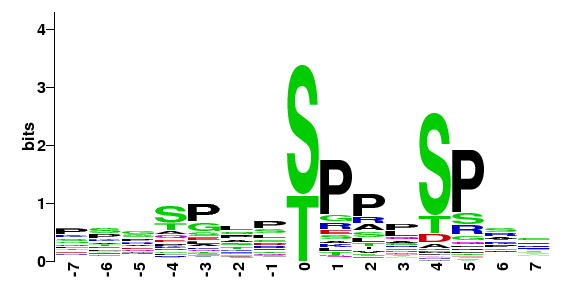

Supplement: Logos of 300 phosphorylation motifs predicted [file msb201312-s6.zip › Logo/GSK3A.png]

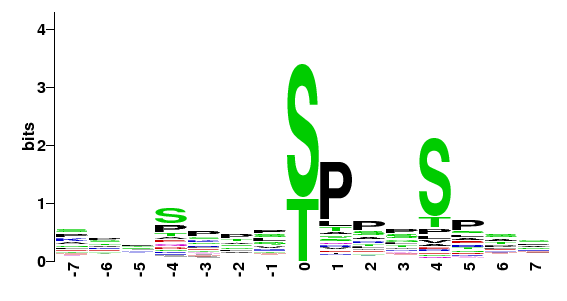

Supplement: Logos of 300 phosphorylation motifs predicted [file msb201312-s6.zip › Logo/GSK3B.png]

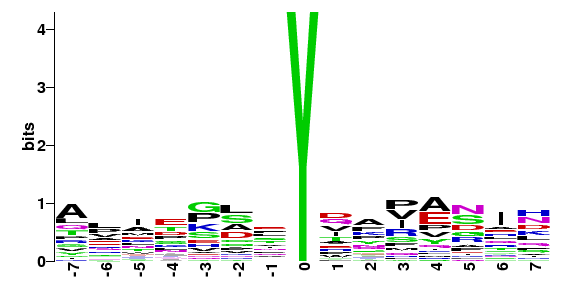

Supplement: Logos of 300 phosphorylation motifs predicted [file msb201312-s6.zip › Logo/HCK.png]
